# Lasting Lower Rhine-Meuse forager ancestry shaped Bell Beaker expansion

**DOI:** 10.1038/s41586-026-10111-8

**Published:** 2026-02-11

**Authors:** Iñigo Olalde, Eveline Altena, Quentin Bourgeois, Harry Fokkens, Luc Amkreutz, Steffen Baetsen, Marie-France Deguilloux, Alessandro Fichera, Damien Flas, Francesca Gandini, Jan F. Kegler, Lisette M. Kootker, Judith van der Leije, Kirsten Leijnse, Constance van der Linde, Leendert Louwe Kooijmans, Roel Lauwerier, Rebecca Miller, Helle Molthof, Pierre Noiret, Daan C. M. Raemaekers, Maïté Rivollat, Liesbeth Smits, John R. Stewart, Theo ten Anscher, Michel Toussaint, Kim Callan, Olivia Cheronet, Trudi Frost, Lora Iliev, Matthew Mah, Adam Micco, Jonas Oppenheimer, Iris Patterson, Lijun Qiu, Gregory Soos, J. Noah Workman, Ceiridwen J. Edwards, Iosif Lazaridis, Swapan Mallick, Nick Patterson, Nadin Rohland, Martin B. Richards, Ron Pinhasi, Wolfgang Haak, Maria Pala, David Reich

**Affiliations:** 1 Department of Zoology and Animal Cell Biology, University of the Basque Country UPV/ EHU, Vitoria-Gasteiz, Spain; 2 Department of Human Evolutionary Biology, Harvard University, Cambridge, MA, USA; 3 Ikerbasque–Basque Foundation of Science, Bilbao, Spain; 4 Department of Human Genetics, Leiden University Medical Center, Leiden, The Netherlands; 5 Faculty of Archaeology, Leiden University, Leiden, The Netherlands; 6 National Museum of Antiquities, Leiden, The Netherlands; 7 Archeologisch Onderzoek Leiden BV, Leiden, The Netherlands; 8 PACEA UMR 5199, University of Bordeaux, Pessac, France; 9 School of Applied Sciences, University of Huddersfield, Huddersfield, UK; 10 Service de Préhistoire, Université de Liège, Liège, Belgium; 11 LAMPEA, Aix-Marseille Université, Aix-en-Provence, France; 12 Department of Archaeology, Ostfriesische Landschaft, Aurich, Germany; 13 Faculty of Science, Department of Earth Sciences - Isotope Archaeology, Vrije Universiteit Amsterdam, Amsterdam, The Netherlands; 14 BAAC archaeology, Hertogenbosch, The Netherlands; 15 Tot op het Bot, Amsterdam, The Netherlands; 16 Formerly Cultural Heritage Agency of the Netherlands, Ministry of Education, Culture and Science, The Netherlands; 17 RAAP, Weesp, The Netherlands; 18 University of Groningen, Groningen Institute of Archaeology (GIA), Groningen, The Netherlands; 19 Department of Archaeogenetics, Max Planck Institute for Evolutionary Anthropology, Leipzig, Germany; 20 ArcheOs, Research Laboratory for Biological Anthropology, Department of Archaeology, University of Ghent, Ghent, Belgium; 21 Faculty of Humanities, University of Amsterdam, Amsterdam, The Netherlands; 22 Faculty of Science and Technology, Bournemouth University, Poole, Dorset, UK; 23 Department of Genetics, Harvard Medical School, Boston, MA, USA; 24 Department of Evolutionary Anthropology, University of Vienna, Vienna, Austria; 25 Howard Hughes Medical Institute, Harvard Medical School, Boston, MA, USA; 26 Broad Institute of MIT and Harvard, Cambridge, MA, USA; 27 Human Evolution and Archaeological Sciences, University of Vienna, Vienna, Austria

## Abstract

Ancient DNA studies revealed that in Europe from 6500-4000 BCE, descendants of western Anatolian farmers mixed with local hunter-gatherers resulting in 70-100% ancestry turnover^1^, then steppe ancestry spread with the Corded Ware complex 3000-2500 BCE^2^. We document an exception in the wetland, riverine, and coastal areas of the Netherlands, Belgium, and western Germany, using genome-wide data from 112 people 8500-1700 BCE. Here, a distinctive population with high (~50%) hunter-gatherer ancestry persisted three thousand years later than in most European regions, reflecting incorporation of females of Early European Farmer ancestry into local communities. In the western Netherlands, the arrival of the Corded Ware complex was also exceptional: lowland individuals from settlements adopting Corded Ware pottery had hardly any steppe ancestry, despite a Y-chromosome characteristic of early Corded Ware-associated people. These distinctive patterns may reflect the specific ecology they inhabited, which was not amenable to full adoption of the early Neolithic type of farming introduced with Linearbandkeramik^3^, and resulted in distinct communities where transfer of ideas was accompanied by little gene flow. This changed with the formation of Lower Rhine-Meuse Bell Beaker users by fusion of local people (13-18%) and Corded Ware associated migrants of both sexes. Their subsequent expansion then had a disruptive impact across a much wider part of northwestern Europe, especially in Great Britain where they were the main source of a 90-100% replacement of local Neolithic ancestry.

Whole genome ancient DNA (aDNA) analysis has illuminated long-standing debates about cultural and demographic transformations in Holocene Europe. Two major prehistoric events have been characterized: the spread of genetic ancestry originating from western Anatolian farmers into Europe associated with the introduction of farming in the Early Neolithic^4^, and the spread of ancestry characteristic of Yamnaya steppe pastoralists during the 3rd millennium BCE^2,5–7^, mediated by the dispersal of the Corded Ware (CW) and Bell Beaker (BB) complexes. However, the demographic processes on a regional level are still not clearly understood and have been shown to follow variable patterns. For example, while the spread of Anatolian ancestry in central Europe was primarily propelled by the expansion of Linearbandkeramik (LBK) farmers^1,4,5^, in the Baltic region and Scandinavia adoption of the farming lifestyle took place much later and in some cases, there was even a return to hunting, gathering, and fishing^8,9,10^.

This paper focuses on the unique trajectory of communities from water-rich environments in the wider Lower Rhine-Meuse area in western and central Netherlands, Belgium, and northern and northwestern Germany. Around 5500 BCE, the southern part of this region witnessed the arrival of LBK-associated farmers, who settled across the fertile loess soils in the south of the Netherlands and parts of Belgium, Germany and France. Within these communities, there is evidence of contact with hunter-gatherer groups, as documented by Limburg and La Hoguette pottery^11^, although the origin of these ceramics and significance^12^ of these contacts are debated^3^. Once established, these LBK communities developed into regional variants such as the Blicquy, Rössen, Villeneuve-Saint-Germain, Bischheim, and the later southern Michelsberg groups.

North of the loess, large rivers such as the Scheldt, Meuse, and Rhine created a dynamic landscape that included fertile soils favoured by farmers, alongside coastlines, beach barriers, river delta wetlands and forested river dunes that continued to support hunting, gathering and fishing practices after the full adoption of farming around 4200 BCE^3,13–16^. This contrasts with other areas of Europe (with the exception of northern Scandinavia, the Baltic region and the eastern European taiga), where farming practices quickly became dominant^8^. In the Lower Rhine-Meuse area, the wetland communities of the Swifterbant (5^th^ millennium BCE) and Hazendonk cultures (4000-3500 BCE) settled on elevated areas (river and coastal dunes, crevasse splays, and river levees) in a region dominated by water courses and peat bogs. They relied mostly on hunting, gathering, and fishing, but also practiced farming. Around 3500 BCE, the Vlaardingen culture succeeded the Swifterbant/Hazendonk tradition, while remaining settled in approximately the same region^17^. Simultaneously, farmers associated with the Funnelbeaker culture (or TRB for *Trechterbekercultuur* in Dutch) settled on the Friesian-Drenthian plateau in the northeast and its surrounding sandy uplands, in regions where no evidence of earlier habitation, neither burials nor settlements, has been found. The Swifterbant, Hazendonk and Vlaardingen settlements were all located near water streams, while TRB farmers settled mostly on forested sandy plateaus and their fringes, as did the Michelsberg communities to the south.

A mixed subsistence strategy of hunting, gathering, and farming persisted in the western/central Netherlands until the 3rd millennium BCE, when a more intense farming-based economy emerged in association with the Late Vlaardingen complex and the introduction of the ard plough around 3000 BCE^18^. The spread of CW influence to the wider Lower Rhine-Meuse area was more complex than in many other areas of central and eastern Europe. In the uplands, where skeletal material tends to be poorly preserved and no ancient DNA data are available, the complete CW package emerged as marked by the construction of CW burial mounds, the general absence of settlements, and sparse pottery finds^19^. By contrast, in wetland areas along the coast, the Rhine-Meuse delta^20^, and other low-lying regions^21^, CW-associated pottery was incorporated into Vlaardingen settlement contexts, but the characteristic CW-style burials were not^20,22,23^.

The arrival of the BB complex around 2500 BCE marked another major cultural transition, as settlements spread across the wetlands and coastal areas, replacing Vlaardingen/CW settlements, though generally not using the same sites^21^. The BB economy was similar to the previous CW one and consisted of predominantly farming mixed with low-intensity hunting and gathering. In the sandy uplands, there was a continuation of the barrow ritual, but with distinct BB characteristics and material culture replacing the CW repertoire^19,24^. BB groups were also well attested south of the Rhine, as evident in BB burial mounds on the sandy soils of the southern Netherlands and Belgium^25–27^. BB settlement sites remain just as elusive in this area as CW settlements. However, the presence of ploughland dated to the Late Neolithic suggests that the lack of settlement evidence is not the result of nomadism but rather of settlements in lower lying places where there is little chance for detection by archaeologists^21^.

Archaeogenetic data have the potential to deepen our understanding of the nature of the dynamic changes in the Lower Rhine-Meuse region. We generated genome-wide data using in-solution enrichment for more than a million single nucleotide polymorphisms (SNPs) from 44 individuals dated between 8500-1700 BCE, sampling cultural contexts that fill gaps in the ancient DNA record of this region (Figure 1a–b; Supplementary Table 1–2). The mean number of SNPs covered from a core set of 1.15 million autosomal targets is 492,551, with a mean coverage of 1.09. Together with 69 published individuals^1,6,28–31^, the time-transect includes 112 individuals. We also report 14 new direct radiocarbon dates on newly analyzed individuals (Supplementary Table 16).

## Late persistence of forager ancestry

**Figure 1.**
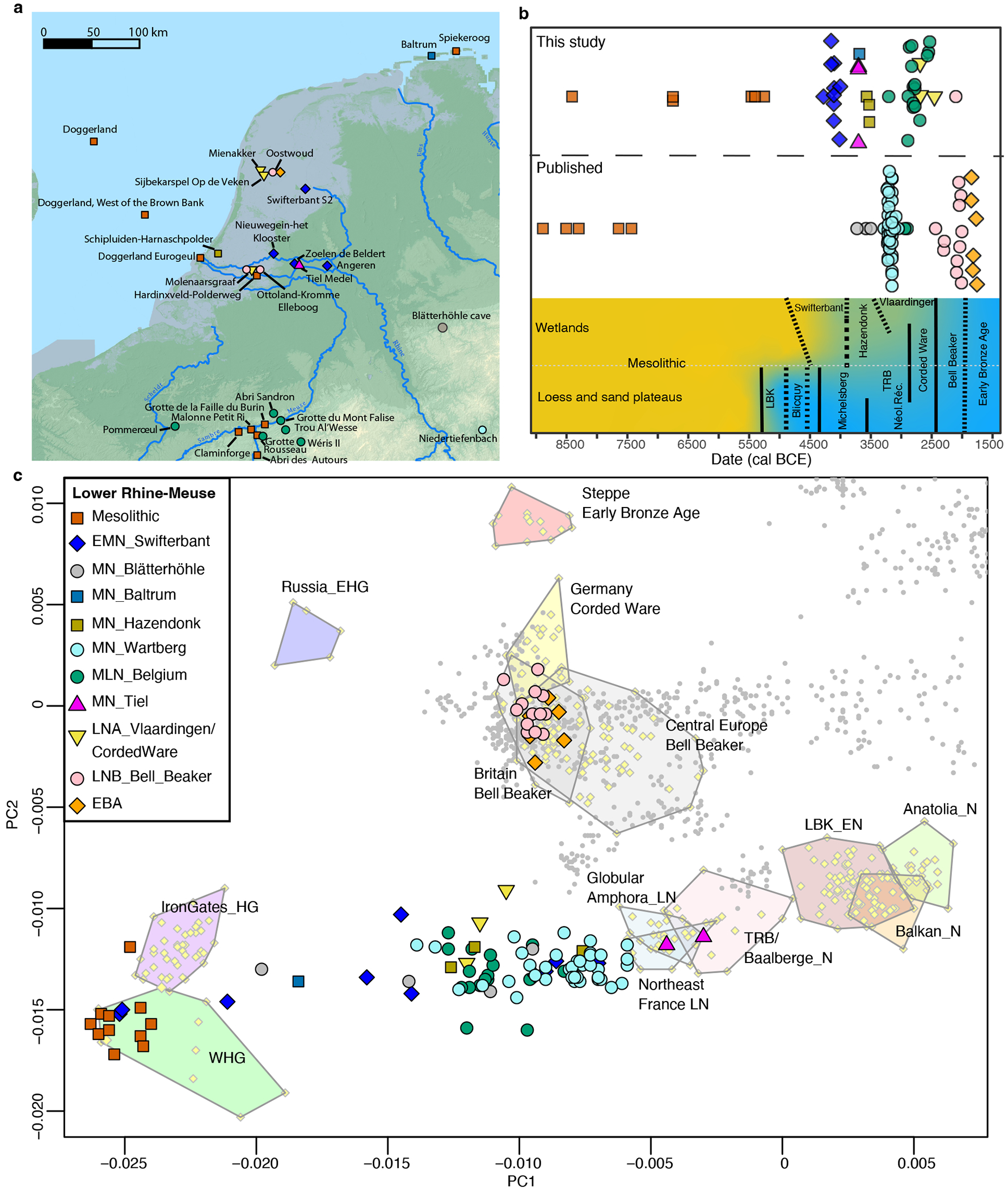
Overview of ancient individuals analyzed in this study. **a)** Map showing archaeological sites with genome-wide data in the Lower Rhine-Meuse area and adjacent regions. The elevation map was downloaded from https://www.mapsforeurope.org/datasets/euro-dem. **b)** Chronological placement of the individuals from the Lower Rhine-Meuse region included in this study. In the bottom panel the local chronology of archaeological cultures. Black lines indicate the degree of changes, with dashed lines representing a gradual change compared to a solid line indicating a more abrupt change in material culture. The colour gradient indicates the general reliance on hunting and gathering (yellow) to farming (blue). **c)** Principal Component Analysis with the ancient individuals projected onto the principal components computed on present-day individuals from West Eurasia. EMN, Early-Middle Neolithic; MLN, Middle-Late Neolithic; EN, Early Neolithic; MN, Middle Neolithic; LN, Late Neolithic; LNA, Late Neolithic A; LNB, Late Neolithic B; N, Neolithic; EBA, Early Bronze Age; WHG, Western hunter-gatherers; IronGates_HG, Iron Gates hunter-gatherers; EHG, Eastern hunter-gatherers.

We report 5 new Mesolithic individuals who traced all their ancestry from Mesolithic western hunter-gatherers (WHG) (Supplementary Table 5), matching previous genetic results from Mesolithic hunter-gatherers from the region^28^. Based on Principal Component Analysis (PCA) (Figure 1c), Neolithic individuals from the Lower Rhine-Meuse area fall along the central/western European Neolithic cline, but much closer to WHG than most European Neolithic farmers. This suggests elevated WHG-related ancestry, which we confirmed through modelling using *qpAdm* (Supplementary Table 6*)*. We find that the earliest Neolithic individuals (4400-3800 BCE), associated with the Swifterbant culture, are genetically highly heterogeneous, with a mother and her daughter (I12093-I12094; Nieuwegein-het Klooster) entirely descending from hunter-gatherer populations, one individual (I38442 from Angeren-Kampsepad) with 84% of such ancestry; three individuals (I12091-I17968 from Nieuwegein-het Klooster and I33739 from Zoelen-de Beldert) with 60-63% and four individuals (SWA001-SWA002-SWA004 from Swifterbant-S2 and I33738 from Zoelen-de Beldert) with 37-45% (Extended Data Figure 2; Supplementary Table 6). These results differ from the overall patterns of hunter-gatherer and farmer admixture elsewhere in central and western Europe, where the arrival of a farming economy generally reduced local WHG ancestry to <30%. However, the results perhaps make more sense in light of the equally limited economic transformation, which combined farming with continued core reliance on the rich wild resources from the Lower Rhine-Meuse wetlands and river valleys. Genetic mixing of local groups with high WHG ancestry continued for the next ~1500 years, with stable proportions of ~40-50 % WHG and 50-60% early European farmer (EEF) ancestry. Rare exceptions include one Middle Neolithic individual from the island of Baltrum (BLR001) and one individual from the Blätterhöhle cave (I1565^1^), both with >75% hunter-gatherer ancestry. The fact that this relatively high WHG ancestry extended not just to the Lower Rhine-Meuse wetlands, but also further along the Rhine and Meuse rivers and the northern coast, is consistent with archaeological evidence of continued cultural engagement of people across this region^32^. Three individuals from Tiel-Medel-de Roeskamp who can be indirectly dated to ~3700 BCE (Supplementary SI 2; Supplementary Table 14), deviate from this pattern of high WHG ancestry, with only ~20% (Extended Data Figure 2), possibly representing new arrivals from neighboring parts of northwest Europe with lower WHG-associated ancestry. Their distinct genetic profile, in combination with parallels in pottery and lithic technology^32^, suggests an origin to the southeast among contemporaneous fully Neolithic communities in that region such as Bischheim groups. As such the Tiel-Medel-de Roeskamp settlement represents a regional outlier, both in ancestry and material culture, and highlights that the Lower Rhine-Meuse area was not isolated, but part of a dynamic frontier characterised by mobility, encounter, and interaction across cultural boundaries.

**Figure 2.**
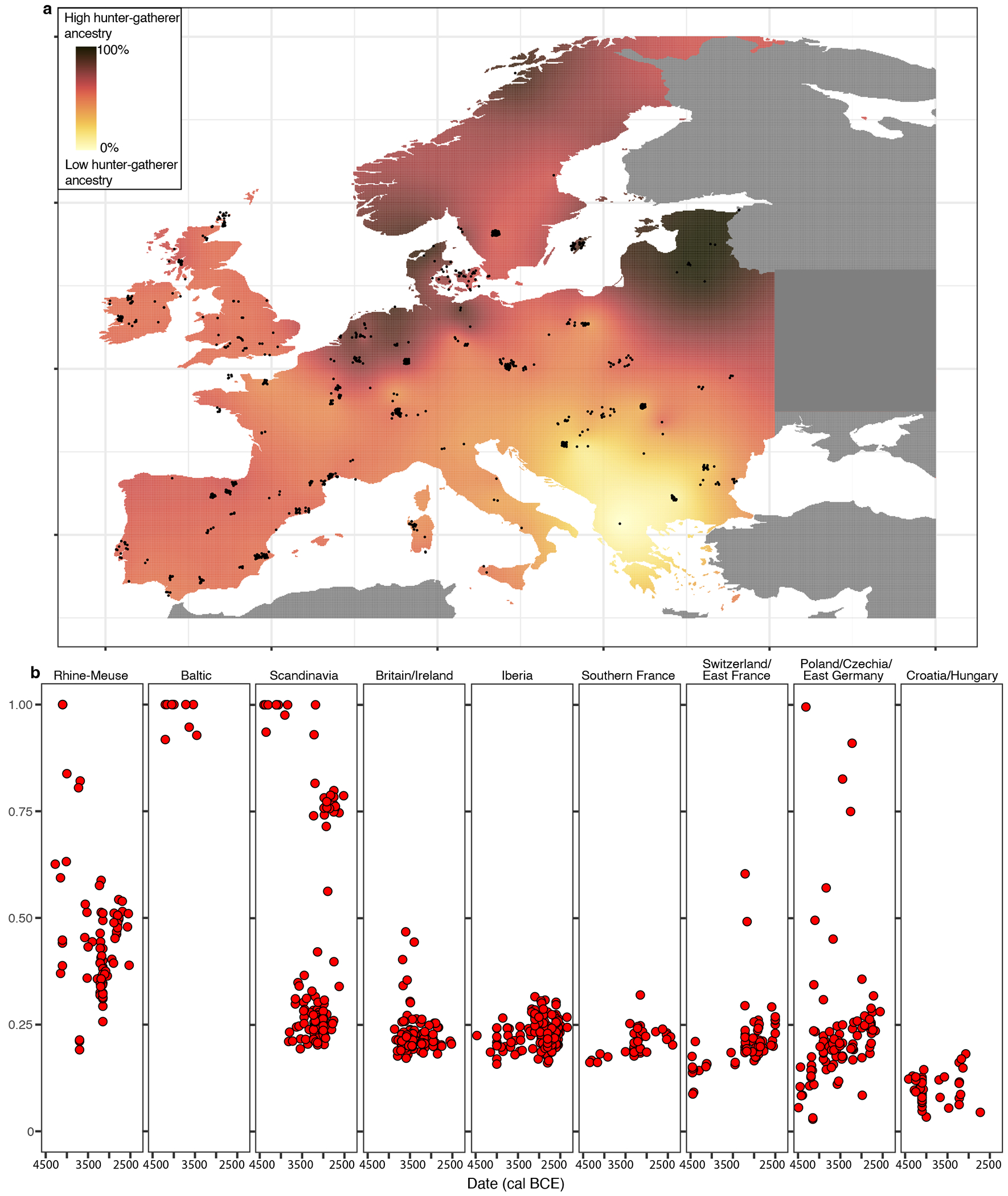
Hunter-gatherer ancestry proportions across Europe between 4500-2500 BCE. **a)** Spatial kriging of hunter-gather ancestry. The colors represent the predicted ancestry proportion at each point in the grid. Map data from the R package ‘maps’. **b)** Hunter-gatherer ancestry levels in individuals from different European regions. Ancestry proportions were estimated with *qpAdm* (Supplementary Tables 6 and 7).

Compared to other regions of central, southern, and western Europe where farming was practiced, the Lower Rhine-Meuse area stands out for its long survival of high proportions of WHG-related ancestry on a population scale (as opposed to isolated cases^33–35^) until the BB transition, halfway through the 3^rd^ millennium BCE (Figure 2). To identify other instances where WHG ancestry on a population scale endured in such high proportions to the dawn of the Bronze Age, it is necessary to go to parts of the Baltic coast where populations with high EEF ancestry never made significant impact^9^, and to Scandinavia where hunter-gatherers with full WHG ancestry persisted until the early 3^rd^ millennium BCE alongside EEF ancestry-rich farmers^10^ (Figure 2; Supplementary Table 7).

The unique ancestry makeup of Lower Rhine-Meuse Neolithic groups is also evident from their EEF-WHG admixture time estimates (Extended Data Figure 4), which point to ongoing admixture well into the fourth millennium BCE, unlike other European regions (Supplementary Table 15). The Tiel-Medel-de Roeskamp individuals represent a deviation from this pattern with older admixture dates, again highlighting their likely recent origin outside the Lower Rhine-Meuse area.

## Female-mediated Early Farming ancestry

We find that the EEF ancestry proportions in Lower Rhine-Meuse area Neolithic people were significantly higher on chromosome X than the autosomes (normally distributed Z-score=5) (Supplementary Table 8), indicating a higher ancestral contribution from women with EEF ancestry. Independent confirmation is provided by analysis of the two uniparentally inherited parts of the genome (Supplementary Table 13). Among the Early and Middle Neolithic men (*n*=43 excluding close relatives) we only observed Y-chromosome lineages common in Mesolithic hunter-gatherers (haplogroups I2a, R1b-V88 and C1a2). In contrast, the maternally transmitted mitochondrial lineages are predominantly of Neolithic farmer origin (50 out of 71, based on their absence in sampled European Mesolithic individuals^4,6,9,10,28,35–37^. For example, the earliest individual with EEF ancestry, a female associated with the Swifterbant culture and dated to ~4342-4171 cal BCE (I17968, Nieuwegein-het Klooster) at the start of the transition to farming in the region^14,16^, harbors only 37% EEF ancestry in her autosomes but farmer-associated mitochondrial haplogroup H+152. A previous study^36^ reported similar sex-biased admixture in Neolithic farmers of Iberia and in Funnel Beaker farmers of northern Europe. A plausible scenario is that in all three regions, hunter-gatherer communities incorporated farmer women, who plausibly mediated the exchange of ideas and technologies related to farming. This scenario of sex-biased admixture of Neolithic ancestry contrasts with one of almost complete displacement of local ancestry by incoming farmers and migration of entire groups, a process that occurred in other parts of early Neolithic Europe^38^.

The Middle Neolithic populations of the Lower Rhine-Meuse region were highly genetically interconnected, as reflected in large segments (>12 cM) of the genome being identical by descent (IBD), which is only expected to be observed for individuals who share common ancestors in the last dozens of generations^39^ (Supplementary Table 14). We also find several cases of IBD segments >20 cM, suggesting even closer relationships between sites such as Blätterhöhle, Niedertiefenbach, and Abri Sandron, as well as between sites in the Lower Rhine-Meuse area and nearby areas of central Europe and Northern France (Figure 4a). A striking case is a relationship (~50 cM in IBD) between an individual from Blätterhöhle, modern western Germany, and a father-daughter pair from Mont-Aimé^34^ in modern northern France, who are also clear ancestry outliers exhibiting more hunter-gatherer ancestry than other individuals from Mont-Aimé.

## CW using groups with minimal steppe DNA

**Figure 3.**
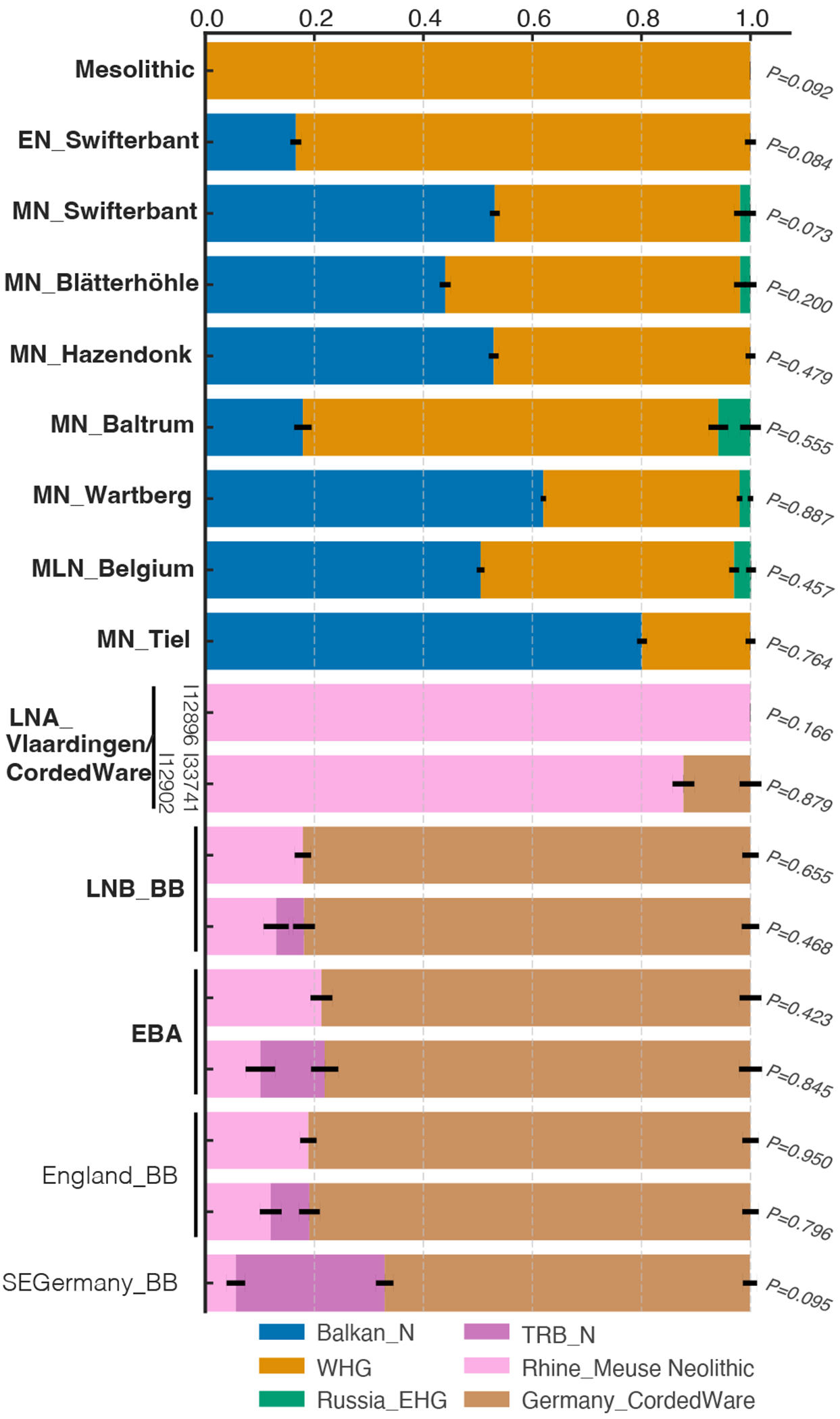
Admixture proportions for Lower Rhine-Meuse populations and other relevant groups. Admixture proportions were estimated with *qpAdm* (Supplementary Tables 5,6 and 10). In bold, groups from the Rhine-Meuse region. Error bars indicate the standard error of estimates from 5-cM-block jackknife analysis. *P* values for the fit of each *qpAdm* model to the genetic data are provided.

In many areas of Europe, the emergence of the CW complex is associated with large-scale demographic change due to the arrival of groups carrying steppe ancestry. The ancestry change in three sampled individuals from Vlaardingen/CW contexts in the Western Netherlands is far smaller. These individuals were buried within settlements with CW complex material goods, but not the typical CW single grave burials, which are overall absent from the Vlaardingen culture region. One female (I12896 from Molenaarsgraaf-24A) has no steppe-related ancestry at all and instead shares ancestry with local late Neolithic farmers of the region (Figure 3; Supplementary Table 9). However, the other two individuals (I12902 and I33741) from Opmeer-Mienakker and Sijbekarspel-Op de Veken, north of the Rhine River delta, can be modeled as a mixture of 12-16% ancestry associated with the main cluster of CW groups, and 84-88% derived from Lower Rhine-Meuse area Neolithic populations with high hunter-gatherer ancestry, similar to Late Neolithic Belgium or Hazendonk (Figure 3; Supplementary Table 10). Despite a low steppe ancestry proportion in the autosomes, the male I12902 from Opmeer-Mienakker carries Y haplogroup R1b-U106, also known in one early CW-associated individual from Bohemia^7^. Furthermore, one of his bones yields a date of 2852-2574 cal BCE; one of the earliest CW complex associated dates in Europe outside of Bohemia and the Baltic region^40^. These results suggest that the male ancestor who brought this Y haplogroup to the Lower Rhine-Meuse region was part of the early CW expansion.

While limited to three people, the IBD analysis for the Vlaardingen/CW individuals revealed two additional notable signals. First, the two individuals with ~11% CW ancestry, excavated at nearby sites, have an IBD match that represents approximately a 7^th^ degree relation (Supplementary Table 14), hinting at a small community size. Second, the geographical range of their IBD links extends much further east than previous groups (Extended Data Figure 3). Among their closest connections we found a Yamnaya-associated individual from Samara in far eastern Europe^41^ (ID: I6733) and CW-associated individuals from present-day Poland^42^ (ID: pcw362) and Estonia^43^ (ID: NEO306), with whom Vlaardingen/CW individuals share one IBD segment of ~19 cM long that are very unlikely to survive for more than a handful of generations. We also detect connections to other Lower Rhine-Meuse area Late Neolithic individuals (Supplementary Table 14), providing direct evidence of a major local ancestry component in Vlaardingen/CW individuals. These patterns reflect their dual sources of ancestry: a minor component (potentially completely along the male line) from central/eastern CW groups (and through them to Yamnaya steppe pastoralists) and a major component from the local Neolithic population. Although our individuals do not represent the initial generation of admixture between CW-related and local groups, patterns from nearby northeastern France could serve as a good model for this interaction. There, ~2500 BCE, a man with ancestry similar to CW-associated individuals (including nearby CBV95^44^ buried with an All Over Ornamented CW type Beaker) fathered a child with a local woman^45^. His son did the same, rapidly reducing steppe ancestry to levels comparable to those seen in the Vlaardingen/CW individuals I12902 and I33741.

## Bell Beaker users had local admixture

With the advent of the BB complex in the Lower Rhine-Meuse delta after 2500 BCE we see a strong difference in genetic ancestry compared to the previous CW/Vlaardingen individuals. All 13 available BB-associated individuals appear genetically close to eastern European CW-associated groups, but not to the preceding Vlaardingen/CW individuals in the PCA (Figure 1c). They can be modeled with ~82% ancestry from the main cluster of eastern European CW-associated individuals (Figure 3; Supplementary Table 10), and their remaining ancestry from a Wartberg Neolithic-related group (Supplementary Table 11), representing the Neolithic population from the Lower Rhine-Meuse area with the lowest level of hunter-gatherer ancestry, or as a mixture between Middle and Late Neolithic groups from outside the Lower Rhine-Meuse area (*e.g.* Poland Globular Amphora, Czechia TRB, Germany Baalberge, Iberia Neolithic-Chalcolithic) and Late Neolithic populations from Belgium. All these scenarios point to a ~82-87% (but not 100%) ancestry change associated with the arrival of the BB complex in the Lower Rhine-Meuse region (Supplementary Table 10). A contribution of local farmers, with their distinctive signature of high hunter-gatherer ancestry, is essential to model the formation of Lower Rhine-Meuse delta BB-associated individuals. Even at 13-18%, we can be confident this local admixture occurred: models lacking this unique Lower Rhine-Meuse farmer genetic contribution are rejected with strong statistical significance (Supplementary Table 10). This suggests that the observed mixture between CW culture associated groups and European farmers that formed the genetic profile of Lower Rhine-Meuse BB must have occurred, at least in part, in the region itself. In the context of these genetic results, it is notable that radiocarbon dates suggest that the Lower Rhine-Meuse area was one of the earliest places where the BB cultural phenomenon arose^46^. While the earliest appearance of BB cultural material has been located in Iberia^47^, our results show that the earliest formation of BB-associated groups that were influenced not just culturally but also genetically by CW users may have occurred in western Europe including the Lower Rhine-Meuse area.

Because there are possibly several centuries between the analyzed CW/Vlaardingen and BB populations we need to consider the option that the drastic change in genetic ancestry could have been a more gradual process, rather than a sudden change. What is different, though, compared to the previous ancestry changes in our time transect, is that it involved both sexes this time. Among BB men, all yielded R1b-L151 haplogroups, which were absent in earlier Neolithic European populations, except for early CW-associated individuals from Czechia in central Europe^7^. All nine available BB-associated men from Oostwoud-Tuithoorn and Ottoland-Kromme Elleboog belonged to the derived R1b-L151P312 lineage (Supplementary Table 1), the major lineage among BB groups across Europe^6^. Three P312 individuals could be further subtyped to DF19, a minor P312 subtype today mostly present in central/northern European populations (https://www.yfull.com/tree/R-DF19/). At Molenaarsgraaf, the only man with enough resolution to determine an L151 subtype belonged to R1b-L151-U106 (I13025), matching the Vlaardingen/CW associated male from Opmeer-Mienakker, referred to above, and suggesting a similar CW-related source for the patrilineal ancestry in both Vlaardingen/CW and BB men in the Lower Rhine-Meuse area. This would be consistent with the hypothesis that even if there was limited local continuity within the lowlands, the same male lineages that were associated with the arrival of CW pottery to the region (in the Vlaardingen/CW context) were at least partially associated with subsequent BB emergence. This suggests that the BB associated population in the Lower Rhine-Meuse area could have emerged from sustained influx of ‘classic’ CW-related groups to the region (such as those documented in the uplands to the east, where skeletal preservation is absent but classic CW burial features are present), which mixed with the local Vlaardingen or other Lower Rhine-Meuse Neolithic populations that had high hunter-gatherer ancestry.

**Figure 4.**
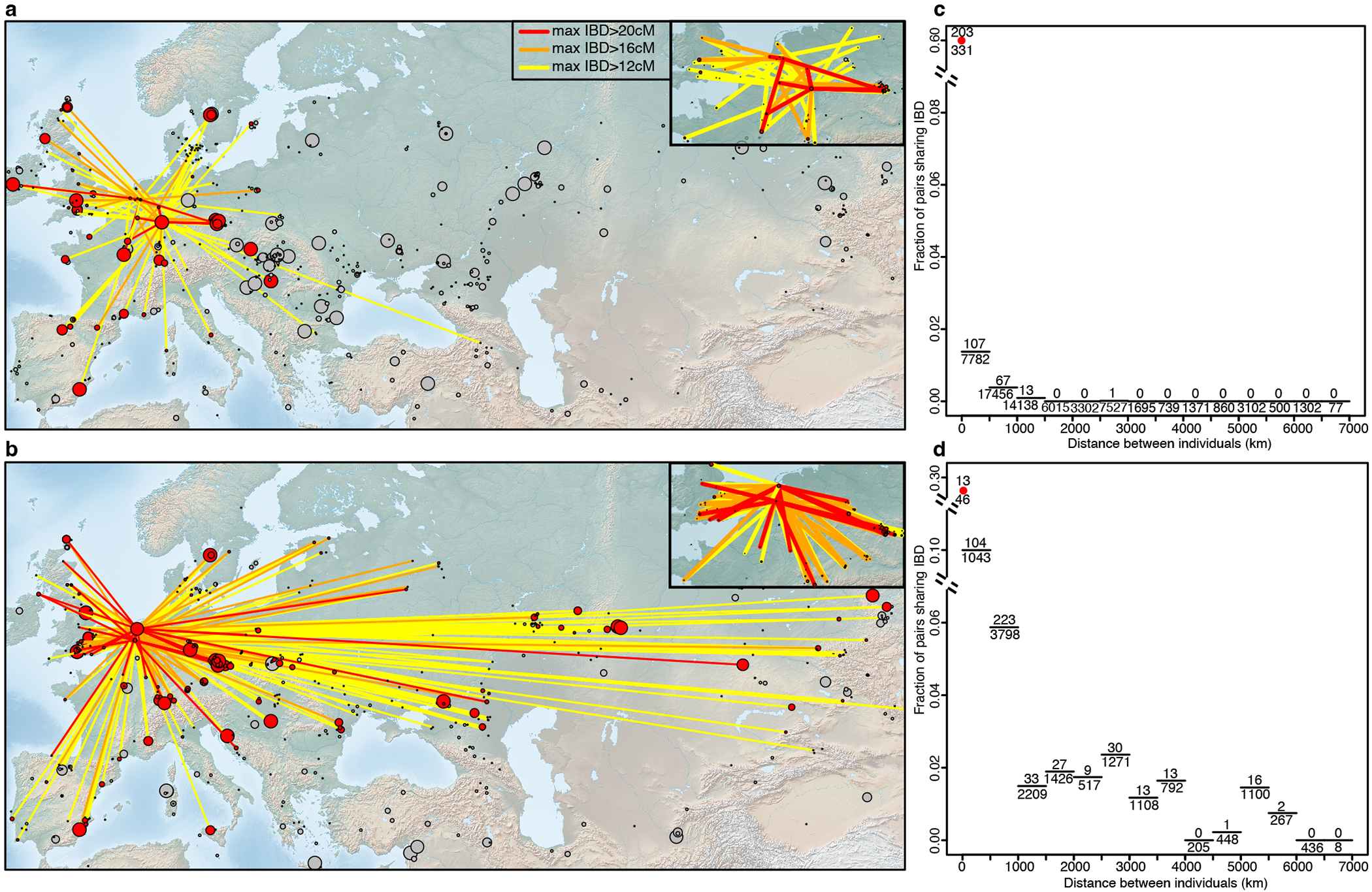
Genetic connections of Lower Rhine-Meuse groups. IBD connections of **a)** Lower Rhine-Meuse Early, Middle and Middle-Late Neolithic individuals (n=35) and **b)** Lower Rhine-Meuse BB-associated individuals (n=11). Sites are represented by circles with size proportional to the number of individuals amenable to IBD calling. Grey circles indicate archaeological sites between 5500-2500 BCE (top) and 3000-1500 BCE (bottom) with no IBD connections to Lower Rhine-Meuse individuals. Decay of IBD sharing with geographic distance for **c)** Lower Rhine-Meuse Early, Middle and Middle-Late Neolithic individuals and **d)** Lower Rhine-Meuse BB-associated individuals. Pairs were considered to share IBD if they share at least one segment of >12cM. Maps were drawn using public-domain Natural Earth data with the rnaturalearth package in R.

Further evidence for a major influx from outside regions during the BB period comes from inspection of IBD networks, which, after this point, expand thousands of kilometers further to the east and northeast compared to the CW/Vlaardingen period (Figure 4b). These show strong links to CW and BB-associated individuals from Bohemia, as well as evidence of distant Early Bronze Age relatives from England and Scotland (Supplementary Table 14), corroborating findings of a shared origin between these groups located on opposite shores of the North Sea^6^. The BB horizon is the first period in our time transect when people of the Lower Rhine-Meuse region became intensively integrated within a much wider European IBD network, in contrast to the previous more regional patterns, yet another indication for the high level of mobility that has become evident from isotope studies^48^.

After this period of profound demographic change, Early Bronze Age individuals from the Lower Rhine-Meuse area had similar ancestry to BB predecessors (Figure 1c; Figure 3), with ~3% additional Neolithic-related ancestry (Supplementary Table 10), potentially reflecting small-scale admixture with neighboring populations. Local continuity from the BB period to the Middle Bronze Age is also reflected in the abundant familial links connecting Early Bronze Age individuals with both earlier BB individuals and later Middle Bronze Age ones (Supplementary Table 14), with pairs sharing up to 100 cM IBD as expected for ~6th-degree relations.

## Lower Rhine-Meuse expansion to Britain

The study identifies the Lower Rhine-Meuse region as the likely origin of a secondary expansion of BB users that had a greater demographic impact than the postulated initial Iberian expansion.

**Extended Data Figure 1.**
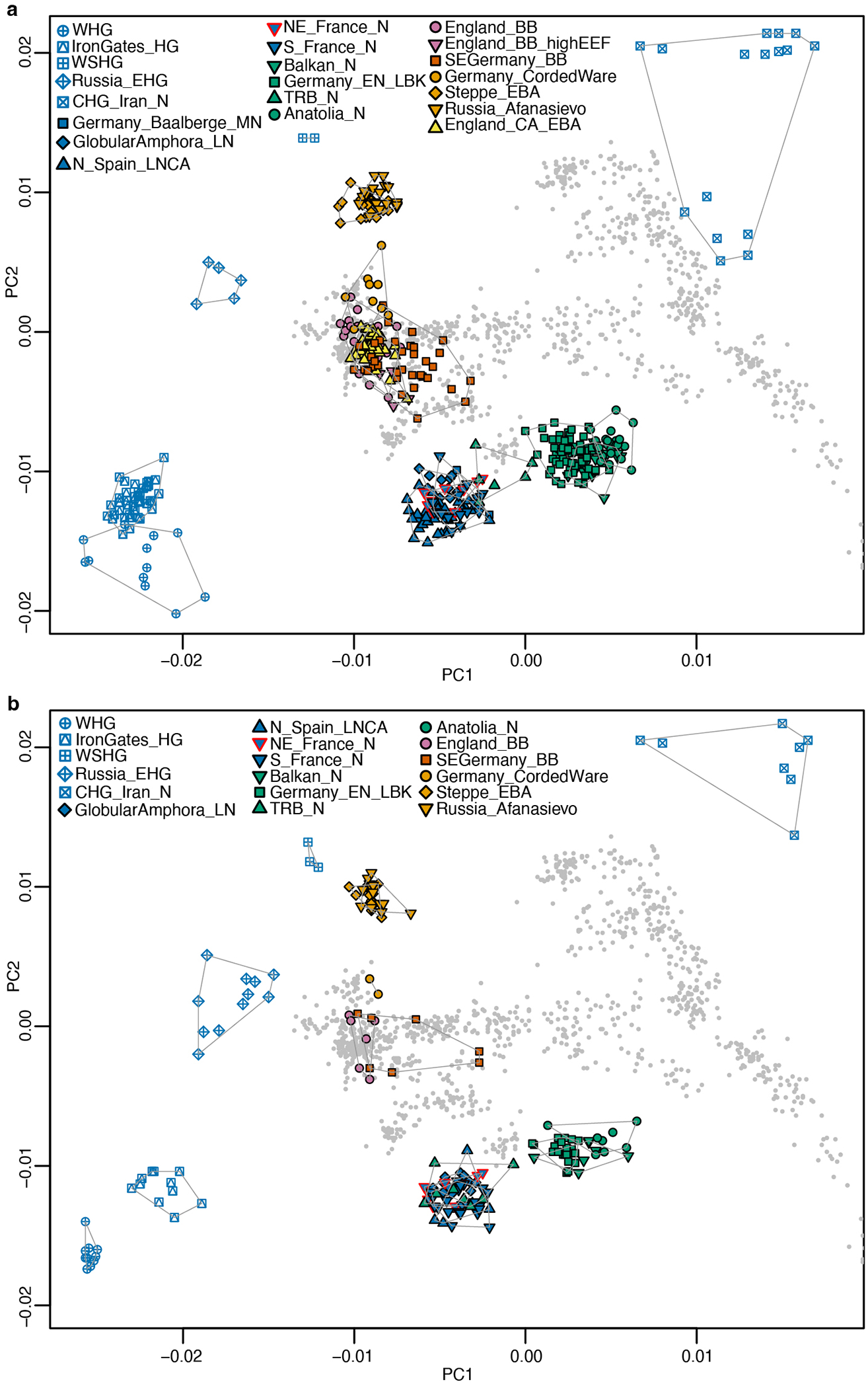
Genetic structure of relevant ancient groups used in *qpAdm* analysis. PCA of sources and outgroups used under *qpAdm* **a)** setup 1-3 and **b)** setup 4 (Supplementary Tables 3–4).

To document this, we used our statistical modelling framework to re-examine the genetic evidence of the arrival of BB users in Britain. Previous work proposed an origin of British BB-associated groups in the Lower Rhine-Meuse based on Y-chromosome data^7^, and showed a minimum ~90% ancestry change^6^, but had not been able to distinguish models in which the EEF ancestry came from Britain itself or elsewhere. We analyzed 28 BB-associated individuals from Great Britain from the main homogeneous cluster (Supplementary Table 3–4) and obtained the same result as for Rhine-Meuse delta BB individuals (Supplementary Table 10; Table 1). Both require ~12-19% ancestry from Lower Rhine-Meuse Neolithic populations with high levels of hunter-gatherer ancestry, plus ancestry from the main CW genetic cluster (Figure 3). This is consistent with no contribution at all from British Neolithic farmers. However, in an outlier subset of four BB associated individuals from Britain (including the high-status “Amesbury Archer”) with lower proportions of steppe ancestry than the main cluster (Extended Data Figure 1; Supplementary Table 3), models featuring CW Complex associated groups and Lower Rhine-Meuse farmers provide a poor fit (Supplementary Table 10). We cannot rule out the possibility that some of the ancestry of these outliers could derive from local British Neolithic populations, but it could also plausibly come from separate migratory streams into Britain, such as the one suggested for the Amesbury Archer whose isotopic genetic signatures indicate an origin in the Alps^49^. By the Early Bronze Age, when ancestry proportions in Britain stabilized, we estimate ~92% Lower Rhine-Meuse BB ancestry and at most 8% local British Neolithic ancestry but possibly as little as 0% (Supplementary Table 12), providing new information about the magnitude of the demographic transition associated with the BB transition in Britain.

## Discussion

A striking finding in our study is that the long-term persistence of high hunter-gatherer genetic ancestry proportions was not limited to the previously reported Blatterhöhle cave^1^ and Wartberg culture^29^ in the Rhine region but also occurred in the core wetlands of the Netherlands and the inland regions of the Meuse. In the context of archaeological evidence, this suggests the co-existence of two distinct but interdigitated Neolithic spheres in the entire Lower Rhine-Meuse region, persisting well into the 3^rd^ millennium BCE. One of these consisted of communities centered around water, not only those in the wetland core but also connected to it through waterways, and practicing a semi-agrarian lifestyle^3^. Water links were central for these communities, and connections to people living along the waterways were often more important than connections to physically closer neighbors. Surrounding the waterways, the other sphere consisted of early full-blown farming communities that preferred the fertile loess soils and kept to their culturally specific traditions of settlement, housing, material culture, and burial. This supports the “frontier mobility” model proposed by Zvelebil^50^, albeit in a more geographically restricted context. These communities exchanged ideas, with women introducing EEF genetic ancestry and probably also new technological knowledge in the more hunting-gathering practicing communities but their distinct cultures persisted for millennia.

The remarkably high proportion of WHG ancestry was observed from the earliest Neolithic Swifterbant communities to the period of the introduction of the CW complex. CW pottery is present in the Rhine-Meuse delta, but other aspects of this culture are lacking, in particular the characteristic burial rituals^19,20,24^. This is matched by limited population influx and high retention of the genetic ancestry of previously established groups.

The BB complex related communities harbor evidence of a break with the previously established pattern, as they show a profound change in genetic ancestry. This was introduced by new incoming people of both sexes with CW related ancestry. Admixture with local Lower Rhine-Meuse delta populations did occur but was limited to 13-18%. Due to the uncertainty of the radiocarbon dated individuals in our transect we need to consider a potentially slightly larger time-gap between the three analyzed Vlaardingen/CW-associated individuals and the 13 BB-associated individuals. Thus, the time course and exact location in the region where people with a CW-associated genetic profile and a Lower Rhine-Meuse Neolithic genetic profile came together remains unclear. However, the homogeneity of ancestry within the three BB-associated sites we sampled show that the mixture had largely taken place by that time (~2400-1900 BCE).

The evidence for a large-scale demographic change in the Lower Rhine-Meuse region by the time of the spread of Beaker users is important in light of the evidence from Britain, where Beaker users spread at around the same time. Since the British Neolithic populations encountered by Beaker users practiced cremation and thus did not yield samples amenable for aDNA analysis, it has been unclear whether there was a sharp population break or a period of extended co-existence^51^. In the Lower Rhine-Meuse region, we do not have this issue since cremation was rarely practised by previous groups, yet the genetic turnover seems to have been similarly profound as in Britain, with the great majority of local BB burials being consistent with no local British Neolithic ancestry and the contribution of the local British Neolithic population by the Early Bronze Age estimated at ≤9%. While we do not know what triggered this large-scale mobility, it is clear that the genetic legacy of local populations both in the Lower Rhine-Meuse area and Britain collapsed relatively rapidly.

Despite the evidence of a cultural break, in both the Lower Rhine-Meuse area and Britain, local cultural traditions and knowledge remained intact for some time between 2450 and 2200 BCE^49,52,53^. British archaeologists stress that continuity is witnessed in the building and use of Late Neolithic monuments like Stonehenge, Avebury, Woodhenge and Silbury Hill, and that culturally significant changes only occur in the 23^rd^ century BCE^52,54^. Similarly, in the Lower Rhine-Meuse area, BB groups used the same areas, though not the exact same settlement sites as their Vlaardingen/CW predecessors. They continued to settle in river valleys, on crevasse splays, and along river dunes in a way that was oriented explicitly towards a hunting-farming mixed economy. This indicates that the newcomers with their distinct genetic ancestry came from a similar landscape elsewhere or were in close contact with the local communities to learn how to handle this specific type of landscape.

## Methods

### Sampling, extraction, library preparation, capture, sequencing:

Our initial selection for aDNA analysis comprised 116 ancient individuals from the Lower Rhine-Meuse area for aDNA analysis (Supplementary Table 2), which included five previously reported individuals^1,6,31^ for whom we generated additional data. We also selected 10 previously reported^6,36^ individuals from relevant contexts outside the Lower Rhine-Meuse area and generated additional data (Supplementary Table 2–4). We performed laboratory work in dedicated clean rooms. We removed the outer layer of teeth and long bones and collected powder from beneath the cleaned surface. This process minimized the risk of exogenous DNA contamination, with low-speed drilling used to prevent heat-induced DNA damage^55^. In the case of temporal bones, we removed cochleae through sandblasting^56^ and then milled them. We incubated the resulting powder in lysis buffer and cleaned and concentrated the DNA from one-fifth of the lysate. We did this either manually or using an automated protocol with silica magnetic beads^57^ and Dabney Binding Buffer^58,59^ for manual extraction. The samples from Trou Al’Wesse, Abri Sandron, and Grotte du Mont Falise were prepared and extracted following the method outlined in Dulias *et al*.^60^.

We built 254 libraries (Supplementary Table 2) using two different protocols. Double-stranded barcoded libraries were prepared with truncated adapters from the extract and subjected to partial (“half”) uracil–DNA–glycosylase (UDG) treatment before blunt-end repair to significantly reduce the characteristic damage pattern of aDNA^61,62^. Single-stranded libraries were prepared using automated protocols following Gansauge *et al*. ^63^. A fraction was subjected to USER treatment^63^.

DNA libraries were enriched for human DNA using probes that target 1,233,013 (‘1240k capture’^64^) or 1,352,535 (‘Twist’ BioSciences^65^) nuclear SNPs and the mitochondrial genome. We performed two rounds of capture for the ‘1240k’ reagent and one for the ‘Twist’ BioSciences reagent. Captured libraries were sequenced on an Illumina HiSeq X10 instrument with 2x101 cycles and 2x7 cycles to read out the two indices^66^ or on an Illumina NextSeq 500 instrument with 2×76 cycles and 2×7 cycles to read out the two indices.

For three previously reported individuals (I4075, I0103, I0104), we generated shotgun data (Supplementary Table 2) from the same libraries that were 1240k captured in the original publications^6,36^.

### Bioinformatics: Demultiplexing, adapter removal, mapping, PCR duplicate removal:

Reads for each sample were extracted from the raw sequencing data based on sample-specific indices introduced during wet-lab processing, permitting up to one mismatch. Adapters were removed and paired-end sequences were merged into single-ended sequences with a required 15-base-pair overlap (allowing one mismatch with high quality bases or three mismatches with low quality bases), using a modified version of SeqPrep v.1.1 (https://github.com/jstjohn/SeqPrep). This process was applied by selecting the highest-quality base in the overlapping region. Reads that could not be merged were discarded before aligning to the human reference genome (hg19) and the RSRS version of the mitochondrial genome using the ‘samse’ command in bwa (version 0.7.15)^67^. We removed duplicates based on the alignment coordinates and orientation of the aligned reads. Aligned sequences from different libraries of the same sample were merged accordingly into a single bam file. The computational pipelines are available on GitHub (https://github.com/dReichLab/ADNA-Tools, https://github.com/dReichLab/adna-workflow).

### Evaluation of authenticity:

We established aDNA authenticity using several criteria. Libraries with a deamination rate below 3% at the terminal nucleotide were excluded from further analysis. We computed the ratio of Y-chromosome to X- and Y-chromosome reads. Libraries with ratios above 0.03 and below 0.32 were excluded from further analysis. We estimated mismatch rates to the consensus mitochondrial sequence using contamMix-1.0.1051^68^, and X-chromosome contamination estimates using ANGSD^69^ in males with sufficient coverage. Libraries with evidence of contamination were excluded from further analysis. Finally, individuals without a minimum of 20,000 targeted 1240k SNPs with at least one overlapping sequence were discarded from population genetic analysis. After applying these filters, 122 libraries from 59 individuals remained (Supplementary Table 2), and we merged data from the libraries to increase sequencing coverage. Of these 59 individuals, 44 were newly reported individuals from the Lower Rhine-Meuse area, five previously reported from the same area and 10 previously reported from other areas.

### Analysis datasets:

In addition to the 49 individuals with newly generated data from the Lower Rhine-Meuse area, we also included data from 63 previously published individuals^1,6,28–31^ from the region, for a total of 112 individuals from the Lower Rhine-Meuse region and adjacent areas between 8500-1700 BCE (Figure 1; Supplementary Table 1). The time-transect dataset includes three new Mesolithic individuals from Belgium, two from the Netherlands and one from northwest Germany, as well as published data from three individuals from now submerged areas of Doggerland^28^; ten Early-Middle Neolithic individuals from semi-agrarian Swifterbant contexts (4600-4000 BCE) (Netherlands) - the first data from this unique culture; the first three Middle Neolithic individuals from Late Swifterbant or Hazendonk archaeological contexts (Netherlands); three likely Middle Neolithic individuals from Tiel (Netherlands) and one Middle Neolithic individual from Baltrum island (northwest Germany); four published Middle Neolithic individuals from Blätterhöhle cave (3500-3000 BCE) ^1^ (northwest Germany); 40 published Middle Neolithic individuals from a Wartberg context (3500-2800 BCE)^29^ (Niedertiefenbach, northwest Germany); 18 Late Neolithic individuals buried in caves from the Ardennes region (3300-2500 BCE) (Belgium); three Late Neolithic individuals from Vlaardingen/CW contexts, including the first data from this culture from the Lower Rhine-Meuse area (3000-2500 BCE) (Netherlands); 13 Late Neolithic individuals from BB contexts (2500-2000 BCE) (Netherlands); and six individuals from an Early Bronze Age context (2000-1700 BCE) (Netherlands).

To aid the analysis of the Lower Rhine-Meuse area individuals, the analysis dataset was further complemented by previously published data from ancient individuals from other regions (Extended Data Figure 1, Supplementary Tables 3–4). For genome-wide analyses, we assembled two datasets. The HO dataset included the ancient individuals, and 1,036 present-day West Eurasian individuals genotyped on the Affymetrix Human Origins Array^4,70,71^. We kept 591,642 SNPs shared between the 1240k capture and the Human Origins Array. The HOIll dataset included only the ancient individuals and 1,233,013 SNPs in common between 1240k and Twist reagents. In both datasets, we randomly sampled one allele at each SNP position for each individual, discarding the first and the last two nucleotides of each sequence.

### Haplogroup assignment of uniparentally inherited markers:

We created consensus mitochondrial haplotypes with samtools and bcftools. We restricted to sequences with a mapping quality of more than 30 and a base quality of more than 30. We then called haplogroups with Haplogrep3^72^ (Supplementary Table 1). We called Y-chromosome haplogroups (Supplementary Table 1) following the methodology in Lazaridis *et al*.^73^, based on the YFull v.8.09 phylogeny (https://www.yfull.com/). Haplogroups found in Neolithic individuals were classified as either “hunter-gatherer-related” if they were already present among Mesolithic hunter-gatherers (mitochondrial haplogroups U5, U4’9, U2, U* and K1e; Y-chromosome haplogroups I2a, C1a, and R1b-V88), or “Neolithic related” if they were most likely introduced by incoming Early European farmer populations (all mitochondrial and Y-chromosome haplogroups except those mentioned above) (Supplementary Table 13). With this approach, we understand that we might be underestimating the number of lineages contributed by Neolithic farmers, both in the mtDNA and Y-chromosome, as some lineages considered as Mesolithic hunter-gatherer-related, based on their presence during the Mesolithic period, might have been incorporated by farmer populations during their path from Anatolia to the Lower Rhine-Meuse area.

### Molecular sex determination:

Genetic sex was determined by calculating the ratio of reads mapped to Y-chromosome SNP positions to the total reads mapped to sex-chromosome SNP positions. Individuals with a ratio <0.03 were classified as female, while those with a ratio >0.32 were classified as male (Supplementary Table 1).

### Biological relatedness:

To estimate close biological relatedness up to the 3^rd^ degree, mismatch rates were computed between all possible pairs of Lower Rhine-Meuse area individuals, randomly sampling one read for each individual at each of the 1.15 million autosomal SNPs. Mismatch rates were converted to relatedness coefficients following Fowler *et al*.^74^, using three different baseline mismatch rate values to account for the different ancestral backgrounds found in the dataset. If both individuals in the pair had fully Mesolithic hunter-gatherer ancestry, we use a baseline mismatch rate of 0.225. If at least one individual has EEF ancestry and both lack steppe-associated ancestry, we use a baseline mismatch rate of 0.252. If at least one individual has steppe-associated ancestry, we use a baseline mismatch rate of 0.258. Close kinship relationships are annotated in Supplementary Table 1.

### IBD:

We called IBD segments between the Lower Rhine-Meuse delta individuals with high quality data (n=59) and all the previously published ancient individuals from Eurasia with high quality data (n=7034). We followed the same procedure described in Ringbauer *et al.*^39^, which involves imputing and phasing the aligned sequenced data with GLIMPSE^75^ using haplotypes in the 1000 Genome Project as the reference panel^76^, and detecting IBD segments with ancIBD (https://github.com/hringbauer/ancIBD). Pairs of individuals connected by IBD are displayed in Supplementary Table 14. Three individuals from the site of Tiel Medel-de Roeskamp have uncertain chronology. The site has a Middle Neolithic Swifterbant occupation but also a Bronze Age occupation phase, and the sampled individuals were not amenable to radiocarbon dating. We therefore used their IBD connections to estimate an approximate chronology. Their largest IBD sharing is with I33738, a Middle Neolithic Swifterbant individual from Zoelen de Beldert (Netherlands) dated to 4200-3800 BCE, with whom one of Tiel Medel-de Roeskampindividuals shares four IBD segments longer than 8 cM (the longest being 20.5 cM), for a total share of 53 cM (Supplementary Table 14). The second and third largest IBD sharing are with a Neolithic individual from Hazleton North (England)^74^ who lived 3750-3500 BCE (3 IBD segments for a total of 39 cM) and with a Neolithic individual from Gurgy les Noisats^77^ dated to 4836-4606 cal BCE (5855±40 BP, Lyon-4446, SacA-8629) (2 IBD segments for a total of 33 cM). Based on these IBD results, a Bronze Age chronological attribution is extremely implausible for these individuals, and we thus approximate their date to the range 3800-3600 BCE, hence within the Middle Neolithic. This chronology fits well with their lack of steppe-associated ancestry in the autosomal genome, which already suggested a pre-2500 BCE date.

### Runs of homozygosity:

We called runs of homozygosity using hapROH^78^ for individuals with more than 300,000 available SNPs from the 1240k capture panel (Supplementary Table 1).

### Principal component analysis:

We ran Principal Component Analysis on the HO dataset using the smartpca software from the EIGENSOFT package^79^. We computed PCs on 1036 present-day West Eurasians genotyped on the Affymetrix Human Origins Array. Ancient individuals were projected onto those PCs using lsqproject:YES and shrinkmode:YES.

### qpAdm:

We used *qpAdm*^5^ to estimate ancestry proportions. We set the parameters allsnps: YES and inbreed: YES to account for the use of pseudo-haploid data (Supplementary SI 4). We ran each *qpAdm* model with four different setups:

1. Using all 1240k autosomal SNPs.
2. Using 469k autosomal SNPs reported to greatly reduced the bias when co-analysing 1240k data with Twist and shotgun data^65^.
3. Using 711k autosomal SNPs with reduced bias identified using a novel approach (https://github.com/rmnfournier/compatibility-panel) to filter out biased SNPs^80^.
4. Using all 1240k autosomal SNPs but featuring only Twist or shotgun data in the target, source and outgroup populations (Supplementary Table 4).

Throughout this manuscript, we use terms such as “WHG”, “EHG”, “EEF” as genetic shorthand for ancestry components maximized in western European hunter-gatherers, eastern European hunter-gatherers, and Neolithic farmers of Anatolian origin, respectively. These labels refer solely to patterns of shared genetic ancestry and do not imply any specific subsistence strategy, cultural affiliation, or social identity for individuals in whom these components are detected.

### Admixture date estimates:

We applied DATES^81^ to estimate admixture dates leveraging linkage disequilibrium decay (Supplementary Table 15; Extended Data Figure 4). We used the same ancestral population as in *qpADM* modelling. For Neolithic groups/individuals without steppe ancestry, we used Balkan_N+WHG to study EEF-WHG admixture. To study Corded Ware-European Neolithic admixture, we used MN_Wartberg+Germany_CordedWare for Lower Rhine-Meuse BB, Lower Rhine-Meuse EBA and England BB, MLN_Belgium+Germany_CordedWare for Vlaardingen/CordedWare and GlobularAmphora_LN+Germany_CordedWare for SEGermany_BB. To convert the number of generations since admixture given by DATES into years, we assumed 28 years per generation^82^. We used the following parameters: binsize: 0.001; seed: 77; maxdis: 1; qbin: 10; jackknife: YES; afffit: YES; runfit: YES; lovalfit: 0.45 and minparentcount: 1. We considered the earliest, the most recent and the mean possible chronological dates for each group when estimating admixture dates (Supplementary Table 15). The estimates obtained for Vlaardingen/CordedWare group were considered as invalid, since the standard error was higher than the estimated number of generations.

### Radiocarbon chronology:

The chronology for each site is described in Supplementary Information 2. All dates have been obtained from the original excavation reports, follow-up publications or from direct consultation with the excavators. These have been supplemented by 14 new measurements. Full lab codes, pretreatment steps, and quantitative quality indicators are reported in Supplementary Table 16. All reported ages were calibrated with the IntCal20 calibration curve using OxCal 4.4.4^83^.

Several of the dates are potentially susceptible to marine, or freshwater reservoir effect (FRE). However, owing to the dynamic hydrological regime of the Rhine Meuse lowlands, it is often impossible to accurately estimate the source of the FRE, and there is currently no consensus on the correction factor to use for the region^16,84–86^. Therefore, all reported radiocarbon dates have not included an FRE correction factor and we address a potential bias qualitatively in the site descriptions (Supplementary SI 2).

## Extended Data

**Extended Data Figure 2.**
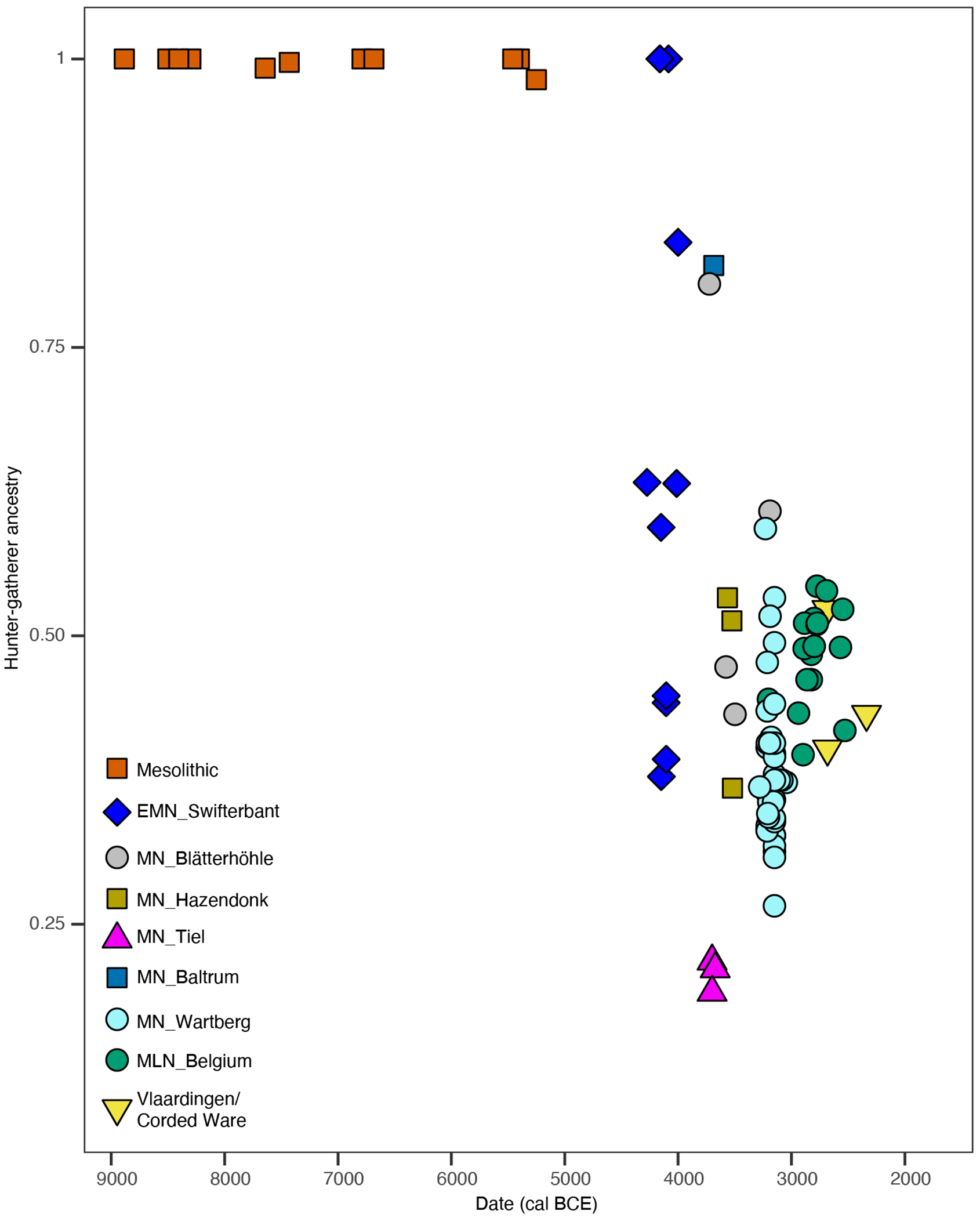
Hunter-gatherer ancestry proportions across time in the Lower Rhine-Meuse area. Ancestry proportions were estimated using *qpAdm* (Supplementary Table 6).

**Extended Data Figure 3.**
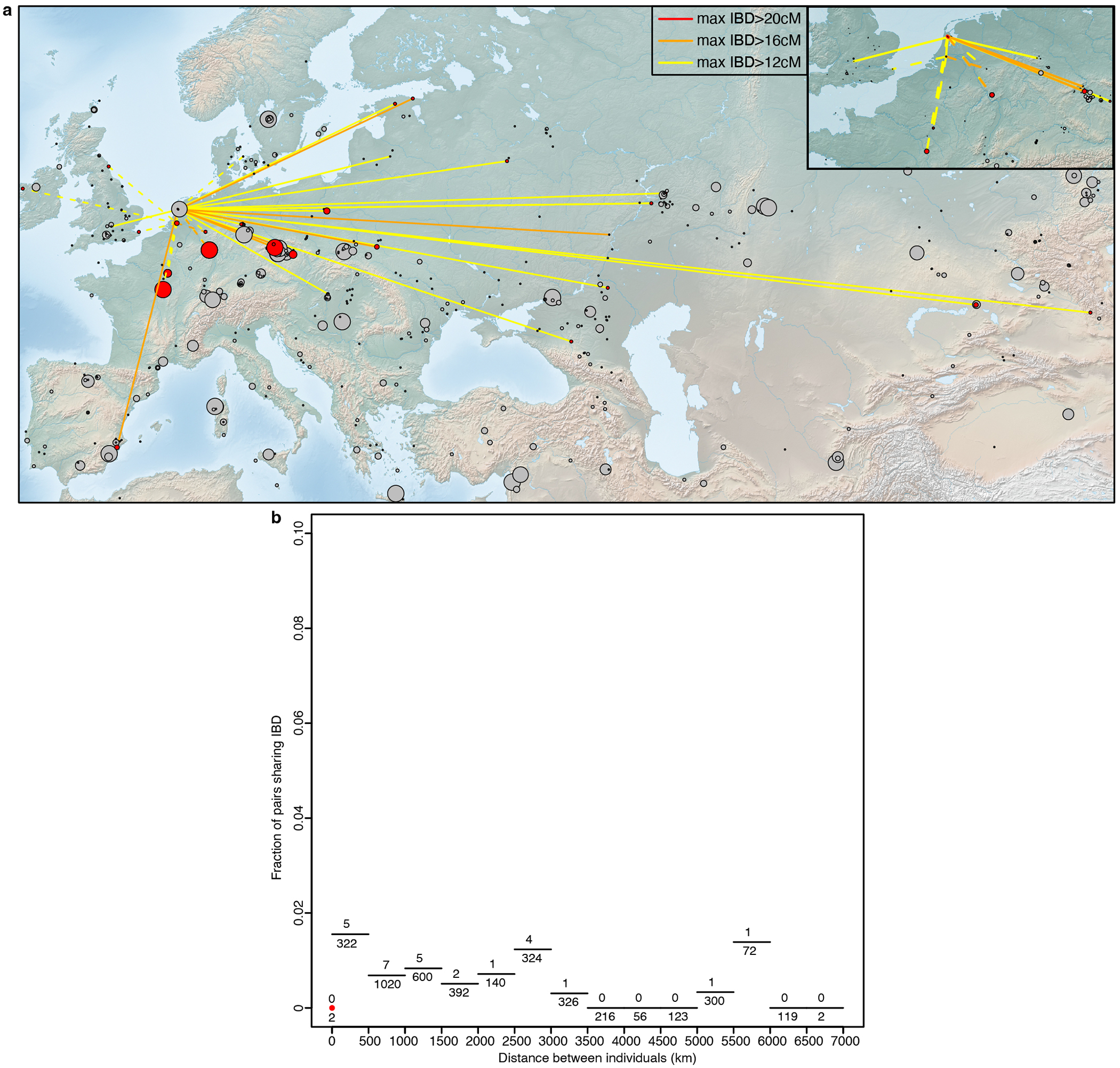
Genetic connections of Lower Rhine-Meuse CW/Vlaardingen individuals (n=3). **a)** IBD sharing of Lower Rhine-Meuse CW/Vlaardingen individuals. Sites are represented by circles with size proportional to the number of individuals amenable to IBD calling. Grey circles indicate archaeological sites between 3000-2000 BCE with no IBD connections to CW/Vlaardingen individuals. The map was drawn using public-domain Natural Earth data with the rnaturalearth package in R^87^. **b)** Decay of IBD sharing with geographic distance for Lower Rhine-Meuse CW/Vlaardingen individuals. Pairs were considered to share IBD if they share at least one segment of >12cM. Dotted lines represent IBD connections involving at least one individual without steppe-related ancestry.

**Extended Data Figure 4.**
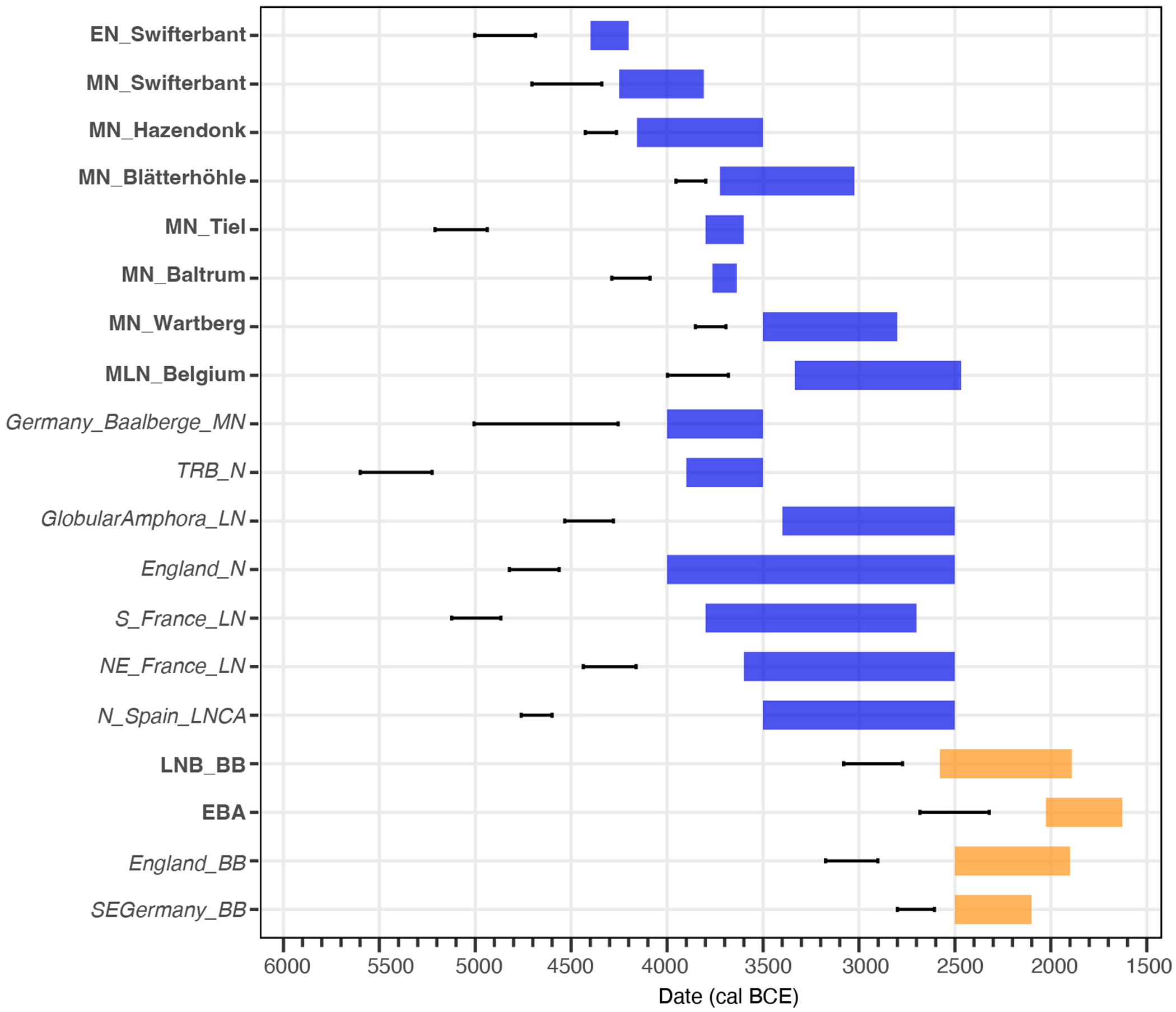
Admixture time estimates using DATES^81^. Boxes represent the chronological range for each population. Confidence intervals represent admixture date ranges, using 28 years per generation and the average date of the chronological range. We tested Balkan_N+WHG admixture for groups in blue, and MN_Wartberg+Germany_CordedWare admixture for groups in orange. In bold, groups from the Lower Rhine-Meuse region.

## Supplementary Material

LowerRhine_SupplementaryInformation

LowerRhineMeuse_SupplementaryTables

## Figures and Tables

**Table 1.** Modelling the ancestry of Lower Rhine-Meuse BB-associated individuals and the main group of British BB-associated individuals using *qpAdm* models involving a CW group from present-day Germany and different Neolithic populations from within and outside the Lower Rhine-Meuse area. See Supplementary Table 10 for further details.

	
	Lower Rhine-Meuse BB	British BB
P-value for models with northern Iberian farmers	0.00079	0.0072
P-value for models with northeast French farmers	0.00015	0.00077
P-value for models with German Baalberge farmers	0.000015	0.000085
P-value for models with Globular Amphora farmers	0.0012	0.0071
P-value for models with TRB farmers	0.00000018	0.00000042
P-value for models with MLN Belgium (Lower Rhine-Meuse)	0.0605	0.0057
P-value for models with MN Wartberg (Lower Rhine-Meuse)	0.65	0.95
Proportion of ancestry from LN Wartberg (Lower Rhine-Meuse)	17.9%	18.9%

Minimum proportion of ancestry from MLN Belgium allowing 3-way *qpAdm* models with CW and other farmer sources outside the Lower Rhine Meuse area	10.9%	9.3%

## Data Availability

Genotype data for individuals included in this study can be obtained from the Harvard Dataverse repository through the following link (https://reich.hms.harvard.edu/datasets). The DNA sequences reported in this paper are deposited in the European Nucleotide Archive under accession number PRJEB105335. Other newly reported data, such as radiocarbon dates and archaeological context information, are included in the manuscript and supplementary files.
