# Supplementary material for: Lasting Lower Rhine-Meuse forager ancestry shaped Bell Beaker expansion
Source: Nature. Author manuscript; Available in PMC 2026 Mar 12. (PMC12978843; doi:10.1038/s41586-026-10111-8)
Supplement: LowerRhine_SupplementaryInformation [file NIHMS2148481-supplement-LowerRhine_SupplementaryInformation.docx]

**Supplementary Information**

**Lasting Lower Rhine-Meuse forager ancestry shaped Bell Beaker expansion**

**Table of contents**

SI 1. Archaeological overview of the Lower Rhine-Meuse region 8500-1700 BCE

SI 2. Archaeological context information about the newly reported and published individuals from the Lower Rhine-Meuse area

SI 3 Analytical details Sr-O-C isotope analysis

SI 4. *qpAdm* modeling of ancestry proportions

# SI 1. Archaeological overview of the Lower Rhine-Meuse region 8500-1700 BCE

A mosaic of ‘cultures’: an archaeological survey 8500-1700 BCE of the Low Countries

Harry Fokkens, Quentin Bourgeois, Eveline Altena, Luc Amkreutz

## 1.1 Introduction

In this overview we sketch the broad cultural developments in the Lower Rhine-Meuse delta in a temporal framework. The goal of this supplement is to provide an archaeological background for the interpretation of the DNA results. Since the Neolithic is characterized by regionally different manifestations of similar archaeological cultures, we discuss how the data from the ‘Low Countries’ fit within this complex mosaic.


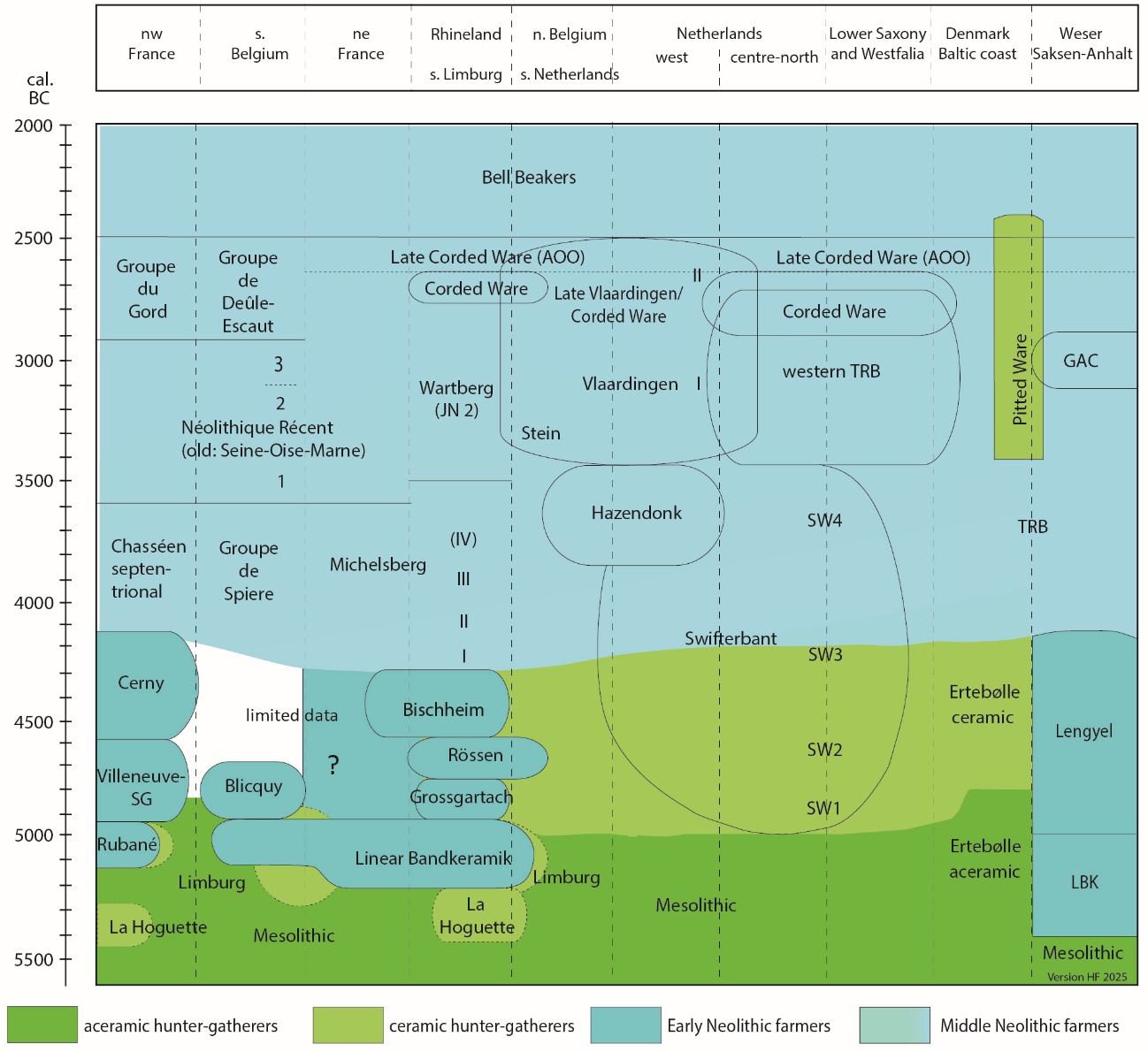


### Figure SI1.1 Schematic overview of archaeological formations in the Early and Late Neolithic in northwest Europe (adapted and updated from Louwe Kooijmans 2006)^1^.

Figure SI1.1 presents the mosaic of cultures that archaeologists recognize against the background of their economic bases and the concept of Neolithization, emphasizing that this was a fluid process in most areas, and not at all a synchronous one. It tries to visualize transitions from hunter-gatherer economies to farming economies in different regions. Early Neolithic farmers are separated from Middle Neolithic farmers in this model in order to indicate the difference between Early Linearbandkeramik (LBK) immigrant farmers and their descendants and a much more diffuse trajectory of adopting farming elements by ‘indigenous’ hunter-gatherer communities. This does not imply that Middle Neolithic farmers ‘slowly’ adopted farming, but that their economy was regionally specific, often relying to some extent on hunting, gathering and fishing. We argue that in wetlands and in river valleys of the Rhine and Meuse the nature of their settlement environment contributed to this different adoption of ‘the’ Neolithic than on the more eastern sandy uplands and southern loess zones.

All radiocarbon dates reported in this document have been recalibrated using Oxcal 4.4 at 95.4% confidence level with the IntCal20 calibration curve^2^. For a more detailed discussion on each individual site we refer to SI2.

## 1.2 Mesolithic (8500-5000 BCE)

### Table SI1.1 Summary of samples used for analysis from the period 8500-5000 BCE

| Genetic ID | Archaeological ID | Locality |
| --- | --- | --- |
| AAT001 | AA3 | Abri des Autours (BE) |
| I7015 | BELG_6598 | ClaminForge (BE) |
| MPR001 | MPR-1 | Malonne Petit Ri (BE) |
| I7018 | BELG_7764 | Grotte_Rousseau (BE) |
| DOG002 | U 2014/12.4; A10-007_V003_M006 | Doggerland, West of Brown Bank |
| I7010 | BELG_265 | Grotte de la faille du burin (BE) |
| DOG001 | A10-007_V002_M003 | Doggerland, Eurogeul |
| DOG007 | U 2014/12.3; A10-007_V001_M001 | Doggerland |
| SPI001 | 2212/2:1 | Spiekeroog (GE) |
| I13024  I38450 | V28.578 Trijntje  V24337 | Hardinxveld-Polderweg (NL)  Hardinxveld-Polderweg (NL) |

## 1.3 Summary of the archaeological context for the individuals in Table SI1.1; for the extended version we refer to SI 2

The Belgian samples are all from (open) cave contexts. Sample I7015 is from a small cave site on a tributary of the Sambre (Claminforge), excavated by Michel Toussaint in 1995^3^. Radiocarbon dates on two individuals from this collection of burials place them well into the Mesolithic. Sample I7018 is from a cave site for which we lack reliable archaeological information (Grotte Rousseau). However, five radiocarbon dates are available for this site. The one that we generated directly from the sample we analyzed for DNA is the only one dating to the Mesolithic, and the genetic profile is typical for Mesolithic western European hunter-gatherers.

All the other dates are from the Middle and Late Neolithic and the related individuals have substantial proportions of ancestry from Anatolian Neolithic farmers. Le Grotte du faille du Burin is a small cave in which the remains of at least 12 individuals were found. Four dates are available, all falling in the Mesolithic^4^. It is not clear whether I7010 was one of those, but the genetic profile is typical for Mesolithic western European hunter-gatherers.

###
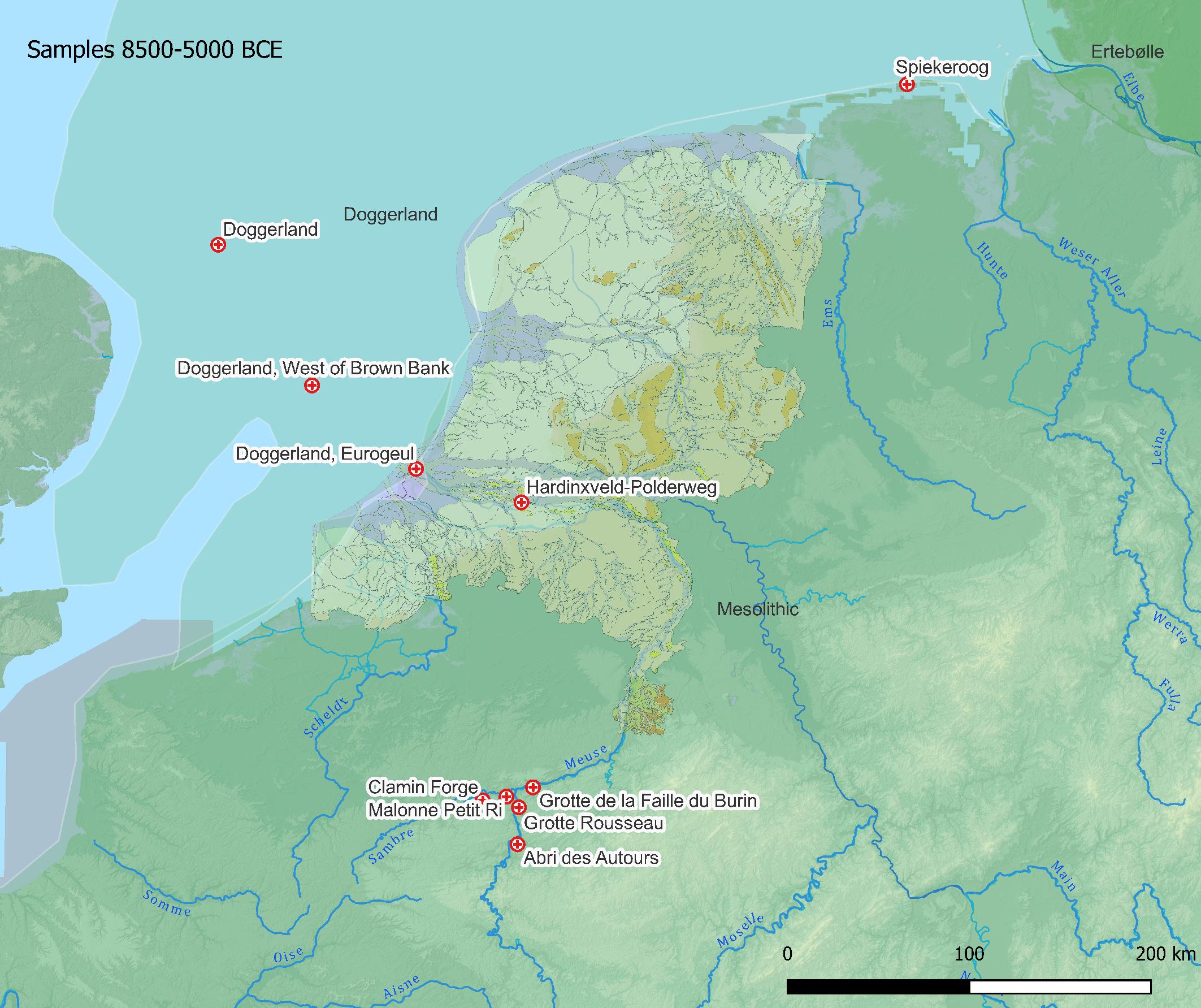
Figure SI1.2 Schematic distribution of cultural spheres and the geographic locations of samples 8500-5000 BCE. The map of the paleogeographic reconstruction of the Netherlands in 9000 BCE^5^ was downloaded from https://nationaalgeoregister.nl/geonetwork/srv/dut/catalog.search#/metadata/c63138f8-775a-4ca2-907c-31f44bd2abf4 in May 2025; the extent of Doggerland is after Coles^6^. The elevation map is from https://www.mapsforeurope.org/datasets/euro-dem (the grey area in the English Channel is uncharted).

The samples from Doggerland, Abri des Autours and Malonne Petit Ri have been published by Posth *et al.* 2023^4^. The Abri des Autours is a cave site on the right bank of the Meuse near the town of Dinant. It contains burial structures from the Mesolithic and the Middle Neolithic ^4,5^. The Grotte du Petit Ri is located in Malonne, near Namur (Belgium). The remains of several individuals, lithics and faunal remains were found ^5,6^. The Doggerland individuals are from different locations in the North Sea, mostly discovered by fishermen or in sand supplies from the North Sea ^8,9^. The sample from Spiekeroog is a beach find from the northern coast of the island. The Hardinxveld-Polderweg samples I13024 and I38450 are from a well-documented grave and from a stray cranium, respectively, from a Mesolithic context on a (now) submerged river dune ^10,11^.

## 1.4 Cultural dynamics in the Mesolithic 8500-5000 BCE

Before early farmers of the LBK reached northwestern Europe, the area —and especially the river deltas and seaside locations— was occupied by Late Mesolithic hunter-gatherer communities with slightly different regional traditions. Especially the vast area of North Sea Doggerland is thought to have been densely occupied^6,12,13^. The Rhine-Meuse delta, bounded in the west by the Scheldt and in the north the Vecht, once formed the eastern fringe of Doggerland. During the Holocene Doggerland gradually drowned due to rising sea levels and changed in an ‘archipelago’ in front of the Thames and Rhine-Meuse estuaries, extending far into the present North Sea^14^. After the Storrega landslide tsunami around 6200 BCE, this process of drowning accelerated^12,13^. Around 5500 BCE Doggerland had completely drowned ^14^, and the sea had advanced to about the present-day coastline of the southern North Sea (*Figure SI1. 2*). Its inhabitants likely migrated toward these new coastlines and connected inland deltas.

In Denmark and on the Baltic coast, the Late Mesolithic Ertebølle hunter-gatherer-fishers formed a ‘*coastal adaptation with a focus on marine resources, especially fish*’^15^, in particular cod. In northern and eastern Jutland, the Ertebølle Culture is best known from its (seasonal) shell middens with oyster shells (*kjøkkenmøddinger*):^16^, but many more sites are known without shell middens, both on the coast and inland ^15,17^. In the Dutch coastal areas and deltas the hunter-gatherers of the Swifterbant culture practiced a similar economy until c. 4200 BCE. From that period onwards they also practiced arable farming and husbandry while continuing to exploit freshwater resources and living on raised areas in, or bordering the wetlands ^18,19^.

Hunter-gatherers started to produce Swifterbant pottery from between 5100 and 4800 BCE (Dreshaj *et al.* 2023), at approximately the same time (4800 BCE) as in Belgium ^20^ and Ertebølle contexts ^21^, demonstrating that these hunter-gatherer-fisher communities were in close contact with each other. Farming was not adopted until a few hundred years later^22^.

In some respects, the Mesolithic occupation in Belgium resembles the Dutch situation, with characteristics very similar to the Swifterbant hunter-gatherers along the Scheldt and Meuse River valleys^23^.

## 2 The first farmers (5000-4000 BCE)

### Table SI1.2 Summary of the samples used for analysis from the period 5000-4000 BCE

| Genetic ID | Archaeological ID | Locality |
| --- | --- | --- |
| I12091 | NGKL10-ID1 | Nieuwegein-het Klooster (NL) |
| I12093 | NGKL10-ID5 | Nieuwegein-het Klooster (NL) |
| I12094 | NGKL10-ID6 | Nieuwegein-het Klooster (NL) |
| I17968 | NGKL10-ID4 | Nieuwegein-het Klooster (NL) |
| I33738 | skelet I | Zoelen_de Beldert (NL) |
| I33739 | skelet II | Zoelen_de Beldert (NL) |
| SWA001 | Skelet II | Swifterbant S2 (NL) |
| SWA002 | Skelet III | Swifterbant S2 (NL) |
| SWA004 | Skelet IV | Swifterbant S2 (NL) |
| I38442 | Grave 2, S454 | Angeren-Kampsepad Zuid (NL) |

###
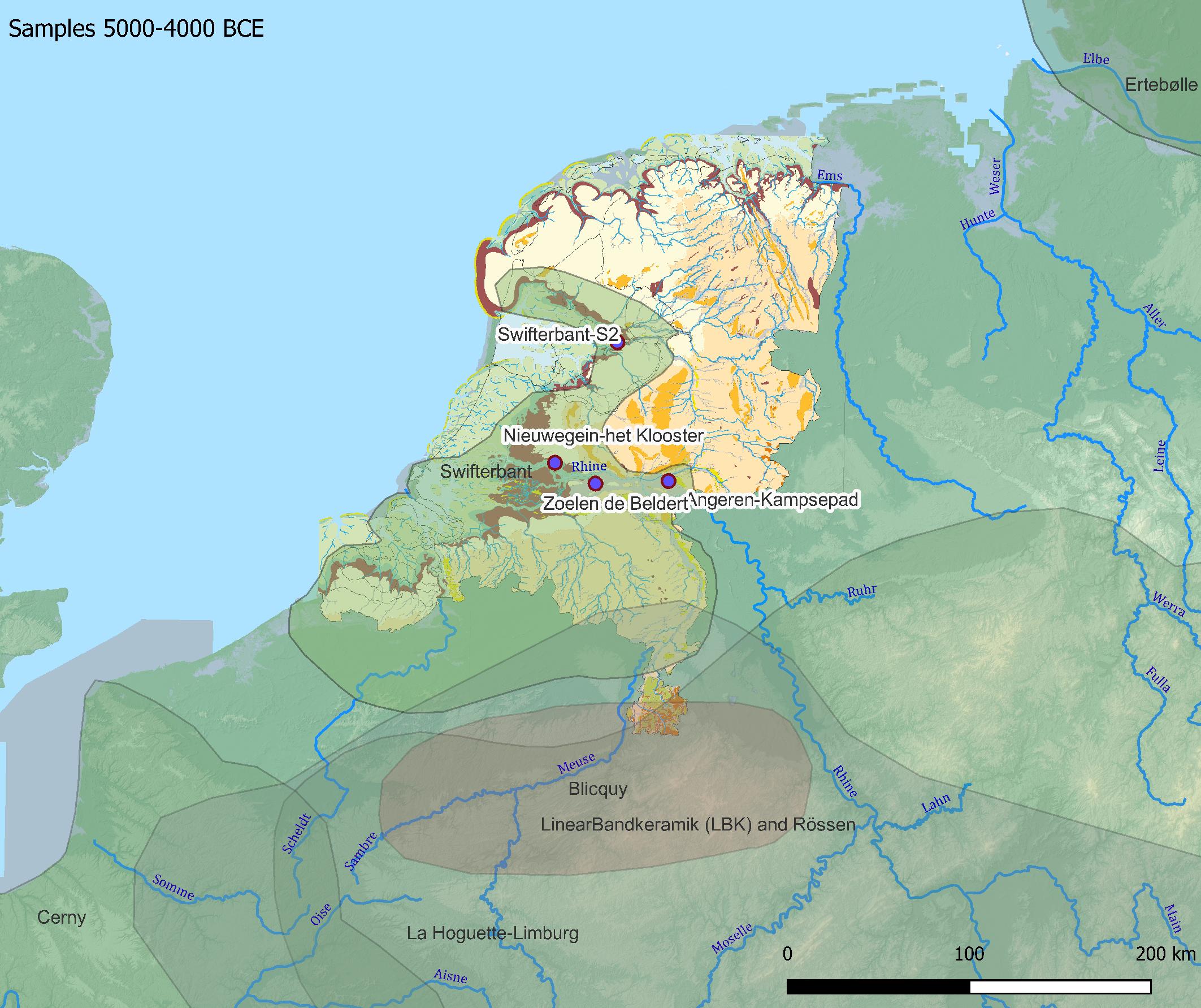
Figure SI1.3 Schematic distribution of cultural spheres and the geographic locations of samples from 5000-4000 BCE. The map of the paleogeographic reconstruction of the Netherlands 5500 BCE^5^ was downloaded from https://nationaalgeoregister.nl/geonetwork/srv/dut/catalog.search#/metadata/c63138f8-775a-4ca2-907c-31f44bd2abf4 in May 2025. The elevation map is from https://www.mapsforeurope.org/datasets/euro-dem (the grey area in the English Channel is uncharted).

## 2.1 Summary of the archaeological context for the individuals in Table SI1.2; for the extended version we refer to SI 2

Three individuals were sampled from one of the eponymous sites at Swifterbant (site S2). These are located on a small river system. Sites S3 and S4 are within meters distance from one another, while S2 is located at some 500 m distance. The site remains make clear that the occupants practiced hunting and animal husbandry, gathering, and cultivation. High resolution Bayesian modelling showed that the site is most likely dated between 4180 and 4030 BCE ^22^, which places it in a later phase of the Swifterbant culture (SW3).

The Nieuwegein-het Klooster site is unique in the Netherlands because it is one of the few Early Swifterbant sites known. At Nieuwegein-het Klooster several graves were found, one of which was of a baby and her mother (I12093 and I12094). Both lacked Early European Farmer (EEF) genetic ancestry, but the other two sampled individuals harbor a mixture of a major ancestry component associated with Mesolithic hunter-gatherers (WHG) and a minor component associated with early farmers (EEF). The same is true for the Late Swifterbant Zoelen-de Beldert individuals, who have a contemporary date.

The individuals analyzed from Zoelen-de Beldert are from a rescue excavation of a pit or grave that contained the remains of three people. Sample I33739 is from skeleton II, a woman lying on the bottom of the pit. On top of that was the secondary deposition of the remains from another woman (sample I33738)^24^. Finally, at the top of the pit a poorly preserved skeleton was found of a child, which did not yield sufficient genetic data.

The individual of the Angeren-Kampsepad site is from one of four graves in settlement near a river channel with stratified deposits containing Hazendonk and Swifterbant type ceramics. The deposits date between 4600 and 3400 BCE. Based on its buried position, the grave is provisionally dated to the Hazendonk period of the Middle Neolithic (3800-3400 BCE; see SI2.14 for more information), but the genetic profile suggests an older (Swifterbant) affiliation.

## 2.2 Cultural dynamics in the Early Neolithic 5000-4000 BCE

The first Linearbandkeramik (LBK) farmers arrived on the loess plateaus of southern Limburg and eastern Belgium west of the Rhine around 5300 BCE^13,25^ (*Figure SI1. 3*). From the archaeological evidence it is clear that, while their economy was fully based on farming, it was strictly bound to the fertile loess soils. The sandy uplands north of this zone were much less fertile, were exhausted more quickly, and were probably less suitable for the cultivation of emmer (*Triticum* *monococcum*) and einkorn (*Triticum* *dicoccum*) wheat, which were staples for LBK farmers^25–28^. From settlement evidence it is clear that LBK farmers also raised sheep, goats, pigs and cattle to supplement their diet^25^. The descendants of these cultures (*Figure SI1. 3*), like the Grossgartach, Rössen, and Bischheim farmers in the east, and the Villeneuve-Saint Germain, Cerny and Groupe de Blicquy farmers in the west, basically grew the same crops and kept similar livestock, although Cerny and Rössen farmers also added bread wheat (*Triticum aestivum*) to their diet^25,29^. The early farming traditions in general kept to the loess belts of eastern and southern Belgium, northern France, the Paris Basin, and the Rhineland^e.g. 30^. There is, however, still a lot of debate about the role of La Hoguette and Limburg pottery. This non-LBK ware occurs also outside the loess areas, and does not seem to have a clear connection with LBK pottery even though it occurs on several LBK sites^31^. Constantin *et al*^32^ therefore think this style is a development from within LBK, while others think it might (also) be related to hunter-gatherer communities^31,33^. From the archaeological evidence it is not clear what happened in Southern Belgium and Northeastern France (*cf. Figure SI1. 1*). In the region situated between the distribution of the Rubané/LBK and Blicquy cultures, the archaeological record is scarce in Southern Belgium and part of Northeastern France, with no Mesolithic and little Neolithic material^34–37^.

Further east, in central Europe, the settlers of the LBK were followed by the early farmers of the Lengyel culture who had their origins probably in Poland^38–40^. Lengyel farmers were still bound to the loess soils and practiced agriculture in a similar way to their LBK predecessors.

While the LBK was thriving across large parts of northern Europe, from France and Belgium in the west to Poland and Ukraine in the east, the river deltas and seashores of the southern North Sea and the Baltic were inhabited by ‘indigenous’ hunter-gatherer-fisher communities. Even though these communities adopted aspects of the Neolithic way of life —first pottery making, and later also elements of farming— it would not be correct to characterize them as (full) farmers just because they practiced farming as well. For this phase of the Neolithic in the wetlands of the Rhine-Meuse delta Louwe Kooijmans^41^ introduced the term “extended broad spectrum economies”. In his later models he adopted the term ‘semi-agrarian’ especially for the communities that in addition to farming, still had hunting and fishing as central parts of their economies^13^. As this appears to have been a constant feature of all communities living in wetlands throughout the Middle Neolithic, even until the Late Neolithic and the Bronze Age^42^.

## 3 Middle Neolithic farmers (4000-3500 BCE)

### Table SI1.3 Summary of samples used for analysis from the period 4000-3500 BCE

| Genetic ID | Archaeological ID | Locality |
| --- | --- | --- |
| BLR001 | Site 2210/5.2-1 | Baltrum (GE) |
| I1565 | Bla8+Bla9+Bla11+Bla24+Bla26(x)+Bla45 (Excavation 2004, 2014 | Blätterhöhle Cave (GE) |
| I1563 | Bla5+Bla7+Bal13+Bla26(o)+Bla30+Bla54 (Excavation 2004, 2014) | Blätterhöhle Cave (GE) |
| I1593 | Bla16+Bla27+Bla59 (Excavation 2004, 2014) | Blätterhöhle Cave (GE) |
| I35542 | grave 4_S1137 | Tiel_Medel (NL) |
| I35543 | grave 1_S1147 | Tiel_Medel (NL) |
| I35544 | grave 3_S2386 | Tiel_Medel (NL) |
| I38121 | 04HP_M120 | Schipluiden-Harnaschpolder (NL) |
| I38447 | Grave 5, Ind. 6; 04HP_M113 | Schipluiden-Harnaschpolder (NL) |
| I38448 | Grave 6, Ind. 7; 04HP_M115 | Schipluiden-Harnaschpolder (NL) |

## 3.1 Summary of the context information for the individuals presented in Table SI1.3; for the extended version we refer to SI 2

The individuals analyzed from Blätterhöhle Cave are from the German Mittelgebirge, at a site with both Mesolithic and Neolithic graves^43,44^. The samples analyzed for DNA came from a scatter of nine bones in the cave all of which yielded DNA and which were treated as independent samples in the study that first reported mitochondrial DNA, but for which genome-wide data showed they belonged to two distinct individuals^45^.

The site of Tiel Medel is situated on a stream ridge or crevasse of the Rhine. It has Swifterbant, but also Hazendonk period occupation and graves. A third phase at the site can be dated to the late 3^rd^-2^nd^ Millennium BCE. The individuals reported here were published as Bronze Age individuals^46^ but the complete lack of Steppe related ancestry in these individuals and analysis suggest that an association with the Middle Neolithic Hazendonk culture is more likely (see SI2. for more information).


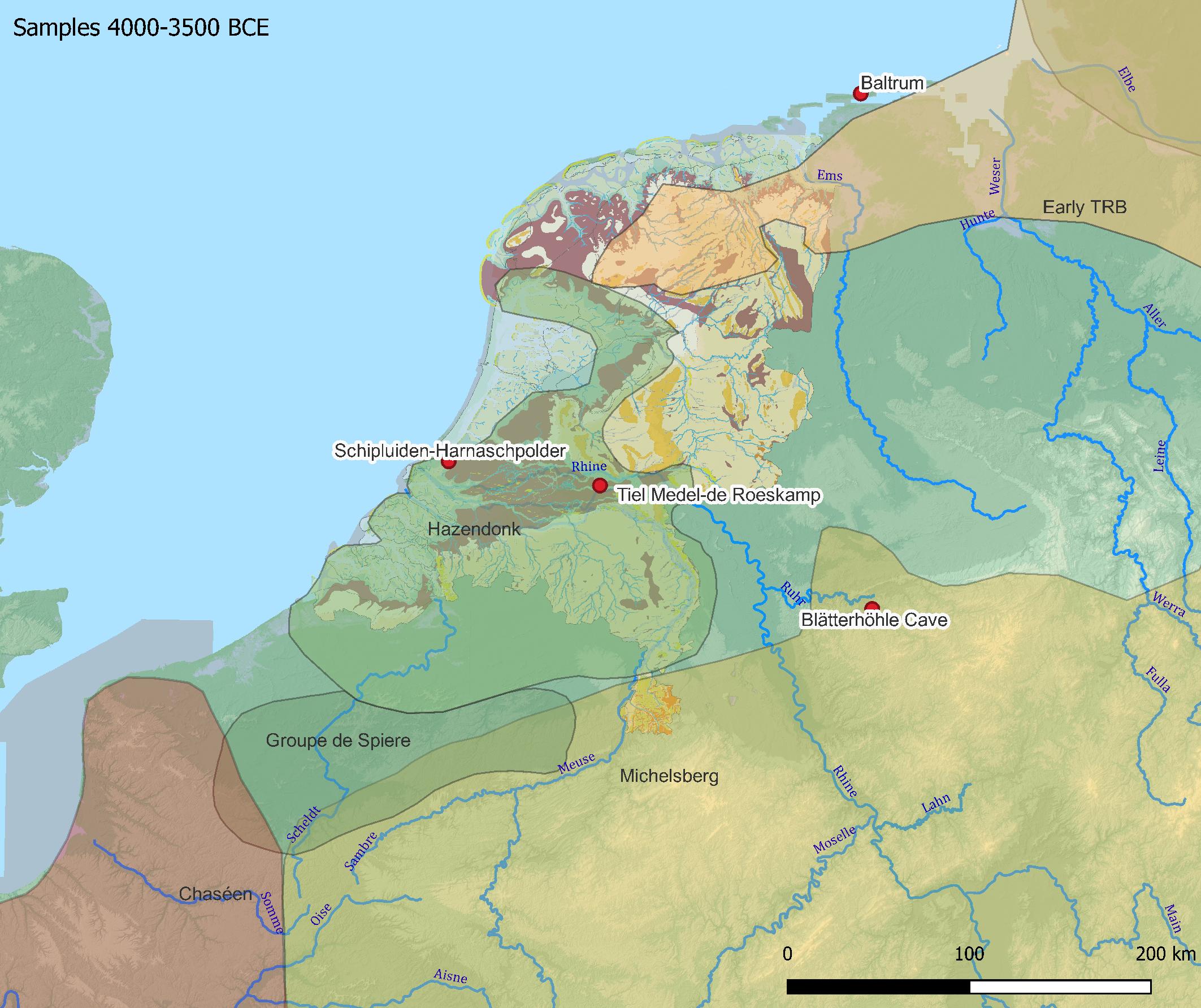
The individuals from Schipluiden are from burials in a Middle Neolithic settlement with huts or houses, situated near the coast on a beach barrier^47^. The habitation on the site must have ended before 3400 BCE ^48,49^.

### Figure SI1.4 Schematic distribution of cultural spheres and the geographic locations of samples from 4000-3500 BCE. The map of the paleogeographic reconstruction of the Netherlands in 3850 BCE^5^ was downloaded from https://nationaalgeoregister.nl/geonetwork/srv/dut/catalog.search#/metadata/c63138f8-775a-4ca2-907c-31f44bd2abf4 in May 2025. The elevation map is from https://www.mapsforeurope.org/datasets/euro-dem (the grey area in the English Channel is uncharted).

## 3.2 Cultural dynamics in the Middle Neolithic 4000-3500 BCE

In most regions the Middle Neolithic was characterized by the first use of flint and stone axes, exploitation of flint mines, and diversification of agricultural practices and crops^25^. The Funnel Beaker Culture (TRB) successors to the Lengyel Culture, for instance, practiced swidden cultivation on the lighter sandy soils north of the loess belt from c. 4000 BCE onwards^39^. The transition from hunting to farming societies in northern Europe took place in a very short period between 4200 BCE and 3800 BCE. The first TRB pottery appears in a Late Ertebølle context around 4100 BCE without replacing other aspects of Ertebølle material. This suggests that Ertebølle hunters-fishers adopted TRB pottery, yet not their farming practices. *Figure SI1.4* shows the distribution of cultural formations that are recognized for this period. The distribution of Early TRB sites is sketchy because only few are known. We assume that most of the forested uplands of NE Netherlands and NW Germany were uninhabited (although perhaps exploited), based on the very limited archaeological indications for this, even though the area has been investigated. Exceptions are early ‘pre-Drouwen’ TRB occupation in lower lying areas (Schokland-P14^50^; Westingermaar^51^).

The Rhine-Meuse delta is dominated by sites of the Late Swifterbant and Hazendonk traditions. Only the later Hazendonk 3 phase is nowadays distinguished as a local variant of the Swifterbant and Michelsberg cultures (*Figure SI1.1*^52,53^). Its distribution is mainly restricted to the river dunes (*donken*) in the central Rhine-Meuse delta. The only individuals sampled that were associated with Hazendonk 3 pottery, are the individuals from the Schipluiden site.

The Blätterhöhle cave is situated within the northern distribution of the Michelsberg Culture. That is contemporary with similar regional cultures such as the Groupe de Spiere in Belgium^54^ and the Chasséen in northern France^55^ (see Bakels^25^ for an extensive overview of economy and settlement). Even though these groups all practiced farming, their economy was not a full farming economy across the region, with the Groupe de Spiere sites being characterized by wetland locations, and by a mix of farming, hunting, fishing, and gathering^56,57^.

## 4 Middle – Late Neolithic farmers (3500-3000 BCE)

### Table SI1.4 Summary of samples used for analysis from the period 3500-3000 BCE

| Genetic ID | Archaeological ID | Locality |
| --- | --- | --- |
| I1594 | Bla28 (Excavation 2004) | Blätterhöhle Cave (BE) |
| KH150613_KH180043 | NT107 | Niedertiefenbach (GE) |
| KH150614_KH150615 | NT142.1 | Niedertiefenbach (GE) |
| KH150618 | NT48 | Niedertiefenbach (GE) |
| KH150619 | NT42 | Niedertiefenbach (GE) |
| KH150620 | NT148 | Niedertiefenbach (GE) |
| KH150621 | NT50 | Niedertiefenbach (GE) |
| KH150622 | NT130 | Niedertiefenbach (GE) |
| KH150623 | NT135 | Niedertiefenbach (GE) |
| KH150625 | NT83 | Niedertiefenbach (GE) |
| KH150626 | NT58 | Niedertiefenbach (GE) |
| KH150627 | KI11 | Niedertiefenbach (GE) |
| KH150628 | NT150.1 | Niedertiefenbach (GE) |
| KH150629 | NT30 | Niedertiefenbach (GE) |
| KH150630 | KI12 | Niedertiefenbach (GE) |
| KH150633 | KI13 | Niedertiefenbach (GE) |
| KH150635 | KI14 | Niedertiefenbach (GE) |
| KH150637 | NT110 | Niedertiefenbach (GE) |
| KH150639 | NT49 | Niedertiefenbach (GE) |
| KH150640 | NT98 | Niedertiefenbach (GE) |
| KH150641 | KI15 | Niedertiefenbach (GE) |
| KH180044 | NT136.1 | Niedertiefenbach (GE) |
| KH180045 | NT146 | Niedertiefenbach (GE) |
| I13627 | 1 / x4; AF004 | Trou Al'Wesse, Modave (BE) |
| I18068 | T26-C | Pommeroeul (BE) |
| I21570 | T26-J | Pommeroeul (BE) |
| I7014 | BELG_273 | Weris_II (BE) |

## 4.1 Summary of the context information for the individuals presented in Table SI1.4; for the extended version we refer to SI 2

The samples cited above are from Germany, from a cave site in the Mittelgebirge (Blätterhöhle GE), a gallery grave near Niedertiefenbach (GE), from an open air site (Pommereuil BE), a cave site (Trou Al’Wesse) and an *allée couverte* (Weris II). The two German sites are associated with the Wartberg Culture, a late variant of the Michelsberg Culture (Immel *et al.* 2021), while the three Belgian sites are probably associated with the Néolithique Récent^58^ (formerly indicated as the Seine-Oise-Marne (SOM) culture), even though only Wéris II is associated with context finds^59^. The individual from Trou Al’Wesse was excavated in a cave (in the Ardennes; Miller *et al.* 2011; 2012), while the sample from Weris II originated from a megalithic grave of a type that had a wide distribution all over western Europe in this period.

The Pommereuil samples come from a composite burial that was discovered in a Gallo-Roman cemetery as the only inhumation grave^60^. Osteo-archaeological and DNA analysis, combined with intensive dating of the separate bones, revealed that it was composed of at least seven individuals, with Late Neolithic dates for the postcranial skeletal parts and a Gallo-Roman date for the cranium^60^.

The Blätterhole, Niedertiefenbach and Pommereuil sites were published earlier, whereas the new samples published here are from Wéris II and Trou Al’Wesse in Belgium. The Niedertiefenbach site stood out because the 42 sampled individuals showed a relatively high (34-58%) proportion of WHG ancestry despite their relatively young date of 3300 and 3200 BCE^61^.

## 4.2 Cultural dynamics at the end of the Middle Neolithic 3500-3000 BCE

The Late Neolithic begins at different moments between 3500 and 3000 BCE in most regions. Several archaeological cultures have been distinguished, mainly on the basis of burial traditions, pottery styles and flint assemblages.

In the Paris Basin, in the north of France, and the adjacent Rhineland, the late 4^th^ and early 3^rd^ millennium are characterized by what used to be indicated as the megalithic “*Seine-Oise-Marne*” Culture (Néolithique Récent)^58^ (*Figure SI1.5*). This places the Néolithique Récent in the same temporal framework as, for instance, the Vlaardingen, Late Michelsberg, Wartberg and Funnel Beaker (TRB) cultures. From a material culture perspective these regional groups or cultures are closely related, for instance the “SOM”, Wartberg and TRB all built megalithic structures of a kind, which were then often reused until the end of the 3^rd^ millennium BCE. Cultural similarities are mirrored in pottery forms (not or sparsely decorated) and technology (even though these are regionally different), and in economy.


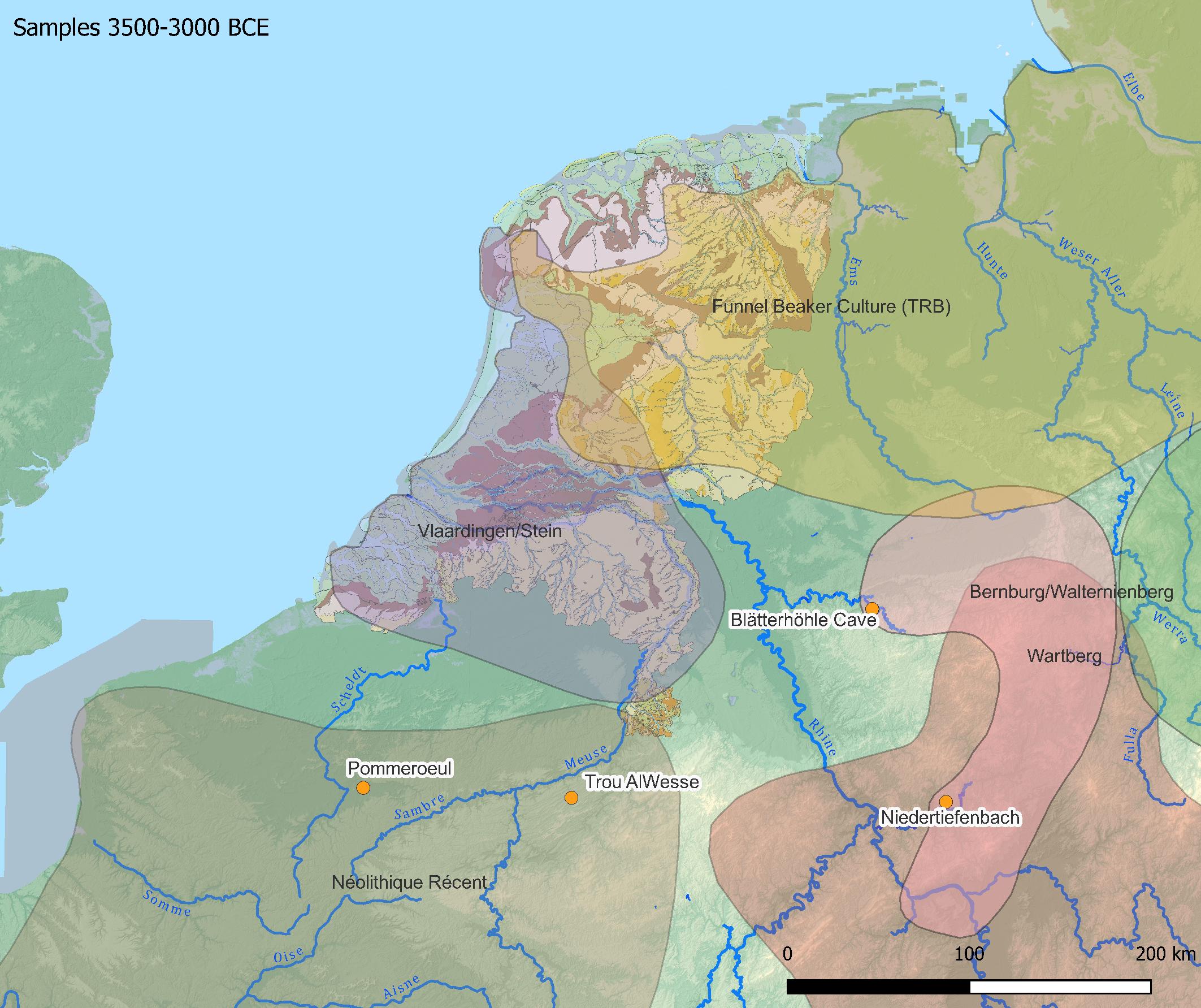


### Figure SI1.5 Schematic distribution of cultural spheres and the geographic locations of newly analyzed and published samples 3500-3000 BCE. The map of the paleogeographic reconstruction of the Netherlands 3850 BCE^5^ was downloaded from https://nationaalgeoregister.nl/geonetwork/srv/dut/catalog.search#/metadata/c63138f8-775a-4ca2-907c-31f44bd2abf4 in May 2025. The elevation map is from https://www.mapsforeurope.org/datasets/euro-dem (the grey area in the English Channel is uncharted).

The only site in the Netherlands that has been associated with the *Néolithique Récent* is a cellar with a stone floor in the Meuse valley near Stein^62–64^. As such it is the most northern expression of a tradition of collective graves in megalithic monuments or in caves and abris that was typical for the *Néolithique Récent.*

Habitation in the deltas of the rivers Scheldt, Meuse, Rhine and Vecht is characterized by Vlaardingen settlements on beach barriers, on Pleistocene river dunes and on levees and crevasse splays but also further inland along the river Meuse and its tributaries (*Figure SI1 5*). Their economy consisted of small-scale farming on these elevated areas, but also hunting, gathering and fishing. In the few cemeteries that have been found near these settlements the dead are buried in a crouched position in ‘flat’ graves without grave gifts apart from amber beads^50,65^.

North and east of the river deltas, on the ice-sculpted and sand-covered landscape of the Veluwe and the northern and eastern Netherlands, the cultural situation appears to have been a little different. These ‘uplands’ were probably heavily forested, and we have little evidence for Neolithic habitation on the plateaus before the TRB tradition started to build their megalithic monuments around 3500 BCE^51^. In northwest Germany and the northeast Netherlands, a separate branch of the TRB developed: the western TRB^66^. There are indications that the first TRB sites developed from 3500 onwards in riverine environments, not unlike those in northwest Germany (e.g. Hüde)^50,51,67^. These sites are all located in the north of the Netherlands and in the Vecht basin. Megalithic monuments were built only in the ice-sculpted areas of the northeastern Netherlands. Only there had the land-ice of the penultimate glaciation brought sufficient boulder material to build these monuments. West of that area, TRB settlement sites have been found only on the western fringes of the sandy ‘uplands’ of the Veluwe, but none in the south of the Netherlands.

Although they were contemporary with the TRB people occupying the Pleistocene ‘uplands’, the overall impression is that the Vlaardingen communities kept to their traditional living areas west of the sandy uplands. We think they were almost inextricably bound to the river deltas and maintained socially defined cultural boundaries with the TRB farmers. It is reasonable to hypothesize that those (immigrant) TRB populations would have relatively high proportions of Anatolian farmer-associated ancestry, similar to the TRB individuals from Denmark that have been genetically analyzed ^68^, but we are not aware of any TRB groups sampled in our research area.

Genetic studies demonstrate that the spread of the TRB culture, where it has been sampled, was associated with a new genetic signature, leading to the conclusion that ‘*…individuals with hunter-gatherer ancestry persisted for decades and perhaps centuries after the arrival of farming groups in Denmark, although they have left only a minor genomic imprint on the population of the subsequent centuries*’^68^. Nevertheless, the hunter-gatherer tradition was not completely ‘eradicated’ by the new wave of farming tradition. Along the Baltic, the southern Swedish and eastern Danish sea coasts, hunter-gatherer-fishers of the Narva, Nema, Zedmar and Pitted Ware traditions kept their own economic strategies and specific material culture until as late as 2200 BCE^69,70^. The Pitted Ware tradition appears in Scandinavia around 3400 BCE, with clear cultural similarity to economies further east. Genetic evidence also shows that Pitted Ware communities had mostly hunter-gatherer ancestry and did not mix with contemporary TRB, GAC and Corded Ware populations^68,71^, even if they exchanged goods and probably practiced some agriculture as well ^72^.

## 5 Late Neolithic A (3000-2500 BCE)

### Table SI1.5 Summary of samples used for analysis from the period 3000-2500 BCE

| Genetic ID | Archaeological ID | Locality |
| --- | --- | --- |
| I12902 | R9560-11_V2764 | Opmeer-Mienakker (NL) |
| I12896 | h 1973/3_18,19 | Molenaarsgraaf-24A (NL) |
| I33741 | Vindplaatsnr. 536_HvH 8077-01 | Sijbekarspel-Op de Veken_de_Veken (NL) |
| I13631 | 213 / 15.019; AF008 | Grotte du Mont Falise (BE) |
| I13638 | 213 / 3252; AF014 | Grotte du Mont Falise (BE) |
| I13649 | 212 / 1x.021; AF026 | Grotte du Mont Falise (BE) |
| I13651 | 212 / 3245; AF028 | Grotte du Mont Falise (BE) |
| I13629 | 213 / 3259; AF006 | Grotte du Mont Falise (BE) |
| I13631 | 213 / 15.019; AF008 | Grotte du Mont Falise (BE) |
| I13633 | 97; AF010 | Abri Sandron (BE) |
| I13635 | 89 / 6763; AF012 | Abri Sandron (BE) |
| I13654 | X1; AF031 | Abri Sandron (BE) |
| I13655 | 88 / 6165; AF032 | Abri Sandron (BE) |
| I13656 | 90; AF033 | Abri Sandron (BE) |
| I13657 | 91; AF034 | Abri Sandron (BE) |
| I13659 | 94; AF036 | Abri Sandron (BE) |
| I13660 | 95; AF037 | Abri Sandron (BE) |
| I7012 | BELG_267 | Grotte Rousseau (BE) |

## 5.1 Summary of the archaeological context of the samples in Table SI1.5; for the extended version we refer to SI 2

Three individuals are from a Dutch context: two from the northwest, and one from the central Netherlands (I12896, I12902 and I33741). IBD analysis indicates that I33741 from Sijbekarspel-Op de Veken de Veken and dated to 2571-2468 cal BCE had a distant kinship (approximately 6^th^-7^th^ degree) with I12902 from Opmeer-Mienakker and dated to 2848-2501 cal BCE (Supplementary Table 14). The individual from Molenaarsgraaf-24A was a chance find and virtually without cultural context^73^. A tooth from the upper jaw of this individual of 30-40 years old (h 1973/3_18,19) yielded sample I12896 and dated directly to 2864-2500 cal BCE.

The Belgian samples cited above are all from the Ardennes, near the river Meuse or its tributaries, from caves that were used for multiple interments over long periods of time. A challenge with these collective burials is that most of them were accessible for long time periods and they may represent multiple burial events, sometimes separated by millennia. However, while this tradition of burying people in caves in this region had already started in the Mesolithic, the vast majority of them date to the turn of the 4^th^ to the 3^rd^ Millennium BCE^74,75^.

Abri Sandron was excavated in the late 19^th^ Century and details are limited. Eight individuals are reported in this study and five have been directly radiocarbon dated to the Late Neolithic. In other publications three additional radiocarbon dates on human remains confirm this general time frame and suggest a short period of use for this cave^73^. While we cannot exclude that some of those without direct radiocarbon dates may date to other time periods, their genetic profile corresponds with this time-frame. One individual sampled from the Grotte Rousseau, was directly radiocarbon dated to the early 3^rd^ Millennium BCE.

###
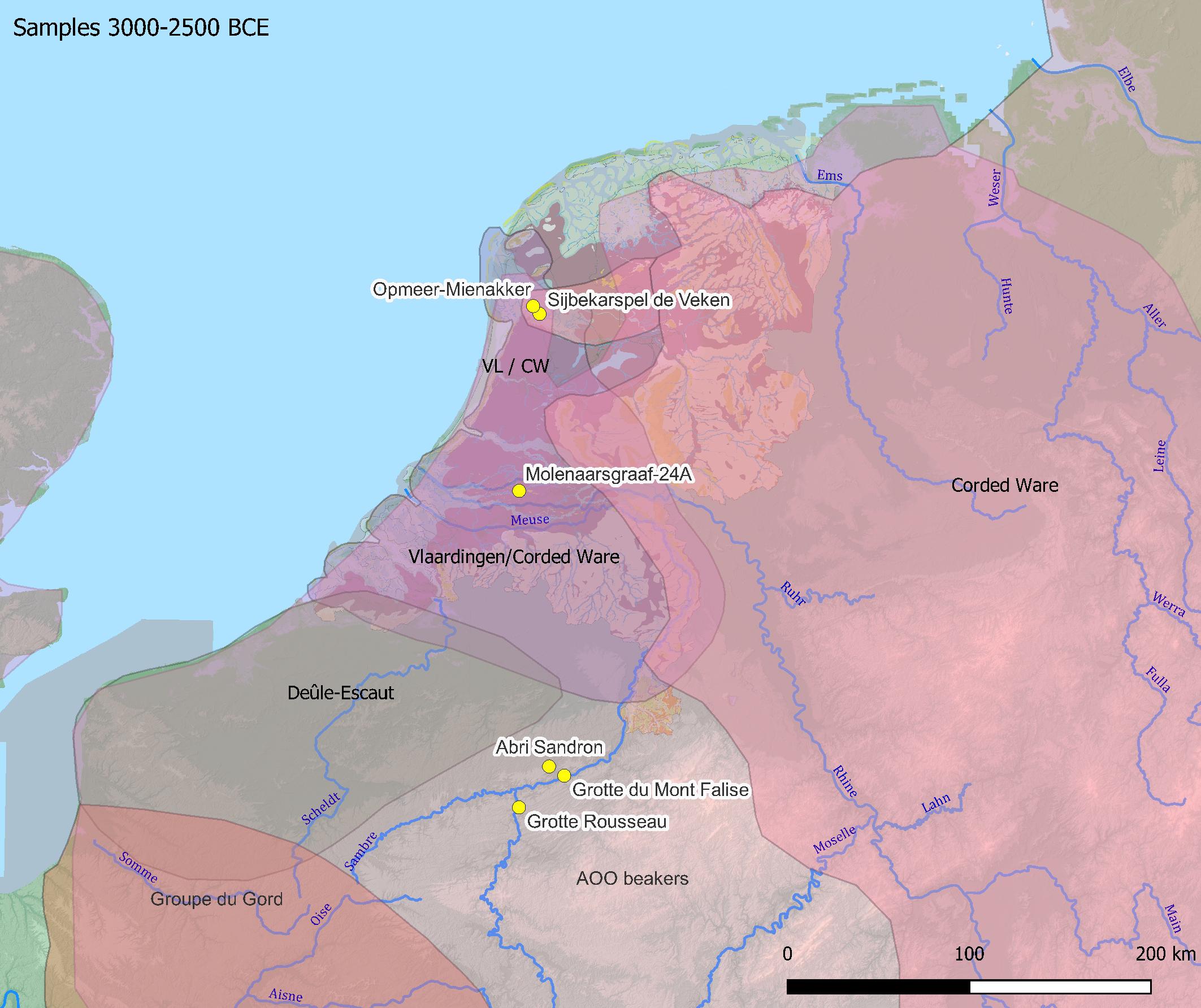
 Figure SI1.6 Schematic distribution of cultural spheres and the geographic locations of samples from 3000-2500 BCE. The map of the paleogeographic reconstruction of the Netherlands 2850 BCE^5^ was downloaded from https://nationaalgeoregister.nl/geonetwork/srv/dut/catalog.search#/metadata/c63138f8-775a-4ca2-907c-31f44bd2abf4 in May 2025. The elevation map is from https://www.mapsforeurope.org/datasets/euro-dem (the grey area in the English Channel is uncharted).

Although other samples from the same cave have been directly dated to the Mesolithic (see I7018), additional radiocarbon dates suggest that most individuals buried in this cave dated to the early part of the 3^rd^ Millennium BCE^73^.

The Grotte du Mont Falise was excavated in the late 19^th^, and early 20^th^ centuries^76^. The context is not very clear but multiple individuals were discovered inside the cave. We included five individuals in this study, with one being directly radiocarbon dated to the first half of the 3^rd^ Millennium BCE. Two additional radiocarbon dates from this site, support this time frame^75^. As with Abri Sandron, we cannot be certain that all sampled individuals date to this period, although it is most likely based on their genetic profile.

## 5.2 Cultural dynamics in the Late Neolithic A: 3000-2500 BCE

In the 3^rd^ millennium several new traditions are recognized. The geographic distribution of these ‘groups’ is not very clear, however (*Figure SI1.6*). Blanchet^77^ and Brunet *et al*^78^ have published generalized site distribution maps that situate the Néolithique Récent in the Paris Basin, reaching up to along the Oise to the River Meuse^77,78^. The distribution of the Groupe du Gord appears to concentrate in the Marne and Oise valleys, though the eponymous site lies near Compiegne, on the Oise river^77–80^. The Groupe du Deûle-Escaut is situated in the area north of the Somme^78^, though most known sites are situated north of the heights of Artois, in the river valleys of the Deûle, Leie and Scheldt (Escaut)^77,78,81,82^. The location of the settlements is similar to those of the Vlaardingen Culture: on elevated areas in the river valleys and deltas. Blanchet^81^, for instance, describes the sites of the Néolithique Récent as situated mostly in river valleys with an economy that depends on farming, but also on a substantial amount of hunting and fishing. Also in Belgium, the Deûle-Escaut group is described as locating its settlements next to rivers and marshes^83–86^ for access to ‘food and different sources from wet environments’^87^.

Both the Groupe du Gord and du Deûle-Escaut are dated to between 2900 and 2550 BCE^88^. They are therefore contemporaneous with Late Vlaardingen, late TRB and the Corded Ware in the Netherlands. From different sources it is clear there were contacts between the people using these regionally slightly different ceramic assemblages. These connections are visible for instance in similar types of ceramic artifacts, notably collared flasks, clay discs, ceramic ‘spoons’ and similarities in the technology and forms of pottery. They are also visible in the distribution of typical ‘Vlaardingen’ flint axes and of flint sources, with large blades of Grand-Pressigny flint as its ‘apotheosis’. Grand-Pressigny flint is one of the typical elements in Deûle-Escaut sites^83^. Its distribution connects the Netherlands with central France through the regions in which the Artenac, Gord and Deûle-Escaut traditions are situated, along major rivers in these areas, including the river Meuse^89–91^.

Taken together, the second half of the 4^th^ and the first half of the 3^rd^ millennium in Northern France, Belgium and the Western Netherlands was characterized by farming communities that supplemented their existence with hunting, fishing and gathering, and situated their settlements accordingly. Even though few reliable structures (houses) are known, these do have similarities. Houses were rectangular, often slightly trapezoidal, substantial, and well built^84,86,92–95^. They represent stable settlement locations, even if these are situated in areas that in modern eyes would not optimally be suited for arable farming. Yet they offered the possibility to optimally use the sources available in these varied environments.

The dead were buried in cemeteries in or near settlements (Vlaardingen)^65^, or in megalithic monuments that were built in the 4^th^ millennium BCE and often reused in the 3^rd^ millennium. In contrast, the megalithic graves of contemporaneous communities contain open and therefore less well dated burial assemblages. This is especially the case with the collective burials in caves and rock shelters. This practice is common along the Meuse river and its tributaries in the south of Belgium and northern France (cf. Toussaint *et al*^75^). The many caves in the region were used for collective burial from the 5^th^ and into the 3^rd^ millennium BC^75,96,97^. The remains are often heavily commingled and there is evidence of post-mortem manipulation of the bones. Most of the burials lack accompanying archaeological material, but those that do, contain artifacts typical for this time period, such as arrowheads or pottery shards of the respective archaeological cultures^75^.

In paragraph 4.2 we already sketched the differences in settlement area between the Vlaardingen culture in the west, almost inextricably bound to the wetlands of the Rhine and Meuse valleys, and the TRB farmers that arrived from the east and settled on the higher ice-sculpted plateaus. Early and Middle Neolithic remains on these ’uplands’ are very scarce, if not completely absent. We assume these regions were heavily forested and used for hunting and gathering, but not (yet) for settlement. Therefore, the TRB farmers are considered to be the first to reclaim these forested areas and convert them into arable land and settled areas. While the oldest TRB sites are known from the wet areas of the Vecht-basin and on the flanks of the ice-sculpted ridges of Wiering and Texel in the north-west, its main distribution, and all of their megalithic monuments, are in the northeast, on the Hondsrug area, and down to the ice-sculpted ridges of the Veluwe in the central Netherlands.

This is also the landscape in which the Corded Ware complex settled. There is increasing evidence now that TRB groups remained in the area until at least 2600 BCE, therefore co-inhabiting the region with Corded Ware people^98,99^ (Kroon 2024; Bourgeois *et al.* in press.). Here we have clear evidence of a transition to the typical Single Grave burial rite that pervades throughout Europe at that time (cf. Furholt 2021a), while at the same time, there is persistence of TRB communities well into the 3^rd^ Millennium BC (Bourgeois *et al.* in press). For instance, cremation burials from the same time horizon as early CW burials have been found associated with TRB pottery^98,99^.

West of these ‘uplands’, in the Rhine-Meuse delta we find only settlements of the Vlaardingen culture, but also with Corded Ware pottery. This is especially the case of the West-Frisian sites in the northwest, but also in other Vlaardingen sites in the western Netherlands. For a long time, these sites have been labeled as Corded Ware sites, but it has become clear that this Corded Ware pottery was not made by immigrant potters, but was made in a Vlaardingen tradition, with Vlaardingen clays and most likely by Vlaardingen potters^98,100–102^. Interestingly Kroon also showed that a substantial number of these pots have inclusions that only could have derived from tertiary deposits like in the Ardennes^101^. Vlaardingen potters therefore most probably had connections to people living higher up in the valley of the Meuse where such tertiary inclusions are probably eroded into natural clays.

In accordance with the cultural situation outlined above, the three individuals (I12896, I12902, I33741) sampled from the western Netherlands are labeled as Vlaardingen/Corded Ware individuals. These three individuals were living on the absolute fringes of the Corded Ware expansion prior to 2600-2500 BC. They were not buried in a classical Corded Ware burial style, but without grave goods and in ‘flat graves’ in settlements with both Corded Ware Beakers and Vlaardingen pottery and artifacts. These Corded Ware Beakers included All Over Ornamented (AOO) Beakers, which have a somewhat wider distribution than other Corded Ware Beakers (*Figure SI1.6*). They are for instance found in Belgium, Northern France and in the British Isles^103–105^, and appear to represent the enhanced mobility that especially marks the following Bell Beaker complex.

Traditionally in the Netherlands, AOO beakers are seen as the latest phase of the CW tradition, for instance because they are still associated with flint knives or ‘daggers’^106,107^. In ^14^C-dates, however, there is a complete overlap with other CW dates, and there is no reason to separate AOO pottery as a distinct phase of Corded Ware^98^.

## 6 Late Neolithic B and Early Bronze Age (2500-1700 BCE)

### Table SI1.6 Summary of samples used for analysis from the period 2500-1700 BCE

| Genetic ID | Archaeological ID | Locality |
| --- | --- | --- |
| I5748 | skeleton 575 (Jan) | Oostwoud-Tuithoorn (NL) |
| I13028 | h 1982/7._4_Skelet I | Ottoland_Kromme_Elleboog (NL) |
| I12900 | h 1982/7._4_Skelet II | Ottoland_Kromme_Elleboog (NL) |
| I13025 | h 1967/1._Skelet I | Molenaarsgraaf (NL) |
| I13026 | h 1967/1._Skelet II | Molenaarsgraaf (NL) |
| I13027 | h 1967/1._Skelet III | Molenaarsgraaf (NL) |
| I4069 | skeleton 229 | Oostwoud-Tuithoorn (NL) |
| I4073 | skeleton 236 | Oostwoud-Tuithoorn (NL) |
| I4074 | skeleton 242/533 | Oostwoud-Tuithoorn (NL) |
| I4075 | skeleton 243 | Oostwoud-Tuithoorn (NL) |
| I5750 | skeleton 230 extra | Oostwoud-Tuithoorn (NL) |
| I20063 | skeleton 232 | Oostwoud-Tuithoorn (NL) |
| I4067 | skeleton 127 | Oostwoud-Tuithoorn (NL) |
| I4070 | skeleton 230 | Oostwoud-Tuithoorn (NL) |
| I4071 | skeleton 231 | Oostwoud-Tuithoorn (NL) |
| I4076  I39210 | skeleton 247  skeleton 233 | Oostwoud-Tuithoorn (NL)  Oostwoud-Tuithoorn (NL) |

## 6.1 Summary of the archaeological context of the samples in Table SI1.6; for the extended version we refer to SI 2

The samples presented in Table SI1.6 are from three sites in the Netherlands. The Molenaarsgraaf site is one of the best documented Bell Beaker / Early Bronze Age settlement sites in the Netherlands^73^. The Molenaarsgraaf individuals were buried in this settlement context. Skeleton 1 (I13025) was buried with a Bell Beaker near his feet but the other two individuals were buried without Beakers, although one of them was accompanied by fishing gear. The settlement situation is typical for a combined farming-hunting-fishing economy. The Ottoland-Kromme Elleboog settlement site is close-by and has the same date^73^. Two individuals were found buried in flat graves in a settlement context. The Oostwoud-Tuithoorn site consists of two burial mounds; one with 12 skeletons dated to the Late Neolithic Bell Beaker Period and the Early Bronze Age, and a second one with two skeletons dated to the Early or Mid-Bronze Age. Both burial mounds were located in extensive arable land with many Late Neolithic and Early Bronze Age Beaker shards^108^.

## 6.2 Cultural dynamics in the Late Neolithic B and Early Bronze Age: 2500-1700 BCE: Bell Beaker and Barbed Wire Beaker


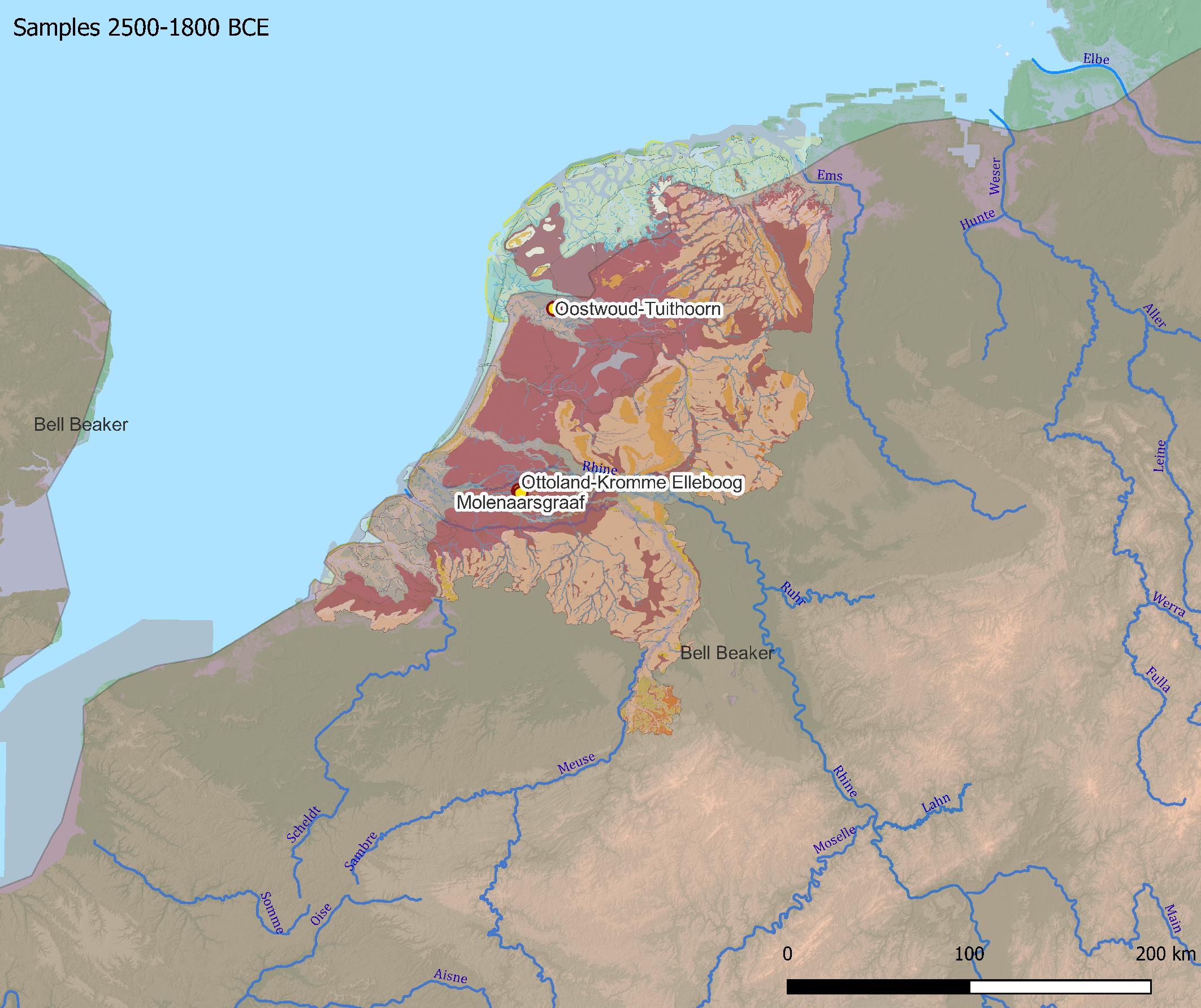
Around 2500 BCE the situation appears to change considerably. The CW and AOO pottery styles are replaced by the Bell Beaker complex almost everywhere in the Netherlands, both in graves and settlements. Also, a large scale genetic turnover becomes visible with the advent of Bell Beaker groups in the region. Olalde *et al.*^109^ already showed the presence of steppe-related ancestry in Oostwoud-Tuithoorn, but samples from previous periods were lacking in this paper. It was therefore not clear if this steppe-related ancestry was already present, or to what extent, in other groups, such as Corded Ware communities. Bell Beaker burial mounds are restricted mostly to the ice-sculpted uplands again, but also extend into the sandy parts of the southern Netherlands and Belgium.

Figure SI1.7 Schematic distribution of cultural spheres and the geographic locations of newly analysed and published samples 2500-1800 BCE. The paleogeographic reconstruction of the Netherlands 2750 BCE is from Vos et al.^5^. The elevation map is from https://www.mapsforeurope.org/datasets/euro-dem (the grey area in the English Channel is uncharted).

Apart from the Oostwoud-Tuithoorn burial mound, no others are known in the western Netherlands. However, settlement sites are discovered everywhere, even in the Rhine-Meuse delta. They are situated in similar locations as their Vlaardingen/Corded Ware predecessors, yet not on the exact same locations. Bell Beaker sites in the west, but also on the eastern uplands, are still situated in river valleys and in general in locations that allowed them to also hunt and fish^42^.

In respect of culture therefore, settlements of the Bell Beaker complex do not offer a major break with those of the Vlaardingen/Corded Ware complex in the Rhine-Meuse delta. How they relate to settlements in the central and eastern Ice-sculpted uplands is not clear, because settlements are virtually lacking in that region. If we find them (for instance at Oldeboorn^42^ and Hattemerbroek^110^), they are situated in locations one would not expect for full farming communities: in low-lying wetlands and in river valleys. They seem to be chosen to optimize the exploitation of the landscape, rather than focusing on farming. It should be noted, however, that present-day construction works are typically not conducted in these types of locations, making it less likely for them to be investigated by development-led archaeology^42^.

Regionalisation of Beaker styles is visible in most countries during the course of the 3rd Millennium BCE ^103–105^, but this is an observation that is almost exclusively connected to pottery style. Bell Beaker settlements remain very scarce (several in the Netherlands, but very few in the adjacent countries: see Besse^111^). Around 2000 BCE a last ‘universal’ phase of the Beaker complex arrived with the Barbed Wire Beakers. These occur everywhere where Bell Beakers were used, from Denmark down to southern France and eastward to Poland. Yet settlements are rare and even burial sites are scarce. In the Netherlands, only the Oostwoud-Tuithoorn burial mounds yielded samples from this period, which ended around 1800-1700 BCE, to be replaced by Middle Bronze Age material culture. In the Low Countries and adjacent areas, this was the period in which the three-aisled longhouse developed as a symbol of social and economic structures that probably were much different from the Late Neolithic Beaker period^112^.

# SI 2. Archaeological context information about the newly reported and published individuals from the Lower Rhine-Meuse area

Edited by Harry Fokkens, Quentin Bourgeois and Eveline Altena

The site summaries provided in this Supplementary Information, are intended solely as a consolidated reference for the samples and associated contextual details. For the radiocarbon dating we rely on previously conducted ^14^C measurements from sites excavated by others. With the exception of 14 new ^14^C measurements (see Supplementary Table 16), all dates have been drawn directly from the original excavation reports, peer‐reviewed articles or obtained via direct consultation with the excavators. Where discussions of marine‐ or freshwater‐reservoir effects (FRE) were critical to chronological interpretation, we have followed the authors’ own assessments and cited the relevant studies. No FRE correction has been applied as currently there is no consensus on a proper adjustment for northwestern Europe^113,114^. The dynamic hydrological regime of the Rhine-Meuse lowlands means that the freshwater effect is often “*unpredictable and not easily explainable”*^115^. If available, we have always tried to anchor the chronology on radiocarbon measurements of other samples such as herbivores, or short-lived plants that are not substantially affected by FRE. All reported calibrated age ranges have been re-calibrated using OxCal 4.4 at 95.4% confidence with the IntCal20 calibration curve^2^.

## *The Netherlands*

## 2.1 Hardinxveld-Polderweg (98POL) (Zuid-Holland, the Netherlands)

**Analyzed individual:**

I13024 (grave 1, V28578)

I38450 (V24337)

**Contact information**: Liesbeth Smits, Leendert Louwe Kooijmans

**Site information and excavation history:** The Mesolithic and Early Neolithic habitation of this site was located on the top of a submerged river dune (*donk* in Dutch) of which 16 x 28 m was excavated in a trench reinforced with steel sheet piling to a depth of about 8.5 m below Dutch Ordinance Datum. All excavated soil was sieved, yielding thousands of organic remains and flint artefacts. The mesolithic part of the site was interpreted as a winter base camp for family groups of Mesolithic hunter-gatherers (Hamburg & Louwe Kooijmans 2001: 31).

Two human burials were found at the site, one of which was severely disturbed, and three dog burials, of which two were disturbed as well. Additionally, several dispersed human bones were found, belonging to at least another ten individuals (Smits & Louwe Kooijmans 2001: 428).

**Summary of the sampled materials:** For this project the undisturbed grave, grave 1 (I13024), and a stray single cranium (I38450, V24337), were sampled. Grave 1 contained the remains of a 40–60-year-old woman (nick-named Trijntje) who had been buried in supine extended position. Her skeleton (find number 28.578), was poorly preserved and therefore limited further inferences about her physical state (Smits & Louwe Kooijmans 2001). The petrous bone yielded sample **I13024.** V24337 was a separate cranium and belonged to a 30-60-year old man **(**Smits & Louwe Kooijmans 2001)**.**

**Dating**:

Grave 1 was directly dated with collagen on the femur. However, the δ^13^C value for grave 1 was high (-22.60 %) and could imply a reservoir effect (Louwe Kooijmans & Mol 2001: 68, table 3.2.). A re-evaluation of the chronology of this site using stratigraphy and radiocarbon measurements on short-lived samples has now proposed that this grave can be dated ca. 5500-5400/5300 BCE (Dreshaj et al. 2023).

**Source of the sample**: Provinciaal archeologisch depot Zuid-Holland; Inge Riemersma, Mark Phlippeau. Samples collected by Eveline Altena.

**Source of the entry**: Liesbeth Smits, Leendert Louwe Kooijmans

**References**:

Dreshaj, M., Dee, M., Brusgaard, N., Raemaekers, D., & Peeters, H. (2023). High-resolution Bayesian chronology of the earliest evidence of domesticated animals in the Dutch wetlands (Hardinxveld-Giessendam archaeological sites). *PLOS ONE, 18(1), E0280619*. https://doi.org/doi.org/10.1371/journal.pone.0280619.

Hamburg, T. D., & Louwe Kooijmans, L. P. (2001). 1. Vooronderzoek en opgraving. In L. P. Louwe Kooijmans (Ed.), *Archeologie in de Betuweroute. Hardinxveld-Giessendam Polderweg: Een Mesolithisch jachtkamp in het rivierengebied (5500-5000 v. Chr.)* (Vol. 83, pp. 13–34). NS Railinfrabeheer.

Louwe Kooijmans, L. P., & Mol, J. (2001). 3. Stratigrafie, chronologie en fasering. In L. P. Louwe Kooijmans (Ed.), *Archeologie in de Betuweroute. Hardinxveld-Giessendam Polderweg: Een Mesolithisch jachtkamp in het rivierengebied (5500-5000 v. Chr.)* (Vol. 83, pp. 55–97). NS Railinfrabeheer.

Smits, L., & Louwe Kooijmans, L. P. (2001). 14. De menselijke skeletresten. In L. P. Louwe Kooijmans (Ed.), *Archeologie in de Betuweroute. Hardinxveld-Giessendam Polderweg: Een Mesolithisch jachtkamp in het rivierengebied (5500-5000 v. Chr.)* (Vol. 83, pp. 419–442). NS Railinfrabeheer.

## 2.2 Zoelen-de Beldert (Gelderland, the Netherlands)

**Analyzed individuals:**

I33738 (skelet I)

I33739 (skelet II)

**Contact information:** Roel Lauwerier

**Site information and excavation history:** North of Tiel, in the villages of Zoelen and Medel, very extensive gravel and sand extractions have taken place from the nineteen eighties onwards. This has led to the discovery of many sites from all kinds of periods, several of which could be excavated. At Zoelen-de Beldert, northwest of Tiel, local archaeologist E. Verhelst discovered a pit with skeletal material that triggered a small rescue excavation in May 1991 (Hogestijn & Lauwerier, 1992, p. 108).

**Summary of the sampled materials:** In the burial pit or grave the remains of three people were buried. Lowermost buried were skeleton II and III, both were buried in an extended prone position on a bed of organic material (leaves or bark). They were the skeletons of a 50–70-year-old female and a child of approximately 7 years old (Hulst et al., 1993; Lauwerier, 1993). The adult skeleton II had been placed on top of the child. This skeleton yielded **sample I33739**. The child's remains did not contain suitable material for aDNA analysis.

The top of the pit contained the secondary deposition of the remains of another 30–60-year-old woman (skeleton I, **sample I33738**). This individual was likely originally buried elsewhere and interred in the new grave only after the body had decayed (Hulst et al., 1993, p. 69). Swifterbant style pottery was found nearby, but not in the grave itself.

**Dating:**

### Table SI2.2 ^14^C dated sampled individuals from Zoelen-de Beldert

| **ID number** | **Sample material** | **Lab code** | ^14^C Age BP ± 1 σ | **95.4% cal BCE** | **δ**^13^C |
| --- | --- | --- | --- | --- | --- |
| I33738 (skelet I) | petrous | PSUAMS-12655 | 5325 ± 25 | 4290-4050 | -21.34 |
| I33739 (skelet II) | collagen rib | Utc-1961 | 5190 ± 50 | 4226-3808 |  |

The new PSUAMS date (Supplementary Table 16) supports the older Utc date and suggests a date between 4200 and 4000 BCE for all skeletons.

**Isotopes:**

Enamel fragments from three maxillary molars (teeth 26, 27, and 28) belonging to Skeleton II (**sample I33739**) were available for combined Sr-O-C isotope analysis (see Supplementary Information S3). The results are presented in Table SI2.3. All ^87^Sr/^86^Sr are compatible with the expected bioavailable Sr signature in the Zoelen region, i.e., the Dutch central river area (Kootker et al., 2016). The *δ*^13^C_PDB_ values are relatively low compared to the majority of the analysed individuals from the Netherlands (all periods, unpublished data from L.M. Kootker). Such values may indicate that the female's diet during her first 16 years of life mainly consisted of C₃ plants, which were likely sourced from forested or temperate environments. The *δ*^18^O_PDB_ values vary (max-min: 1.53 ‰) and are all, except for the M^1^, comparable to the values we believe are indicative of "the Netherlands" (Kootker et al., 2019). The –7.3 ‰ of the first molar (M^1^) currently falls outside this range, which at first glance cannot be directly linked to breastfeeding, as the δ¹⁸O of the M^1^ in that case would usually be heavier (i.e., more positive) than the third molar (M^3^). It is possible that the woman spent her youth somewhat more inland, possibly to the west of present-day Germany. A limiting factor in the interpretation that should also be considered is that the variation in δ¹⁸O within a single molar can be significant (up to about 2‰ in modern molars: Plomp et al., 2020).

### Table SI2.3. Sr-O-C isotope data for Skeleton II from Zoelen-de Beldert. The element numbers are according to Fédération Dentaire Internationale (FDI).

| **Sample ID** | **Material** | **Element** | **87Sr/^86^Sr** | **2SE** | ***δ*^13^C_PDB_ (‰)** | **SD** | ***δ*^18^O _PDB_ (‰)** | **SD2** |
| --- | --- | --- | --- | --- | --- | --- | --- | --- |
| Skelet II (I33739) | Enamel | 26 | 0.708914 | 0.000007 | -14.43 | 0.05 | -7.28 | 0.06 |
|  |  | 27 | 0.708736 | 0.000007 | -15.2 | 0.05 | -5.75 | 0.06 |
|  |  | 28 | 0.708856 | 0.000008 | -15.27 | 0.07 | -6.69 | 0.04 |

**Source of the samples:** Archeologisch Depot Gelderland; Stephan Weiss-König. Samples collected by Eveline Altena.

**Authors of the entry:** Roel Lauwerier, Harry Fokkens, Lisette Kootker

**References**:

Hogestijn, J. W. H., & Lauwerier, R. C. G. M. (1992). Zoelen Beldert. *Jaarverslag Rijksdienst Voor Het Oudheidkundig Bodemonderzoek 1991*, 108.

Hulst, R. S., Hogestijn, J. W. H., de Haan, M. J. A., Lauwerier, R. C. G. M., & Marswijk, R. W. (1993). Buren Zoelen. *Jaarverslag Rijksdienst Voor Het Oudheidkundig Bodemonderzoek 1992*, 69.

Kootker, L.M., Van Lanen, R.J., Kars, H., Davies, G.R. (2016). Strontium isoscapes in the Netherlands. Spatial variations in 87Sr/86Sr as a proxy for palaeomobility, Journal of Archaeological Science: Reports 6, 1-13.

Kootker, L.M., van Lanen, R.J., Groenewoudt, B.J., Altena, E., Panhuysen, R.G.A.M., Jansma, E., Kars, H., Davies, G.R. (2019). Beyond isolation: understanding past human-population variability in the Dutch town of Oldenzaal through the origin of its inhabitants and its infrastructural connections, Archaeological and Anthropological Sciences 11, 755-775.

Lauwerier, R. C. G. M. (1993). *Zoelen—Beldert 2-5-1991; neolithische menselijke skeletten (2)* [Intern verslag Archeozoölogie / ROB 2 februari 1993].

Plomp, E., von Holstein, I.C.C., Kootker, L.M., Verdegaal-Warmerdam, S.J.A., Forouzanfar, T., Davies, G.R., (2020). Strontium, oxygen, and carbon isotope variation in modern human dental enamel, Am J Phys Anthropol 172, 586-604.

## 2.3 Nieuwegein-het Klooster location A (NGKL10) (Utrecht, Netherlands)

**Analyzed individuals:**

| I12091 | (S035_ID1) |
| --- | --- |
| I17968 | (S157_ID4) |
| I12093 | (S292_ID5) |
| I12094 | (S292_ID6) |

**Contact information:** Kirsten Leijnse, Helle Molthof, Theo ten Anscher

**Site information and excavation history:** The development of a business park, ‘Het Klooster’ in Nieuwegein, the Netherlands, fueled a large-scale archaeological project. The nowadays flat green meadows are hiding a former river system called the Wiersch, a predecessor of the current Rhine, with levees and peat-filled channels. The initial surveys indicated that the area was intensively used by Neolithic communities. One of the most promising locations, discovered by augering, was a dense scatter of archaeological indicators (pottery, flint, bone and charcoal) on a former river belt, 1,7 meters below the present-day surface. The site report, due to be published in 2025, will encompass three excavated areas; the information below is limited to the largest of these, known as 'location A'.

This Neolithic settlement was excavated in 2016 and yielded a find layer with tens of thousands of sherds, flints, animal bones and stones. Based on the typology of pottery and flint artefacts, the larger part of the site is dated to the second half of the Swifterbant culture (SW2; 4400–3900 cal BCE). In the southern part of the site there are indications for an older phase (SW1; 4900–4400 cal BCE). This latter part shows an emphasis on blade technology while relatively ‘late’ elements are absent, such as fragments of polished flint axes, triangular flint points with surface retouch, and transverse arrowheads. In contrast, the northern part of the site shows a marked shift in flint technology with a stronger emphasis on flake production, in line with SW2. The aforementioned 'late' elements are also present in this northern part and probably date to the period immediately after SW2, i.e. Pre-Drouwen TRB (3900 - 3400 cal BCE), in accordance with yet another shift in flint technology.

Most strikingly, location A yielded the remains of approximately 25 human individuals. These remains seem to be confined to three zones within the excavated area: zone 1 is located in the north of the site, zone 2 approx. 50 m to the south, and zone 3 is located another 100 m further south. Most individuals are represented by human bone fragments and teeth from the sieving residue of sampling units. Some of them probably represent burials that unfortunately were not recognized as such during the excavation, some are anciently disturbed graves, partial (re)burials or stray human remains. Five individuals were recognized as inhumations during fieldwork: three of these in close proximity in zone 2 (ID2, ID3 and ID4) and two together in a grave in zone 3 (ID5 and ID6). In addition to the five individuals in graves, a skull without a mandible (ID1) was found a few meters west of the group of ID2/3/4. The graves and the isolated skull were block lifted with the surrounding clay and transported to a hall for further investigation, to ensure dry and proper research conditions.

Grave goods were found with one individual (ID4; I12092/I17968), consisting of eight perforated cattle incisors found in proximity to the neck. Apart from these, no grave goods were found. However, the sampling units yielded three jet objects, a pendant and two beads, which were found in or near areas with human remains. The pendant found near the human remains of zone 1 measures approx. 6 x 6 cm, which makes it the second largest prehistoric jet object of the Netherlands so far. Although the three ornaments could not be directly linked to buried individuals or human remains, all three were located near zones with human remains, while the areas in-between did not yield any jet finds. It is possible that the jet objects at Nieuwegein-het Klooster were originally deposited as grave goods.

**Summary of the sampled materials:** A total of thirteen samples, derived from eleven individuals, was analyzed for aDNA, but only four of the samples, two from zone 2 and two from zone 3, all petrous bones, delivered results.

The first, from zone 2, was from the isolated skull (ID1). This skull, without teeth or a mandible, belonged to an adolescent of 14–18 years and yielded sample **I12091** (male). The second sample was taken from ID4, found about four meters to the east of the skull, an almost complete skeleton of an adolescent of 16–20 years old (sample **I17968;** female). This individual was buried in an extended supine position, and around the neck eight perforated cattle incisors were found (probably domesticated, not aurochs).

In the most southern part of the site, zone 3, one grave was discovered containing the remains of two people. The first (ID5) was an almost complete skeleton of a young female adult of 20–24 years old, buried in extended supine position. Her right arm was bent, with the hand resting near the hip bone, and her head was turned towards the right. On her right side, cradled within the bent right arm, the remains of a neonate (0–3 months) were found (ID6). Both individuals were successfully analyzed for aDNA, yielding sample **I12093** (ID5: female) and **I12094** (ID6: female). The DNA analyses not only revealed that the baby is a girl, but also that the woman and the baby are first degree relatives: mother and daughter. It is the first time for the Stone Age of the Netherlands that we are able to take family ties into account and determine the sex of such a young infant.

**Dating:**

### ***Table SI2.4*** ^14^C dated sampled individuals from Nieuwegein-het klooster

| **ID number** | **Sample material** | **Lab code** | ^14^C Age BP ± 1 σ | **95.4% cal BCE** | **δ**^13^C |
| --- | --- | --- | --- | --- | --- |
| I17968 (S157 ID4) | Cattle incisor | ICA-6954 | 5410 ± 30 | 4342-4171 |  |
| t.a.q. for ID 2/3/4 | Charred hazelnuts | ICA-5755 | 5460 ± 30 | 4356-4251 |  |
| t.p.q. for ID 5 | Charred ash | ICA-6563 | 5720 ± 30 | 4678-4458 |  |

The human bone samples from zone 2 (ID1: I12091, ID4: I17968) were radiocarbon dated between ca. 5200-4680 cal BCE. However, the δ13C values of these 14C measurements indicated a freshwater reservoir effect. Additionally, typological and stratigraphical evidence indicated that these dates are older than expected and are probably influenced by post depositional processes (see below). The flint and pottery found adjacent to the burials in zone 2 point to occupation mainly in the SW2 period (4400–3900 cal BCE). That the direct dates on human bone are too old is supported by a ^14^C measurement obtained from a perforated cattle incisor, a grave gift associated with ID4, which places this burial between 4342-4171 cal BCE. A concentration of charred hazelnuts found in the sieved layer above ID2/3/4 dated to 4356-4251 cal BCE. As these must be considered as a *terminus ante quem*, they agree with the overall stratigraphy and context of the site, and it must thus be concluded that the human remains found in zone 2 should also be placed within the early SW2 phase (ca. 4400-4250 BCE).

Of ID5 in zone 3 (sample **I12093**) two ^14^C samples dated to 4845-4726 cal BCE and 4928-4707 cal BCE. As with the samples from zone 2, the δ^13^C values indicated a freshwater reservoir effect. Nevertheless, when looking at the flint found in zone 3, this material does point towards occupation in the SW1 phase (4,900–4,400 BCE), older than the northern and mid-section of the site, although later occupation (SW2 and Pre-Drouwener TRB) is found in the southern part as well. Several ^14^C dated features in the vicinity of the buried mother and child also point to usage of this part of the terrain during SW1 (as well as later periods). However, these older features are, without exception, the remnants of surface hearths, covered by a layer of clay that has been deposited around 4200 cal BCE. The skeletal remains are found at exactly the same height as the nearby SW1 surface hearths, only a few meters away. Synchronicity can then only be assumed if the skeletons were also placed on the surface and remained there, undisturbed, for several centuries. The relatively sparse displacement of skeletal elements makes it more than likely that the bodies were buried in a shallow grave pit, dug into the clay layer that was dated to ca. 4200 cal BCE. Furthermore, below the mandible of ID5 a piece of charred ash was found, dating to 4678-4458 cal BCE. Charcoal was not abundant in the fill of the presumed grave pit. This isolated fragment is interpreted as part of the infill, originating from a fire that dates before the burial event, and is therefore seen as a *terminus post quem*. On the basis of the stratigraphy of the graves and the non-human radiocarbon dates, it is probable that ID5 and ID 6 date to ca. 4200 BC.

The ^14^C dates of human bone, proved more than problematic; most results on these materials from Nieuwegein-het Klooster (and elsewhere, such as Tiel Medel-de Roeskamp) had to be classified as unreliable. This was already expected as a result of the observed soil formation processes, in this case especially the decalcification of the uppermost part of the soil, to a depth of 50 to 100 cm below the surface. Amongst many other effects, decalcification and specifically the byproducts of calcium carbonate dissolution such as calcium oxide and calcium hydroxide, have a strong detrimental effect on bone, especially the collagen therein, but can also lead to increased exchange of elements (i.e. carbon) with the surrounding matrix (a more detailed description shall be part of the site report, including some analyses with multiple ^14^C dates from the same context using different sample materials). The mentioned processes, with these effects, are not specific to Nieuwegein-het Klooster, but can also be expected in other clay areas (fluviatile and marine) that were originally calcareous but underwent (strong) decalcification.

**Isotopes:** The tooth enamel quality of the Nieuwegein-het Klooster individuals was moderate. To minimize contamination, only dental elements that produced clean, white enamel powder during sampling were selected for analysis. As a result, only two of the four individuals included in this study, ID4 and ID5, were sampled for Sr-O-C isotope analysis (Supplementary Information S3). However, only the Sr isotope data will be presented here. For ID4, all three molars from the right maxilla were sampled, while for ID5, all three molars from the left mandible were analysed. The data are presented in Table SI2.5.
The measured ^87^Sr/^86^Sr vary significantly, both between individuals and among the molars of a single individual (0.7086–0.7096). According to the current Sr isoscape map (Kootker et al., 2016), the lowest ratios are relatively low for the region and even for the present-day Netherlands. However, in the Neolithic, Sr isotope ratios below 0.7088 were more commonly observed in the Dutch river area (unpublished data from L.M. Kootker). This suggests that such low values likely occurred frequently in the prehistoric riverine landscape. Overall, the observed Sr isotope ratios are consistent with expectations for a geologically heterogeneous region such as present-day Nieuwegein-het Klooster. In contrast, the oxygen isotope data (not given here) seem to indicate possible non-local origins. However, the obtained *δ*^18^O_PDB_ values require confirmation through δ^18^O_PO4_ analysis, as phosphate-derived values from apatite are considered less susceptible to diagenetic alteration than those from carbonate.

### Table SI2.5. Sr isotope data for female individuals ID4 and ID5 from Nieuwegein-het Klooster. The element numbers are according to Fédération Dentaire Internationale (FDI).

| **Sample ID** | **Material** | **Element** | **87Sr/^86^Sr** | **2SE** |
| --- | --- | --- | --- | --- |
| V791.1/ID4 (I17968) | Enamel | 16 | 0.708702 | 0.000009 |
|  |  | 17 | 0.709166 | 0.00001 |
|  |  | 18 | 0.708622 | 0.00001 |
| V790.3/ID5 (I12093) | Enamel | 36 | 0.709646 | 0.000009 |
|  |  | 37 | 0.709075 | 0.000008 |
|  |  | 38 | 0.709289 | 0.000009 |

The ^87^Sr/^86^Sr of female individuals ID4 (**I17968**) and ID5 (**I12093**) vary significantly among different dental elements, suggesting residential mobility during childhood or shifts in their primary food sources. For ID4, at least two periods of mobility can be identified: after the third year and around the eighth year of life. In contrast, ID5 exhibits evidence of only one mobility event, occurring after the third year. The isotopic difference between the second and third molars in ID5 is minimal (<0.0002, see Plomp et al., 2020), which is insufficient to indicate significant mobility.

**Source of the samples: RAAP archeologisch Adviesbureau.** Samples collected by Eveline Altena.

**Authors of the entry:** Kirsten Leijnse, Helle Molthof, Paul van der Kroft, Theo ten Ancher, Lisette Kootker, Steffen Baetsen.

**References:** The site is due to be published in 2025

Kootker, L.M., Van Lanen, R.J., Kars, H., Davies, G.R. (2016). Strontium isoscapes in the Netherlands. Spatial variations in ^87^Sr/^86^Sr as a proxy for palaeomobility, Journal of Archaeological Science: Reports 6, 1-13.

Plomp, E., von Holstein, I.C.C., Kootker, L.M., Verdegaal-Warmerdam, S.J.A., Forouzanfar, T., Davies, G.R. (2020). Strontium, oxygen, and carbon isotope variation in modern human dental enamel, Am J Phys Anthropol 172, 586-604.

## 2.4 Tiel Medel-de Roeskamp (NBBM6) (Gelderland, the Netherlands)

**Analyzed samples:**

| I35542 | (grave 4_S1137_V4957) |
| --- | --- |
| I35543 | (grave 1_S1147_V2084) |
| I35544 | (grave 3_S2386_V4910) |

**Contact information:** Theo ten Anscher

**Site information and excavation history:**

At Tiel Medel-De Roeskamp, in the Dutch central river district, large-scale excavations were carried out between 2016-2017. In the excavated area of approximately 5 ha, stratigraphically separated prehistoric landscapes were uncovered, revealing settlement remains dating to the Early Neolithic Swifterbant culture, the Middle Neolithic Hazendonk Group and the Early Bronze Age, with traces extending to the late Neolithic and well into the late Bronze Age. The reported age ranges are obtained from the overall chronostratigraphy of the site. For an exhaustive discussion on the dating of the site we refer to the original report (Ten Anscher *et al.* 2023). However, in this section we deviate significantly from the chronostratigraphy presented in the report on one point, namely the dating of the flatgrave cemetery. The summary presented here is reconciliation of both the chronostratigraphy of the site and the new aDNA evidence, unavailable at that time.

Situated on a crevasse/levee along a channel of the river Rhine system approximately 1,5 ha of a large Swifterbant settlement was excavated, yielding more than 550.000 finds. Around 40 ^14^C-dates place the settlement firmly in Phase SW2 (4400-3900 BCE). Occupation began around 4300 with the clearing of the local hardwood riparian forest. Over time, the rapidly rising groundwater table and the associated deposition of clay sediments in the surrounding floodplain and channel had an initially imperceptible but steadily worsening impact on the levee’s suitability as a settlement location. The area that remained above the surrounding floodplain gradually diminished. From 4050 BCE onwards, the site was only sporadically visited. By that time, the levee was largely submerged, while the local channel had become a narrow and shallow residual channel.

Approximately 30-40 two aisled, rectangular or slightly tapering house plans point to a permanent settlement consisting of at least a few houses at any one time. Evidence of local cereal cultivation is abundant, including about 2500 chaff and kernel remains of naked barley, einkorn, emmer wheat and free-threshing wheat (durum), as well as grain based products such as ground cereals and charred bread lumps. More than 75 % of all identified mammalian remains (7500 fragments identifiable to species or family level) are from livestock, dominated by large pigs kept for meat, and cattle kept for milk, meat, and as draught animals. Less than 25 % of mammalian remains belong to game, mostly red deer. In particular, bones of fur animals, abundant at other Swifterbant sites, are scarce. Fishing appears to have been substantial. Among the gathered food plants, hazelnuts and crab apples were prominent. Apart from a few scattered human remains, no traces of human graves were found on the levee. Some in situ skeletal remains indicate (partial) dog burials.

Polished stone axes and a *durchlochte Breitkeil*, all found in fragments, as well as the spectrum of grains and arable weeds, ceramics (including vessel shapes, decoration rules based on pot format, and the application of a layer on large pots), some flint tools (particularly used for harvesting) and the house types, indicate strong Bischheim influences and shared technological and agricultural practices. This agrarian lifestyle – complemented by fishing, gathering, and some hunting – persisted at Medel until local environmental conditions made it unsustainable.

Around 3800 BCE, the old silted up channel was reactivated, causing extensive erosion of the former riverbank area of the Swifterbant settlement. The sediments in the old channel were also swept away. It took about a century for new inhabitable landscape elements to form over the area of the old channel. Point bars along the new channel, which subsequently silted up rapidly, attracted Hazendonk occupation. This habitation probably started around 3700 BCE and lasted until about 3500 BCE or slightly later, yielding approximately 75.000 finds. The Hazendonk settlement is characterized by a sequence of a few houses, with one occupied at a time. The subsistence economy was dominated by agriculture as is evidenced by a small number of chaff remains and kernels of naked barley and emmer wheat, as well as livestock, mainly cattle and pigs. Ca. 25 % of the mammal bones belong to game, particularly wild boar, followed by red deer and aurochs. In contrast to the Swifterbant period, very few charred hazelnut shells were recovered, suggesting that gathering played a less significant role. Fishing appears to have been of lesser importance too.

In the extensive site monograph, no graves are mentioned for the Hazendonk period (nor for the Swifterbant period). However, new aDNA information has led to a reinterpretation of the data: a flatgrave cemetery formerly ascribed to the Early Bronze Age is now believed to be much older and should be linked to the Hazendonk period. The arguments are outlined below.

Followed by a hiatus of ca. 1500 years a new occupation phase began in the Early Bronze Age (1950-1800 cal.BC), preceded by ephemeral traces of a Late Neolithic Bell Beaker settlement. Nearly 300.000 finds can be ascribed to this period. The Early Bronze Age settlement is characterized by several farmyards and associated structures, along with three monumental burial mounds containing dozens of inhumation and cremation burials. These mounds functioned as cemeteries that were in use from the Early Bronze Age, or perhaps the late Bell Beaker period, well into the Late Bronze Age. During its early phases the largest mound was encircled by a disrupted ditch and seems to have served as a solar calendar. When viewed from a central point within the disrupted circle, the gaps between the ditches align with the position of the rising sun during solstices and equinoxes.

180 m to the southwest of the burial mounds, a small flat grave cemetery was discovered. At the center of this cemetery, precisely aligned with one of the viewing gaps, a ritual pit was discovered. This pit contained animal bones, human skull remains (without the mandible or teeth), a glass bead – the oldest glass bead in the Netherlands, probably crafted in Mesopotamia – and several Early Bronze age sherds. The four surrounding individual graves 1-4 and grave 5, which contained the remains of at least 20 individuals, all lacked datable grave gifts. Only the rather poorly preserved skeletal remains were visible, with no traces of burial pits whatsoever. The teeth (and sometimes the premolars as well) of the adults, who appeared to be exclusively female, showed significantly more wear than the molars, indicating that they were used as tools. This trait was one of the key arguments for the contemporaneity of the individuals. About twenty ^14^C-samples were taken from teeth or bone, but none yielded reliable results due to poor collagen preservation as a result of soil formation processes. These soil processes are not restricted to Medel but can be expected for large areas in the Dutch river district.

Initially a Hazendonk date for the graves was contemplated, but when it became clear that the sherds found directly above and among the human remains belong to Bell Beaker and Early Bronze Age pottery, this was deemed highly unlikely. Since the ritual pit must date to the Early Bronze Age (see also below), while its upper fill contained human remains that are complementary to the headless skeleton from the adjacent grave 4, all the indications pointed to an Early Bronze Age date for the flat grave cemetery.

Several aDNA samples from this flat grave cemetery were attempted, but only three individuals provided aDNA samples. The new aDNA evidence, not available at the time, makes it clear that an Early Bronze Age date and all the hypotheses concerning the Early Bronze Age and Beaker burial rituals, has to be rejected. A new scenario that reconciles the archaeological data with the genetic evidence is proposed below.

**Summary of the sampled materials:**

Grave 1 (I35543) contained the remains of a 40–45-year-old female in a strongly flexed position, lying on her left side. No grave goods were associated with this burial.

Grave 3 (I35544) contained the remains of a 30–50-year-old female in a flexed position, lying on her right side. An awl crafted from a metacarpal or metatarsal bone of an herbivore (sheep, goat, or deer) was found within the grave.

Grave 4 (I35542) was partly disturbed, as the skull was discovered in a separate pit adjacent to the burial. The grave itself contained the headless skeleton of a 25–35-year-old female, buried in a strongly flexed position, lying on her right side. Within the upper fill of the pit, the heavily fragmented remains of what is likely her skull were discovered, accompanied by teeth from the upper and lower jar and some bones from the hand.

**Dating:**

### Table SI2.6 ^14^C dates from Tiel Medel de Roeskamp

| **ID number** | **Sample material** | **Lab code** | ^14^C Age BP ± 1 σ | **95.4% cal BCE** | **δ**^13^C |
| --- | --- | --- | --- | --- | --- |
| *t.p.q.* grave 1 | Charred einkorn | GrM-24349 | 5243 ± 29 | 4227-3974 |  |
| Ritual pit (t.a.q. for grave 4) | Charred cereal | GrM-22836 | 3702 ± 24 | 2196-1985 |  |
| t.p.q. for the pointbar on which the cemetery is located. | Unburnt willow fruit | Ua-55971 | 4959 ± 32 | 3797-3648 |  |
|  | Charred hazelnut | GrM-17652 | 5005 ± 30 | 3945-3656 |  |

As with the Nieuwegein samples (see section 2.3), radiocarbon dates on human bone and teeth yielded conflicting results, often contradicting the stratigraphical and archaeological evidence. The dates have been reported in the site publication (Ten Anscher *et al.* 2023), while here we only present the dates that were reliably associated with the graves.

Grave 1 (I35543): a sample of the right femur yielded no collagen. A piece of the right tibia was dated to 3093-2787 cal BCE. One burnt fragment of einkorn found in grave 1 was radiocarbon dated to 4227-3974 cal BCE. The latter date should be regarded as a *terminus post quem*.

Grave 3 (I35544): a sample of the left tibia yielded no collagen. Tooth enamel (apatite) was dated to 2012-1883 cal BCE. As the radiocarbon dates on human skeletal remains kept yielding conflicting results, a new experimental method was attempted on a fragment of the left femur (apatite) providing a date of 3631-3373 cal BCE (Ten Anscher *et al*. 2023). This experimental method consisted of a pre-treatment to isolate the apatite fraction and three AMS measurements carried out on CO₂ gas rather than on graphite (Ten Anscher *et al.* 2023, p. 1350, note 30). As the experimental protocol is still unvalidated, neither date can be definitively rejected. However, in light of the IBD connections, the latter appears more plausible (see below).

Grave 4: no associated ^14^C-dates.

Similar to the three graves sequenced in this study, the other graves proved problematic to date. A full discussion on the different dating attempts for each grave, including experimental methods employed, and the context as a whole have been described in Ten Anscher *et al.* (2023).

The individuals from graves 1, 3, and 4 have no steppe ancestry, pleading against a date in the Early Bronze Age. There are multiple IBD links (Supplementary Table 14) to several individuals from predominantly England and Scotland dating to the first half of the 4th millennium BCE and an individual (**I33738**) from nearby Zoelen de Beldert (~4200-3800 BCE; see Supplementary Table 14). In particular the latter individual must be related around the 7^th^ degree to individual I35543 from Tiel Medel-de Roeskamp. The maximum distance between these individuals can thus be hypothesized to be ~225 years, assuming an average generation time of 25 years. This too is in conflict with a previously suggested Early Bronze Age date for graves 1, 2, and 3.

Reconciling the archaeological and the genetic evidence is possible, according to a new scenario: The ritual pit must date to the Early Bronze Age, as is attested by the presence of Early Bronze Age sherds, the oldest glass bead found in the Netherlands and a radiocarbon sample from cereal *parenchym* dated to 2196-1985 cal BCE (a *terminus post quem* for the digging of the ritual pit and a *terminus ante quem* for grave 4), all found in its lower fill, and it’s position in relation to the ‘calendar monument’ mentioned before. It has been cut through a much older grave, grave 4. After the rituals involving the deposition of animal bones, a glass bead and a skull, were completed, the pit was filled and the disturbed remains from grave 4 were reburied in the top of the pit.

Contrary to what was initially considered, the (fragments of) late Bell Beaker and Early Bronze Age sherds found directly on top and among the graves do not provide *termini post quem* for the graves. They should instead be regarded as later intrusions. There is evidence for erosion reaching to the top of the human remains. The combination of erosion and trampling could explain their presence.

The cemetery is located on a point bar that was formed around or after 3800 BCE as is attested by two radiocarbon dates from samples found deep in the sediments that were deposited shortly after the channel reactivation. An uncharred fruit of a willow was dated to 3797-3648 cal BCE and a charred hazelnut shell was dated to 3945-3656 cal BCE. They provide *termini post quem* for the formation of the point bar, clearly ruling out an attribution of the graves to the Swifterbant Culture.

The conclusion is that the flat grave cemetery including graves 1, 3 and 4 can be dated to the first half of the 4^th^ millennium BCE. A date around 3700 seems likely, also based on the other radiocarbon dates associated with refuse from the Hazendonk occupation period. These dates are discussed in depth elsewhere (Ten Anscher *et al*. 2023).

**Isotopes:**

### Table SI2.7. Sr-O-C isotope data for females 1, 3, and 4 from Tiel Medel-de Roeskamp. The element numbers are according to Fédération Dentaire Internationale (FDI).

| **Sample ID** | **Material** | **Element** | **^87^Sr/^86^Sr** | **2SE** | ***δ*^13^C_PDB_ (‰)** | **SD** | ***δ*^18^O _PDB_ (‰)** | **SD2** |
| --- | --- | --- | --- | --- | --- | --- | --- | --- |
| Grave 1_S1147 (I35543) | Enamel | 46 | 0.708727 | 0.000008 | -16.92 | 0.11 | -7.01 | 0.14 |
|  |  | 47 | 0.708852 | 0.00001 | -14.62 | 0.05 | -6.22 | 0.08 |
| Grave 3_S2386 (I35544) | Enamel | 46 | 0.708947 | 0.000008 | -15.16 | 0.06 | -6.21 | 0.15 |
|  |  | 47 | 0.710393 | 0.000007 | - | - | - | - |
|  |  | 28 | 0.709581 | 0.000008 | -14 | 0.17 | -6.42 | 0.2 |
| Grave 4_S1137 (I35542) | Enamel | 16 | 0.71539 | 0.000012 | - | - | - | - |
|  |  | 17 | 0.715615 | 0.000007 | -15.21 | 0.09 | -6.16 | 0.19 |
|  |  | 18 | 0.71495 | 0.000008 | -14.76 | 0.04 | -5.52 | 0.09 |

Despite the poor preservation of the skeletons, complete dental elements or enamel fragments from female individuals in Graves 1, 3, and 4 were available for combined Sr-O-C isotope analysis. However, the O-C isotope data should be interpreted with caution (see Supplementary Information S3). The results are presented in Table SI2.7.

The ^87^Sr/^86^Sr vary significantly among individuals, providing evidence of both intra-individual mobility and diverse regional origins. The Sr isotope signature of Grave 1 is consistent with the expected bioavailable Sr signature for the Tiel region, i.e., the Dutch central river area (Kootker et al., 2016). Her *δ*^18^O_PDB_ values are relatively low, possibly indicating an origin further inland than Tiel. However, the difference between the obtained *δ*^18^O_PDB_ value and the ‘local’/’Dutch’ lower limit is minimal, particularly when considering potential intra-tooth variation (Plomp et al., 2020). Female Grave 3 exhibits greater variation in ^87^Sr/^86^Sr. The first and third molars align with the local Sr isotope signature, as do the *δ*^18^O_PDB_ values, though they fall on the lower end of the expected range. The second molar, however, exhibits a more radiogenic Sr ratio (0.710393), which is inconsistent with the Dutch river area (Kootker et al., 2016) but aligns with regions to the north and south of the site where Pleistocene sandy deposits occur. While she may not have originated far from Tiel, her isotopic data indicate residential mobility during childhood. A scenario in which she left the Tiel De Roeskamp site at an early age and returned a few years later remains plausible.

The most striking results come from female Grave 4. Her ^87^Sr/^86^Sr indicate childhood mobility and are incompatible with both the Tiel region and the rest of the Netherlands. While her *δ*^18^O_PDB_ a values fall within the local or Dutch range, her Sr isotope ratios exclude a local origin. Radiogenic Sr signatures of this nature are found in southeastern Belgium (Sengeløv et al., 2025) and Scotland (British Geological Survey materials © UKRI, 2025), though a more northern origin, such as Scandinavia, cannot be ruled out. Given the IBD genetic link between the individual from Grave 4 and Scotland (Supplementary Table 14), this represents an intriguing match that warrants further investigation. However, her *δ*^18^O_PDB_ values are too positive for a British origin. Further analysis of the oxygen isotope composition in the phosphate fraction could provide more reliable insights. The *δ*^13^C_PDB_ values suggest that the females’ diets during their first 16 years of life were predominantly based on C₃ plants, likely sourced from forested or temperate environments.

**Source of the samples:** RAAP Archeologisch Adviesbureau. Samples collected by Steffen Baetsen.

**Author of the entry:** Theo ten Anscher, Lisette Kootker

Kootker, L.M., Van Lanen, R.J., Kars, H., Davies, G.R. (2016). Strontium isoscapes in the Netherlands. Spatial variations in ^87^Sr/^86^Sr as a proxy for palaeomobility, Journal of Archaeological Science: Reports 6, 1-13.

Plomp, E., von Holstein, I.C.C., Kootker, L.M., Verdegaal-Warmerdam, S.J.A., Forouzanfar, T., Davies, G.R. (2020). Strontium, oxygen, and carbon isotope variation in modern human dental enamel, Am J Phys Anthropol 172, 586-604.

Sengeløv, A., Capuzzo, G., Dalle, S., James, H.F., Sabaux, C., Stamataki, E., Hlad, M., Gerritzen, C.T., Legrand, E.M., Veselka, B., Mulder, G.D., Annaert, R., Boudin, M., Salesse, K., Warmenbol, E., Mattielli, N., Snoeck, C., Vercauteren, M. (2025). From plants to patterns: Constructing a comprehensive online strontium isoscape for Belgium (IsoBel) using high density grid mapping, Geoderma 453, 117123. https://doi.org/10.1016/j.geoderma.2024.117123

Ten Anscher, T.J., S. Knippenberg, C.M. van der Linde, W. Roessingh, N. Willemse (2023). Doorbraken aan de Rijn, Een Swifterbant-gehucht, een Hazendonk-nederzetting en erven en graven uit de bronstijd in Medel-De Roeskamp, RAAP-rapport 6519 / Archol rapport 742 / ADC rapport 6150 / BAAC Rapport A-16.0207, RAAP/Archol/ADC ArcheoProjecten/BAAC, Weesp/Leiden/Amersfoort/‘s-Hertogenbosch.

## 2.5 Schipluiden-Harnaschpolder (04HP) (Zuid-Holland, the Netherlands)

**Analyzed samples:**

| I38121 | (V9267) |
| --- | --- |
| I38447 | (Grave 5, Ind. 6) |
| I38448 | (Grave 6, Ind. 7) |

**Contact information:** Liesbeth Smits

**Site information and excavation history:** The Schipluiden site was located on a submerged sand dune near the coast, of which the top presently is situated at c. 3m minus Dutch Ordnance Datum (NAP). The site was excavated in 2003 by ARCHOL bv. in cooperation with Leiden University in artificial dry conditions (water management). It yielded a wealth of data about Middle Neolithic habitation and economy, including a palisaded settlement area, houses or huts, preservation of organic material and a small number of burials (Louwe Kooijmans & Jongste 2006). Three phases of habitation between 3800 and 3400 BCE were recognized.

**Summary of the sampled materials:** Six burials were found, which contained seven individuals in a flexed or stretched position. Apart from that, a number of ‘loose’ bones were discovered. The preservation condition was good, probably because originally the dead were buried close to the groundwater table and became submerged soon after habitation (Smits & Louwe Kooijmans 2006: 93). Grave 1 contained the remains of two male individuals, both in extended supine position. Ind. 2 had most likely died from a blow to the front side of the head. For Ind. 1 there were no indications of traumata (Smits & Louwe Kooijmans 2006: 95). Nevertheless, an unnatural death for both individuals may be the reason for the unusual nature of this burial (double and (semi) extended position vs. single and flexed or strongly flexed) (Smits & Louwe Kooijmans 2006: 95).

Individuals 3, 4, 5, 6, and 7 were buried in individual graves in a strongly flexed position, indicative of being tightly wrapped or bound. Individual 6 and 7 (grave 5 and 6 respectively) were young children, Individual 6 yielded sample **I38447,** Individual 7 yielded **sample I38448**. Apart from these six formal burials, the site yielded 36 human bones without burial context, belonging to at least eight individuals (Smits *et al.* 2010). One of these, upper molar **V09267,** yielded sampled (**I38121**)**.**

The strontium isotopes of the individuals (Table SI2.8) showed no signs for mobility, but the Oxygen δ^18^O levels showed that two of the bones without context (V05001 at 15.8‰ and V08057 at 18.9‰) could not have been local (Smits *et al.* 2010: 20). In their words: “Using the calibration of Daux et al. (2008) shows that SCH6 *(=V05001)* must have spent his childhood somewhere well to the east or south with a drinking water δ^18^O of -9.4 ± 0.5‰ , which is consistent with modern precipitation in a broad band from central Scandinavia, through eastern and southern Germany to the western Alps.” (Smits *et al.* 2010: 20, 21).

**Dating:**

The human bones yield slightly older dates than the proposed period of habitation, but the δ^13^C values indicate a reservoir effect (Mol *et al.* 2006). In a recent study the source for this reservoir effect has been identified as likely coming from cattle grazing in saltmarsh environments although it is not possible to accurately account for this effect (Kamjan *et al*. 2020). From the archaeological evidence it is clear the habitation ended before 3400 BCE, because no Vlaardingen pottery was recorded, only Hazendonk pottery, which was in use until the first half of the fourth millennium BCE (Mol *et al.* 2006: 35; Raemaekers & Rooke 2006).

**Source of the samples:** Provinciaal archeologisch depot Zuid-Holland; Inge Riemersma, Mark Phlippeau. Samples collected by Eveline Altena.

**Authors of entry:** Liesbeth Smits, Harry Fokkens

**References:**

Louwe Kooijmans, L. P., & Jongste, P. F. B. (2006). *Schipluiden. A neolithic settlement on the Dutch North Sea coast, c. 3500 cal BVC* (Vol. 37/38). Faculty of Archaeology.

Mol, J., Louwe Kooijmans, L. P., & Hamburg, T. D. (2006). 2 Stratigrahy and chronology of the site. In L. P. Louwe Kooijmans & P. F. B. Jongste (Eds.), *Schipluiden: A Neolithic Settlement on the Dutch North Sea Coast c. 3500 cal BC* (pp. 19–38). Leiden University Press.

Kamjan S, Gillis RE, Çakırlar C, Raemaekers DCM (2020) Specialized cattle farming in the Neolithic Rhine-Meuse Delta: Results from zooarchaeological and stable isotope (δ^18^O, δ^13^C, δ^15^N) analyses. *PLOS ONE 15(10)*: e0240464. https://doi.org/10.1371/journal.pone.0240464

Raemaekers, D. C. M., & Rooke, M. (2006). The Schipluiden pottery. In L. P. Louwe Kooijmans & P. F. B. Jongste (Eds.), *Schipluiden: A Neolithic Settlement on the Dutch North Sea Coast c. 3500 cal BC* (pp. 113–128). Leiden University Press.

Smits, E., & Louwe Kooijmans, L. P. (2006). 5 Graves and human remains. In L. P. Louwe Kooijmans & P. F. B. Jongste (Eds.), *Schipluiden: A Neolithic Settlement on the Dutch North Sea Coast c. 3500 cal BC* (pp. 91–112). Leiden University Press.

Smits, E., Millard, A., Nowell, G., & Pearson, G. (2010). Isotopic Investigation of Diet and Residential Mobility in the Neolithic of the Lower Rhine Basin. *European Journal of Archaeology*, *13*, 5–31. https://doi.org/10.1177/1461957109355040

## 2.6 Molenaarsgraaf (Zuid-Holland, the Netherlands)

**Analyzed sample:**

I13025 (h 1967/1.215/158_skelet I)

I13026 (h 1967/1.208_skelet II)

I13027 (h 1967/1.?-5_skelet III)

### Table SI2.8 samples used from Molenaarsgraaf individuals

| **ID number** | **Sample material** | **Lab code** | ^14^C Age BP ± 1 σ | **95.4% cal BCE** | **δ**^13^C |
| --- | --- | --- | --- | --- | --- |
| I13025 (h 1967/1.215/158_Skelet I; grave I) | collagen bone | GrN-5131 | 3635 ± 40 | 2136-1892 | - |
| I13026 (h 1967/1.208_Skelet II; grave II) | collagen bone | GrN-5566 | 3640 ± 40 | 2136-1897 | - |
| I13027 (h 1967/1.?-5_Skelet III; grave III) | petrous | PSUAMS-7847 | 3700 ± 25 | 2197-1983 | - |

These samples have previously been reported in Patterson, N., Isakov, M., Booth, T. et al. Large-scale migration into Britain during the Middle to Late Bronze Age. Nature 601, 588–594 (2022). https://doi.org/10.1038/s41586-021-04287-4.

## 2.7 Molenaarsgraaf-24A (Zuid-Holland, the Netherlands)

**Analyzed sample:**

| I12896 | (24A_h 1973/3_18,19) |  |
| --- | --- | --- |

**Contact information:** Luc Amkreutz, Leendert Louwe Kooijmans

**Site information and excavation history:** In August 1972 local archaeologists discovered human bones in the side of a newly cleaned ditch. They reported this to the keeper of the National Museum of Antiquities (L.P. Louwe Kooijmans). On a Saturday afternoon with two technicians of the museum, Louwe Kooijmans rescued the remains as well as possible. They documented two concentrations of human bone c. 60 cm below the surface, situated on the sediments of the Schoonrewoerd stream ridge. There was no (grave) pit visible, and the bones were reported not to be in anatomical position (museum diary for find number 1973/3), even though Louwe Kooijmans remembers a person in crouched position. A few flints were recovered from the site as well, no pottery. The site was never published in detail but mentioned as site 24a by Louwe Kooijmans in his dissertation (Louwe Kooijmans 1974).

**Summary of the sampled materials:** The material of site 24A was inventoried and analyzed by Hilde Uytterschaut (unpublished document), who suggests that 3 individuals were present in this collection of bones, mainly based on the dental elements. There was 1 adult at least and two children (4-7 y. old). A tooth from the upper jaw of one adult individual of 30-40 years old (RM1973/18-19) yielded sample **I12896**.

**Dating:**

### Table SI2.9 ^14^C dated sampled individuals from Molenaarsgraaf-24A

| **ID number** | **Sample material** | **Lab code** | ^14^C Age BP ± 1 σ | **95.4% cal BCE** | **δ**^13^C |
| --- | --- | --- | --- | --- | --- |
| I12896 (Mol24A 1 1973/3_18,19) | apatite tooth | PSUAMS-7806 | 4100 ± 30 | 2864-2500 | -22.005 |

**Source of samples**: National Museum of Antiquities Leiden; Luc Amkreutz. Sample collected by Eveline Altena.

**Authors of entry**: Luc Amkreutz, Harry Fokkens, Leendert Louwe Kooijmans

**References**:

Louwe Kooijmans, L.P. (1974). The Rhine/Meuse Delta; four studies on its prehistoric occupation and Holocene geology. Leiden: Instituut voor Prehistorie.

## 2.8 Opmeer-Mienakker (OPM'90) (Noord-Holland, the Netherlands)

**Analyzed samples:**

| I12902 | (R9560-11_V2764, S54) |  |
| --- | --- | --- |

**Contact information:** Harry Fokkens

**Site information and excavation history:** The site of Opmeer-Mienakker was excavated in 1990 but published only in 2013 by a multidisciplinary team of Groningen University, Leiden University and the Cultural Heritage Agency of the Netherlands (Kleijne *et al.* 2013). This team re-analysed all documentary and material culture evidence but was not involved in the original excavation. The spatial analysis of the site was carried out by Nobles. He describes the grave of OPM90, nicknamed by the excavators as ‘Cees’, as deposited in a pit that was dug into the habitation layer of the site (Nobles 2013, 36; Figure 1). There were no grave goods. Stratigraphically the burial is interpreted as one of the most recent elements at the site.

In his spatial analysis Nobles reconstructs a house-like ritual structure around this grave (Nobles 2013b, esp. 238 ff.). However, there are serious doubts about that reconstruction (Fokkens et al. 2016, 76). Nobles has tried to settle the debate (Nobles 2020), but not convincingly in our view.

**Summary of the sampled materials:** Skeleton R9560-11_V2764, S54 yielded **sample I12902**; he was 20-25 years old, was approximately 1.72 m tall and there were no indications of illnesses (Pasveer et al. 1992, 270). The grave was only 120 cm long and 96 cm wide. The bottom was 25 cm below the excavation surface (Nobles 2013a, 33). Because the skeleton was partly in anatomical position, especially with respect to the labile joints of the left hand, Plomp concludes that it was probably a primary burial (Plomp 2013, 176). Strangely enough, both legs and the right arm were missing. The original examiners of the skeleton, Pasveer and Uytterschout, state that gnaw-marks were present of a dog or wolf especially on the knee joints (femur and tibia) and on the pelvis (Pasveer & Uytterschaut 1992: 274; Plomp 2013: 178). Moreover, the clavicle was broken, but no gnaw marks were reported. In other words: the right arm was (forcefully?) removed, and the lower legs were bitten off. According to Plomp this happened not very long after death because the labile hand joints were still in anatomical position (Plomp 2013, 176).

Given the ‘functional’ dimensions of the grave pit (just large enough to fit the torso), the possibility remains that this was the grave of a person who died elsewhere and the body was exposed to open air for a short time , accessible to scavengers.

**Dating:**

### Table SI2.10 ^14^C dated sampled individuals from Opmeer-Mienakker

| **ID number** | **Sample material** | **Lab code** | ^14^C Age BP ± 1 σ | **95.4% cal BCE** | **δ**^13^C |
| --- | --- | --- | --- | --- | --- |
| I12902 (R9560-11_V2764, S54) | Tooth | PSUAMS-8438 | 4000 ± 30 | 2864-2500 | -17.48 |
| I12902 (R9560-11_V2764, S54) | collagen bone | GrA-15698 | 4010 ± 50 | 2844-2348 | -18.40 |

From the stratigraphy of the site, it is clear that the grave ‘*…was dug in the final stages of human activity ...*’ (Nobles 2013a, 33). Kleijne and Weerts distinguish two possible phases of habitation, the first starting in Furholts phase D (2880-2680 BCE; (Furholt 2003; Kleijne et al. 2013), the second in Furholts phase E (2620-2480 BCE; (Furholt 2003; Kleijne et al. 2013)). The grave was dug in the second phase. The skeleton was directly dated between 2844 and 2348 cal BCE. An earlier sample date, also of bone collagen (GrA-1670 3890±50 BP), was rejected because that and others from the same batch appeared too young (Lanting et al. 2002, 76–77).

**Isotopes:** 'Cees' is currently part of the permanent exhibition at the museum that is part of the *Provinciaal Depot van Noord-Holland* in Castricum. To ensure minimally invasive sampling, three molars from both the maxilla and mandible were collected *in situ*. The results of the Sr-O-C isotope analysis are presented in Table SI2.12.
The Sr and O isotope data align with the expected values for the Opmeer-Mienakker region (West-Friesland) and the broader Netherlands, respectively (Kootker et al., 2016; Kootker et al., 2019). This suggests that 'Cees' possibly spent the first 16 years of life in a single location, possibly within West Friesland.
The δ^13^C values show some variation (–12 ‰ to –10 ‰). The –10 ‰ value is relatively high compared to other data from the Netherlands for this period. Such elevated values could be attributed to the consumption of C₄ plants or fish. Given that a potential reservoir effect has also been detected in the bone collagen, it is plausible that fish consumption, possibly for a limited period during childhood, contributed to this more positive δ^13^C signature.

### Table SI2.11. Sr-O-C isotope data for 'Cees' (S54) from Opmeer-Mienakker. The element numbers are according to Fédération Dentaire Internationale (FDI).

| **Sample ID** | **Material** | **Element** | **^87^Sr/^86^Sr** | **2SE** | ***δ*^13^C_PDB_ (‰)** | **SD** | ***δ*^18^O _PDB_ (‰)** | **SD2** |
| --- | --- | --- | --- | --- | --- | --- | --- | --- |
| OPM’90/S54 (I12902) | Enamel | 26 | 0.709148 | 0.000006 | -12.57 | 0.09 | -5.44 | 0.15 |
|  |  | 47 | 0.709181 | 0.000006 | -10.89 | 0.08 | -5.79 | 0.1 |
|  |  | 48 | 0.70923 | 0.000008 | -12.11 | 0.06 | -5.53 | 0.15 |

**Source of samples:** Provinciaal depot voor archeologie van Noord-Holland; Martin Veen, Rob van Eerden. Samples collected by Eveline Altena

**Author of entry:** Harry Fokkens, Eveline Altena, Lisette Kootker

**References:**

Fokkens, H., Steffens, B.J.W., & van As, S.F.M. (2016). Farmers, fishers, fowlers, hunters. Knowledge generated by development-led archaeology about the Late Neolithic, the Early Bronze Age and the start of the Middle Bronze Age (2850 - 1500 cal BC) in the Netherlands. Amersfoort: Rijksdienst voor het Cultureel Erfgoed.

Furholt, M. (2003). Die absolutchronologische Datierung der Schnurkeramik in Mitteleuropa und Südskandinavien. Bonn: Dr. Rudolf Habelt GMBH.

Kleijne, J.P., O. Brinkkemper, R.C.G.M. Lauwerier, B.I. Smit & E.M. Theunissen. 2013. A Matter of Life and Death at Mienakker (the Netherlands). Late Neolithic Behavioural Variability in a Dynamic Landscape. Vol. 45 (Nederlandse Archeologische Rapporten 45). Amersfoort: Cultural Heritage Agency of the Netherlands.

Kleijne, J.P., & Weerts, H.J.T. (2013). 2 Landscape and chronology. In J. P. Kleijne, O. Brinkkemper, R. C. G. M. Lauwerier, B. I. Smit, & E. M. Theunissen (eds) A Matter of Life and Death at Mienakker (the Netherlands). Late Neolithic Behavioural Variability in a Dynamic Landscape.Nederlandse Archeologische Rapporten, 19–28. Amersfoort: Cultural Heritage Agency of the Netherlands

Kootker, L.M., Van Lanen, R.J., Kars, H., Davies, G.R. (2016). Strontium isoscapes in the Netherlands. Spatial variations in ^87^Sr/^86^Sr as a proxy for palaeomobility, Journal of Archaeological Science: Reports 6, 1-13. 10.1016/j.jasrep.2016.01.015

Kootker, L.M., van Lanen, R.J., Groenewoudt, B.J., Altena, E., Panhuysen, R.G.A.M., Jansma, E., Kars, H., Davies, G.R. (2019). Beyond isolation: understanding past human-population variability in the Dutch town of Oldenzaal through the origin of its inhabitants and its infrastructural connections, Archaeological and Anthropological Sciences 11, 755-775. 10.1007/s12520-017-0565-7

Lanting, J.N., & van der Plicht, J. (2002). De ^14^C Chronologie van de Nederlandse Pre- en Protohistorie III: Neolithicum. Palaeohistoria 41/42 (1999-2000): p.1–110.

Nobles, G.R. (2013a). 3 Features. In J. P. Kleijne, O. Brinkkemper, R. C. G. M. Lauwerier, B. I. Smit, & E. M. Theunissen (eds) A Matter of Life and Death at Mienakker (the Netherlands). Late Neolithic Behavioural Variability in a Dynamic Landscape.Nederlandse Archeologische Rapporten 45, 29–36. Amersfoort: Cultural Heritage Agency of the Netherlands

Nobles, G.R. (2013b). 11 Spatial analysis. In J. P. Kleijne, O. Brinkkemper, R. C. G. M. Lauwerier, B. I. Smit, & E. M. Theunissen (eds) A Matter of Life and Death at Mienakker (the Netherlands). Late Neolithic Behavioural Variability in a Dynamic Landscape.Nederlandse Archeologische Rapporten 45, 185–240. Amersfoort: Cultural Heritage Agency of the Netherlands

Nobles, G.R. (2020). Settling the monumental issue in the Dutch Wetlands. In A. B. Gebauer, L. Sørensen, A. Teather, & A. C. Valera (eds) Monumentalising Life in the Neolithic: Narratives of Continuity and Change, 125–138. Oxford: Oxbow Books Available at: https://doi.org/10.2307/j.ctv13pk66m.16.

Pasveer, J.M., & Uytterschaut, H.T. (1992). Twee Laat-Neolithische skeletten uit Noord-Holland, een fysisch-anthropologisch onderzoek. Westerheem 41: p.268–275.

Plomp, E. (2013). 10 The human skeleton. In J. P. Kleijne, O. Brinkkemper, R. C. G. M. Lauwerier, B. I. Smit, & E. M. Theunissen (eds) A Matter of Life and Death at Mienakker (the Netherlands). Late Neolithic Behavioural Variability in a Dynamic Landscape. Nederlandse Archeologische Rapporten 45, 175–184. Amersfoort: Cultural Heritage Agency of the Netherlands

## 2.9 Sijbekarspel-Op de Veken (Noord-Holland, the Netherlands)

**Analyzed samples:**

| I33741 | ( HvH 8077-01): |  |
| --- | --- | --- |

**Contact information:** Harry Fokkens

**Site information and excavation history:** The site Op de Veken in Sijbekarspel was discovered through corings in 1986 and surveyed in more detail in 1987. In that year also four 1x2m test pits were dug. One of these was extended to 4x4m when a grave was discovered (van Heeringen & Theunissen, 2001). The site was preliminary published by Hogestijn (Hogestijn & Woltering, 1990), and a detailed analysis of the skeleton was performed (Pasveer & Uytterschaut, 1992b, 1992a). As most of the skeletons found in West-Frisia were given names by their excavators, the Sijbekarspel-Op de Veken skeleton was nicknamed ‘Mies, het woiffie van Soibekarspel’ (Hogestijn & Woltering, 1990). IBD analysis (Supplementary Table 14) made clear that I33471 shared a distant kinship (approximately 6^th^-7^th^ degree) with individual I12902 from Opmeer-Mienakker.

**Summary of the sampled materials:** The Sijbekarspel-Op de Veken skeleton yielded **sample I33741**; she was a woman aged about 30-35. She was placed on her left side facing south in a crouched position. In her youth she probably had severe health problems, a.o. a shortage of nutrients in her food (Pasveer & Uytterschaut, 1992a, p. 271). Her teeth had remains of dental plaque showing a diet of grain, and even diatoms from a salt to brackish environment, consistent with the West-Frisian wetland landscape at the time (Pasveer & Uytterschaut, 1992a, p. 273).

**Dating:**

### Table SI2.12 ^14^C dated sampless from Sijbekarspel-Op de Veken

| ID number | Sample material | Lab code | ^14^C Age BP ± 1 σ | 95.4% cal BCE | δ^13^C |
| --- | --- | --- | --- | --- | --- |
| I33741 ( HvH 8077-01) | XAD amino acids petrous | PSUAMS-12657 | 4015 ± 20 | 2575-2471 | -18.74 |
| Occupation layer | Charred hazelnut shells | GrA-107 | 3960 ± 60 | 2627-2213 |  |

A first AMS-date of bone collagen yielded an unexpectedly young date: GrA-1644 3550±50 BP. Since this date belonged to a small series of anomalous radiocarbon dates from the Groningen lab, it was re-dated (Lanting & van der Plicht, 2002, p. 76). This time it yielded 3890±50 BP (GrA-15696; 2554-2202 cal BCE). The δ^13^C value of –20. 0 ‰ and the δ^15^N value of 14.1 ‰ does not suggest a reservoir effect (Lanting & van der Plicht, 2002, p. 76). Charred hazelnut shells from the site itself yielded a slightly older date: 2627-2213 cal BC (van Heeringen & Theunissen, 2001). The stratigraphy of the site places the grave in the Vlaardingen/Corded Ware period of use, most likely slightly around 2500 BCE. Part of the petrous bone was radiocarbon dated again after sampling for genetic material. Due to the smaller error on the latter ^14^C measurement we could confirm this grave dates to the end of Corded Ware (2575-2472 cal BCE).

**Isotopes:** Comparable to her distant kin from Opmeer-Mienakker, Mies’ ⁸⁷Sr/⁸⁶Sr aligns with the expected bioavailable local strontium signature (Kootker et al. 2016, Table SI2.14). The inter-dental elemental variation in ⁸⁷Sr/⁸⁶Sr is negligible, as is the case for δ¹³C values, which indicate a diet primarily based on C₃ plants. In contrast, the δ¹⁸O data exhibit significant variation. The M^₁^ displays the most enriched δ¹⁸O value, which may be an artefact of breastfeeding. While this pattern, where earlier mineralizing dental elements show less negative δ¹⁸O values, does not occur in all individuals, it remains a plausible explanation for the observed variation. The δ¹⁸O values of the M^₂^ and M^₃^ are relatively low, even lower than those of 'Cees’. Although the δ¹⁸O of the M^₂^ exceeds the defined lower limit, the values may still be compatible with the isotopic range of “West-Friesland”. However, further research is necessary to assess the reliability of these data before drawing more definitive conclusions.

### Table SI2.13. Sr-O-C isotope data for 'Mies' (S54) from Sijbekarspel-Op de Veken. The element numbers are according to Fédération Dentaire Internationale (FDI).

| **Sample ID** | **Material** | **Element** | **^87^Sr/^86^Sr** | **2SE** | ***δ*^13^C_PDB_ (‰)** | **SD** | ***δ*^18^O _PDB_ (‰)** | **SD2** |
| --- | --- | --- | --- | --- | --- | --- | --- | --- |
| 8077-01 (I33741) | Enamel | 16 | 0.709075 | 0.000008 | -12.97 | 0.04 | -4.85 | 0.05 |
|  |  | 17 | 0.709181 | 0.000008 | -12.24 | 0.03 | -7.69 | 0.15 |
|  |  | 18 | 0.709173 | 0.000006 | -12.83 | 0.08 | -6.42 | 0.11 |

Source of samples: Provinciaal depot voor archeologie van Noord-Holland; Martin Veen, Rob van Eerden. Sample collected by Eveline Altena.

Author of entry: Harry Fokkens, Lisette Kootker

**References:**

Hogestijn, J. W. H., & Woltering, P. J. (1990). ‘Het woiffie van Soibekarspel’: Een Laat-Neolithisch vrouwengraf te Sijbekarspel. *West-Frieslands Oud En Nieuw*, *57*, 152–164.

Lanting, J. N., & van der Plicht, J. (2002). De ^14^C Chronologie van de Nederlandse Pre- en Protohistorie III: Neolithicum. *Palaeohistoria*, *41/42 (1999-2000)*, 1–110.

Pasveer, J. M., & Uytterschaut, H. T. (1992a). Twee Laat-Neolithische skeletten uit Noord-Holland, een fysisch-anthropologisch onderzoek. *Westerheem*, *41*, 268–275.

Pasveer, J. M., & Uytterschaut, H. T. (1992b). Two Late Neolithic human skeletons, a recent discovery in the Netherlands. *International Journal of Osteoarchaeology*, *2*(1), 1–14. https://doi.org/10.1002/oa.1390020102

van Heeringen, R. M., & Theunissen, E. M. (2001). *Kwaliteitsbepalend onderzoek ten behoeve van duurzaak behoud van neolithische terreinen in West-Friesland en de Kop van Noord-Holland* (Vol. 21). ROB.

## 2.10 Oostwoud-Tuithoorn (Noord-Holland, the Netherlands)

**Analyzed samples:**

I39210 (skeleton 233)

I39211 (skeleton 239)

**Contact information:** Harry Fokkens

**Site information and excavation history:** In 1956 and 1957, Prof. van Giffen excavated one Late Neolithic and one Early Bronze Age burial mound at Oostwoud-Tuithoorn (van Giffen 1961, 1962). Since van Giffen had not been able to finish the excavations, additional research was conducted in 1963 and 1966 by de Weerd (de Weerd 1963, 1967). Finally in 1977 and 1978, when the site was being threatened by deep-ploughing, a large-scale excavation was conducted under supervision of van der Waals and Lanting (Lanting 1979, 2008). During these excavations a total of 15 well preserved skeletons were recovered, dating to three episodes of activity between c. 2340–1780 cal BCE. A detailed account of all excavations was published by Fokkens et al. (Fokkens *et al.* 2017), and aDNA samples of other skeletons were published earlier by Olalde et al. (Olalde *et al.* 2018) and Patterson *et al.* 2021.

**Summary of the sampled materials:** For the present study an additional individual from the site was sampled. Skeleton 239 yielded **sample I39211**. This skeleton was of a relatively tall male individual (181.4 ± 3.27 cm) who was buried in extreme flexed position, probably because he was wrapped in a mat of some kind (Fokkens et al. 2017, 141). Skeleton 233 (**sample I39210**) was buried in a pit that was disturbed in the Middle Ages, leaving less than 25 % of the skeleton (the skull and part of the pelvis: Veselka 2016). Osteoarchaeological analysis indicated that the individual was a male of 36-49 years old (Veselka 2016), though earlier analysis had suggested this to be possibly female (Runia 1987). S**ample I39210** of skeleton 233, however, indicated the molecular sex as female.

Dating:

### Table SI2.14 ^14^C dated sampled individuals from Oostwoud-Tuithoorn

| **ID number** | **Sample material** | **Lab code** | ^14^C Age BP ± 1 σ | **95.4% cal BCE** | **δ**^13^C |
| --- | --- | --- | --- | --- | --- |
| I39211 (skeleton 239) | collagen bone | GrA-15601 | 3520 ± 60 | 2026-1688 | -20.09 |

Skeleton 239 (I39211) was dated to the Early Bronze Age, though still buried in a Late Neolithic tradition in terms of position and orientation. Therefore, we suggest he dates between 1900 and 1800 BCE. Skeleton 233 was not direct dated, but since IBD analysis shows she was buried near close relatives, including her son S242 (^14^C dated between 2281 and 1899 cal BCE (GrA- 15597: 3690 ± 60 BP)) this skeleton is dated to 2200-1900 BCE .

**Source of samples:** Provinciaal depot voor archeologie van Noord-Holland; Martin Veen, Rob van Eerden. Samples collected by Eveline Altena.

**Author of entry**: Harry Fokkens

**References**:

De Weerd, M.D. 1963. Protocolboek opgraving Oostwoud. Instituut voor Prae- en Protohistorie, Universiteit van Amsterdam.

De Weerd, M.D. 1967. Medemblik [nabij Oostwoud]. *Nieuwsbulletin KNOB* 1967, 2e afl. februari, kolom *31-*32.

Fokkens, H., Veselka, B., Bourgeois, Q., Olalde, I., & Reich, D. (2017). Excavations of Late Neolithic arable, burial mounds and a number of well-preserved skeletons at Oostwoud-Tuithoorn; a re-analysis of old data. *Analecta Praehistorica Leidensia*, *47*, 95–150.

Lanting, J. N. (1979). Medemblik: Oostwoud. *Archeologische Kroniek van Noord-Holland over 1978*, 250–251.

Lanting, J. N. (2008). De NO-Nederlandse/NW-Duitse Klokbekergroep: Culturele achtergrond, typologie van het aardewerk, datering, verspreiding en grafritueel. *Palaeohistoria* 49/50 (2007-2008): 11–326.

Olalde, I. et al. (2018). The Beaker phenomenon and the genomic transformation of northwest Europe. *Nature* 555: 190–96. https://doi.org/10.1038/nature25738 https://www.nature.com/articles/nature25738#supplementary-information.

Patterson, N. *et al*. (2021). Large-scale migration into Britain during the Middle to Late Bronze Age. *Nature* 601: 588–94. https://doi.org/10.1038/s41586-021-04287-4.

Runia, L.T. (1987). *The chemical analysis of prehistoric bones. A paleodietary and ecoarcheological study of Bronze Age West-Friesland* (British Archaeological Reports International Series 363). Oxford: BAR publishing.

Van Giffen, A.E. (1961). Settlement traces of the Early Bell Beaker Culture at Oostwoud (N.H.). *Helinium* 1: 233–228.

Van Giffen, A.E. (1962). Grafheuvels uit de midden-bronstijd met nederzettingssporen van de Klokbekercultuur bij Oostwoud. *West-Frieslands Oud en Nieuw* 29: 199–209.

Veselka, B. (2016). Fysisch antropologische analyse van het menselijk skeletmateriaal uit Oostwoud. Leiden.

## 2.11 Ottoland-Kromme Elleboog (Zuid-Holland, the Netherlands)

### Table SI2.15 samples used from Ottoland-Kromme Elleboog individuals

| **ID number** | **Sample material** | **Lab code** | ^14^C Age BP ± 1 σ | **95.4% cal BCE** | **δ**^13^C |
| --- | --- | --- | --- | --- | --- |
| I12900 (h 1982/7._4_Skelet II) | collagen bone | GrN-6384 | 3820 ± 45 | 2456-2141 | - |
| I13028 (h 1982/7._4_Skelet I) |  |  |  |  |  |

These two individuals have previously been reported in Patterson, N., Isakov, M., Booth, T. et al. Large-scale migration into Britain during the Middle to Late Bronze Age. Nature 601, 588–594 (2022). https://doi.org/10.1038/s41586-021-04287-4. We produced additional genetic data for I12900.

## 2.12 Doggerland (the Netherlands)

### Table SI2.16 samples used from Doggerland individuals

| **ID number** | **Sample material** | **Lab code** | ^14^C Age BP ± 1 σ | **95.4% cal BCE** | **δ**^13^C |
| --- | --- | --- | --- | --- | --- |
| DOG007 (U 2014/12.3) |  | GrA-11642 | 8370 ± 50 | 7576-7201 | -15.4 |
| DOG001 (V002) | collagen bone | MAMS-48201 | 8627 ± 35 | 7730-7586 | - |
| DOG002 (U 2014/12.4) |  | MAMS-34582 | 9091 ± 37 | 8421-8238 | - |

A detailed archaeological context was published in Posth, C., Yu, H., Ghalichi, A., Rougier, H., Crevecoeur, I., Huang, Y., Ringbauer, H., Rohrlach, A. B., Nägele, K., Villalba-Mouco, V., Radzeviciute, R., Ferraz, T., Stoessel, A., Tukhbatova, R., Drucker, D. G., Lari, M., Modi, A., Vai, S., Saupe, T., … Krause, J. (2023). Palaeogenomics of Upper Palaeolithic to Neolithic European hunter-gatherers. *Nature*, *615*(7950), 117–126. https://doi.org/10.1038/s41586-023-05726-0

## 2.13 Swifterbant-S2 (Dronten, Flevoland, the Netherlands)

**Analyzed individuals:**

SWA001 (Skelet II)

SWA002 (Skelet III)

SWA004 (Skelet IV)

**Contact person:** Daan Raemaekers

**Site information and excavation history:**

The archaeological sites near Swifterbant were discovered in the early 1960’s as a result of the creation of the polder *Oostelijk Flevoland*. There are two landscapes in which sites were found. The first is that of sandy ridges that were located along the small river Hunnepe. These sand ridges were occupied during the Mesolithic and Neolithic and have in general a poor preservation condition. Nevertheless, 13 human burials were documented at location S21-S24 (Meiklejohn & Constandse-Westermann 1979), of which 12 were ^14^C dated, between 4600-4000 cal BCE (Raemaekers et al. 2014). Visual inspection of these remains by E. Altena resulted in their exclusion from the current analysis. The second landscape with archaeological sites is that of the levees that accompanied the Hunnepe river. Here, the sites S2, S3 and S4 are of relevance.

The sites are located on one small river system. Swifterbant-S3 and S4 are within meters distance from one another, while Swifterbant-S2 is located at some 500 m distance. Until recently, the sites were dated c. 4300-4300 cal BCE, a relatively unprecise date because of the presence of a plateau in the calibration curve. Now, the chronology of S3 and S4 has been re-analyzed, making use of short-lived samples of cereal grains, high-precision AMS dating and Bayesian modelling. As a result, S4 is now dated 4240-4160 BCE and S3 is now dated 4180-4030 BCE (Dreshaj et al. 2024).

Swifterbant-S2 was not included in this re-analysis. What can be said about the date of the site and the human burials that were documented? The single ^14^C date from the settlement is on charcoal (4309-3992 cal BCE: GrN-5443. 5300 ± 40 BP). Because this date fits the Swifternat-S3 dates perfectly and Swifterbant-S2 is located on the same river system, we propose that the site of SWIFTERBANT-S2 dates between 4180-4030 BCE as well. During fieldwork, the contours of the burial pits could be seen to have been dug through the settlement layer, implying a younger date than the settlement activities.

Detailed subsistence data are available for all three sites. They make clear that the Swifterbant occupants practiced hunting and husbandry, gathering and cultivation. Here, we focus on those elements that underline that these sites should be interpreted as Early Neolithic, rather than Late Mesolithic. Evidence for cereal cultivation is abundant. At all three sites, cultivated fields were documented (Huisman & Raemaekers 2014), charred cereal remains were found in large numbers (Van Zeist & Palfenier-Vegter 1981; Schepers 2020), many ceramic vessels were present (Raemaekers et al.2014), and all coprolites (Kubiak-Martens and Van der Linden 2022) yielded microscopic or chemical evidence of cereal remains. While most mammal bones at these sites come from pigs, the domestic status remains ambiguous. Cattle bones are found in smaller numbers and testify to full control over fodder, mobility (Brusgaard et al. 2024) and reproduction (Erven et al. in prep.). This indicates the social importance of domestic cattle to these people.

**Summary of the sampled materials:**

The human remains from SWIFTERBANT-S2 comprise ten burials in nine burial pits. The burials share a NNW-SSE orientation and are found on the eastern levee of the Hunnepe river. The age and gender distributions based on osteological analysis are as follows (Meiklejohn & Constandse-Westermann 1979):

Skelet II (sample SWA001) Female (probable) 35+ years

Skelet III (sample SWA002) Male (probable) 20-35 years

Skelet IV (sample SWA003) Male 20-55 years

The osteological sex estimations are in concordance with the genetic sex estimations.

**Source of the samples:** Provinciaal Archeologisch Depot Flevoland; Tineke Heise-Roovers. Samples collected by Eveline Altena.

**Author of the entry**: Daan Raemaekers

**References:**

Brusgaard, N. Ø., Kooistra, J., Schepers, M., Dee, M., Raemaekers, D., & Çakırlar, C. (2024). Early animal management in northern Europe: Multi-proxy evidence from Swifterbant, the Netherlands. *Antiquity*, *98*(399), 654–671. https://doi.org/10.15184/aqy.2024.58

Meiklejohn, C., & Constandse-Westerman, T. S. (1978). The human skeletal material from swifterbant, Earlier Neolithic of the northern Netherlands: I. Inventory and demography. *Palaeohistoria*, 39–89.

Dreshaj, M., D.C.M. Raemaekers & M. Dee, (2024). Chronological modelling on a calibration plateau: implications for the emergence of agriculture in the Dutch wetlands, *Radiocarbon* 65(6), 1280-1298. https://doi.org/10.1017/RDC.2023.126

Huisman, D.J. & D.C.M. Raemaekers, (2014). Systematic cultivation of the Swifterbant wetlands (The Netherlands). Evidence from Neolithic tillage marks (c. 4300–4000 cal. BC), *Journal of Archaeological Science* 49, 572-584.

Kubiak-Martens, L. & Van der Linden, M. (eds) (2022). *Neolithic Human Diet. Based on Studies of Coprolites from the Swifterbant Culture Sites, the Netherlands*. Nederlandse Archeologische Rapporten 77. Amersfoort: Cultural Heritage Agency of the Netherlands.

Raemaekers, D.C.M., J. Geuverink, I. Woltinge, J. van der Laan, A. Maurer, E.E. Scheele, T. Sibma & D.J. Huisman, (2014). Swifterbant-S25 (gemeente Dronten, provincie Flevoland). Een bijzondere vindplaats van de Swifterbant-cultuur (ca. 4500-3700 cal. BC), *Palaeohistoria* 55/56, 1-56.

Schepers, M. & N. Bottema-Mac Gillavry, (2020). The vegetation and the exploitation of plant resources. In D.C.M. Raemaekers & J.P. de Roever (red.), *Swifterbant S4 (the Netherlands). Occupation and exploitation of a Neolithic levee site (c. 4300-4000 cal. BC),* Groningen (Groningen Archaeological Studies 36),51-75.

Van Zeist, W. , & R.M. Palfenier-Vegter, (1981). Seeds and fruits from the Swifterbant S3 site. Final Reports on Swifterbant IV, *Palaeohistoria* 23, 105-168.

## 2.14 Angeren-Kampsepad Zuid (Gelderland, the Netherlands)

**Analyzed individual:**

I38442 (Grave 2, S454)

**Contact information**: Judith van der Leije, Constance van der Linde

**Site information and excavation history**: The site Angeren-Kampsepad Zuid (municipality of Lingewaard, Gelderland) was excavated in advance of an extension of the A15 motorway, in 2022. The site was located on the outer bank of a channel that began to silt up around 5200 BCE. Three archaeological levels were stratigraphically separated from one another. The lower one (Level 3) contained archaeological finds from the Early Neolithic, finds and features from the Middle Neolithic A were associated with the middle level (Level 2). The upper level (Level 1) was used in the Middle Neolithic B and Late Neolithic. Of these three levels, Level 2 is of particular importance in this context. Based on radiocarbon dating of seeds and charcoal from the channel's infill, this level has been dated to between approximately 4600 and 3400 cal BCE. The find material includes Swifterbant and Hazendonk ceramics. Beneath the find layer, features such as pits, postholes, and inhumation graves were identified.

The excavation yielded four inhumation graves. In addition, a separate skull had been deposited in a pit and loose human skeletal elements were found within the find layer, bringing the minimum number of individuals to six.

**Summary of the sampled materials:** Samples for DNA analysis were taken from the petrous bones from each of the four buried individuals as well as from the separately buried skull. Only one sample yielded results. The sample, I38442, was from a boy of 5 years +/- 16 months old. The child was buried in a strongly flexed position, lying on his left side. Next to the skeleton, in the grave pit, a base of a red deer antler was found, which might be considered as an intentional grave good.

**Dating**: Unfortunately, radiocarbon dating didn’t yield any results because there was not enough collagen preserved in the bones. Neither were there any datable grave goods. A sample of the antler was also sent in for ^14^C-analysis but didn’t yield any result. Based on its stratigraphic position, the grave can be roughly dated to between 4600 and 3400 BCE. This is consistent with the Swifterbant and Hazendonk ceramics on the site. Based on the position of the body on the side and the strongly flexed limbs (comparable to the individuals of the Hazendonk sites Ypenburg-Locatie 4 (Baetsen 2008) and Schipluiden-Harnaschpolder (Smits 2006), an attribution to the Hazendonk culture (3800-3400 BCE) seems most likely, but an older allocation to the Swifterbant cannot be excluded. In light of the high WHG ancestry found for this individual (84%), we take a conservative approach and attribute this individual to the Swifterbant culture in this study.

**Isotopes:** A molar of the individual was sampled for isotope analysis. Strontium as well as oxygen isotope analyses was performed. The data are presented in table SI2.18. The ^87^Sr/^86^Sr ratio is compatible with the direct surroundings of Angeren-Kampsepad. The δ^18^O_VPDB_ however, is more negative than would be expected for the region of Angeren-Kampsepad. It places the individual outside the current national borders of the Netherlands. Such negative values occur in higher-altitude regions of Europe, such as the Alps, as well as in areas located farther inland, such as large parts of Eastern Europe, Central Europe or parts of Scandinavia. This would mean that the child had already travelled a considerable distance at a very young age, brought along by his parents and/or community.

### ***Table SI2.17. Sr and O isotope data for grave 2 from Angeren-Kampsepad Zuid***.

***The element numbers are according to Federation Dentaire Internationale (FDI).***

| **Sample ID** | **Material** | **Element** | **^87^Sr/^86^Sr** | **2SE** | ***δ*^13^C_PDB_ (‰)** | **SD** |
| --- | --- | --- | --- | --- | --- | --- |
| Grave 2 | Enamel | 16 | 0,709046 | 0.000006 | -8,17 | 0.24 |

**Source of the samples**: ADC Archeoprojecten/Archol bv/BAAC BV. Samples collected by Constance van der Linde

**Author of the entry:** Judith van der Leije, Constance van der Linde, Lisette Kootker

**References**: The site is due to be published in 2026.

Baetsen, S. 2008. Het grafveld, in J.M. Koot, L. Bruning & R.A. Houkes (ed.) *Ypenburg-locatie 4: een nederzetting met grafveld uit het midden neolithicum in het west-Nederlandse kustgebied*: 119–88. Leiden: Hazenberg Archeologie.

Smits, E. & L.P. Louwe Kooijmans. 2006. 5 Graves and human remains, in L.P. Louwe Kooijmans & P.F.B. Jongste (ed.) *Schipluiden: A Neolithic Settlement on the Dutch North Sea Coast c. 3500 cal BC*: 91–112 (Analecta Praehistorica Leidensia 37/38). Leiden: Leiden University Press.

## *Belgium*

## 2.15 Abri des Autours (Dinant, Namur, Belgium)

### Table SI2.18 samples used from Abri des Autours individuals

| **ID number** | **Sample material** | **Lab code** | ^14^C Age BP ± 1 σ | **95.4% cal BCE** | **δ**^13^C |
| --- | --- | --- | --- | --- | --- |
| AAT001 (AA3) | collagen bone | OxA-4917 | 9500 ± 75 | 9160-8623 | -20.2 |

A detailed archaeological context was published in: Semal, P., Polet, N., & Cauwe, N. (2023). Abri de Autours, Belgium  Supplementary information; section 1 archaeological contact information to Posth et al. 2023. *Nature*, *615*(7950). https://doi.org/10.1038/s41586-023-05726-0

## 2.16 Malonne-Petit Ri (Namur, Belgium)

### Table SI2.19 samples used from Malonne Petit Ri individuals

| **ID number** | **Sample material** | **Lab code** | ^14^C Age BP ± 1 σ | **95.4% cal BCE** | **δ**^13^C |
| --- | --- | --- | --- | --- | --- |
| MPR001 (MPR-1) | collagen bone | OxA-5042 | 9270 ± 90 | 8731-9294 | - |

A detailed archaeological context was published in: Semal, P., & Jadin, I. (2023). Malonne Petit Ri, Belgium  Supplementary information; section 1 archaeological contact information to Posth et al. 2023. *Nature*, *615*(7950). https://doi.org/10.1038/s41586-023-05726-0

## 2.17 Trou Al’Wesse (Modave, Liège, Belgium)

**Analyzed sample:**

| I13627 | (1 / x4; AF004) |  |
| --- | --- | --- |

I13642 (2 / 3120; AF018)

I13648 (4 / 3262; AF024)

**Contact information:** Maria Pala, John Stewart

**Site information and excavation history:**

Trou Al’Wesse (‘Wasp Cave’ in the Walloon dialect) is part of a limestone karstic cave system located in the Hoyoux valley. The cave extends almost horizontally for 35m inside a cliff. At the back, the ceiling opens through a 9m long chimney that connects to the plateau above. The cave has been known since excavations first occurred in the 1860s, and it has been the object of numerous investigations since (Masy, 2020-2022, Miller et al., 2011, Flas et al., 2019). The Pleistocene and Holocene deposits show different phases of human occupation from the Middle and Upper Palaeolithic, Mesolithic and Neolithic.

**Summary of the sampled materials:** The human remains included in this study were retrieved from the deposits filling the chimney at the back of the cave, and were excavated in the 1880s (Masy, 1993)**.**

**Dating:**

### Table SI2.20 ^14^C dates from Trou Al’Wesse

| **ID number** | **Sample material** | **Lab code** | ^14^C Age BP ± 1 σ | **95.4% cal BCE** | **δ**^13^C |
| --- | --- | --- | --- | --- | --- |
| I13627 (1 / x4; AF004) | Collagen bone, left petrous | OxA-39060 | 4466 ± 21 | 3333-3026 | -20.34 |
|  | 5th left metatarsal | Beta-319269 | 4560 ± 30 | 3488-3103 |  |
|  | 5th left metatarsal | Beta-319270 | 4450 ± 30 | 3336-2937 |  |

Previous radiocarbon dates on two human 5^th^ left metatarsals placed these burials in the second half of the 4^th^ millennium BCE (Miller *et al*., 2012; recalibrated in Oxcal 4.4).

Genetic Identifier: I13627. Grave Identifier: 1 / x4; AF004. Grave type: chimney burial. Skeletal information: left petrous. Grave goods: none.

The following four samples were not used in the genetic analysis because of suspected DNA contamination (Supplementary Table 2):

Genetic Identifier: I13642. Grave Identifier: 2 / 3120; AF018. Grave type: chimney burial. Skeletal information: left humerus. Grave goods: none. Dating: context date 3500-3100 BCE.

Genetic Identifier: I13648. Grave Identifier: 4 / 3262; AF024. Grave type: chimney burial. Skeletal information: right tibia. Grave goods: none. Dating: context date 3500-3100 BCE.

Genetic Identifier: I13643. Grave Identifier: 4 / 3262; AF019. Dating: context date 3500-3100 BCE.

Genetic Identifier: I13645. Grave Identifier: AF021. Dating: context date 3500-3100 BCE.

**Source of the samples:** University of Huddersfield

**Authors of the entry:** Maria Pala, Alessandro Fichera

**References**:

Flas D., Zwyns N., Stewart J., Wilkinson K., Barrett N., Knul M. & Noiret P. (2019). Modave/Modave: Trou Al'Wesse, fouilles 2018. *Chronique de l’Archaeologie Wallonne* 27, 161-164.

Masy P. (1993). La sépulture collective néolithique du trou Al’Wesse à Modave (province de Liège). *Bulletin des Chercheurs de la Wallonie***,** XXXIII, 81-99.

Masy P. (2020-2022). Historique du Trou Al’Wesse (Modave) avant les fouilles modernes commencées en 1988. *Bulletin des Chercheurs de la Wallonie* 55**,** 5-23.

Miller R., Collin F., Otte M. & Stewart J. (2011). Le Trou Al’Wesse: du Moustérien au Néolithique dans la vallée du Hoyoux. *Le Paléolithique moyen en Belgique. Mélanges Marguerite Ulrix-Closset.* Liège: ERAUL 128.

Miller R., Stassart E., Otte M., Austin P. & Stewart J. (2012). Interprétation chronostratigraphique de la séquence holocène du Trou Al'Wesse à la lumière des nouvelles datations: du Mésolithique ancien au Néolithique moyen (Modave, B). *Notae Praehistoricae* 32**,** 133-139.

## 2.18 Grotte du Mont Falise (Liège, Belgium)

**Analyzed samples:**

| I13629 | (213 / 3259); AF006 |
| --- | --- |
| I13631 | (213 / 15.019); AF008 |
| I13638 | (213 / 3252); AF014 |
| I13649 | (212 / 1x.021); AF026 |
| I13651 | (212 / 3245); AF028 |
| I13630 | (213 / 3274); AF007 |
| I13653 | (213 / 3251); AF030 |

**Contact information:** Maria Pala, Damien Flas, Pierre Noiret

**Site information and excavation history:**

The Grotte du Mont Falise, is a cave located near Antheit (Liège province). It opens into a limestone cliff in the basin of the Mehaigne. The cave was excavated in the 1890s by Julien Fraipont, and in 1958 by Haeck (Haeck 1964, Fraipont 1897). Apart from human remains, the site yielded archaeological material from the Middle and Upper Palaeolithic, Neolithic, Iron Age, Roman, and Medieval periods (Haeck 1964). Little contextual information is available on this site as the documentation on both the 19^th^ century and mid-20^th^ century excavation is very limited. The skeletal remains are thought to originate from a collective burial dating to the Neolithic. The available radiocarbon dates, both directly on the sampled materials as well as two other available radiocarbon dates appear to confirm this.

**Summary of the sampled materials:**

Genetic Identifier: I13629. Grave Identifier: 213 / 3259; AF006. Grave type: collective grave. Skeletal information: juvenile (12 years); molar (LLM2). Grave goods: none. Dating: context date 2950-2600 BCE (PSUAMS generated no collagen; skeletal element: left mandibular condyle).

Genetic Identifier: I13631. Grave Identifier: 213 / 15.019; AF008. Grave type: collective grave. Skeletal information: left femur. Grave goods: none. Dating: context date 2950-2600 BCE.

Genetic Identifier: I13638. Grave Identifier: 213 / 3252; AF014. Grave type: collective grave. Skeletal information: molar. Grave goods: none. Radiocarbon dated to 2886-2668 cal BCE.

Genetic Identifier: I13649. Grave Identifier: 212 / 1x.021; AF026. Grave type: collective grave. Skeletal information: femur. Grave goods: none. Dating: context date 3050-2600 BCE.

Genetic Identifier: I13651. Grave Identifier: 212 / 3245; AF028. Grave type: collective grave. Skeletal information: femur. Grave goods: none. Dating: context date 3050-2600 BCE.

**Dating:**

### Table SI2.21 ^14^C dates from Grotte du Mont Falise

| **ID number** | **Sample material** | **Lab code** | ^14^C Age BP ± 1 σ | **95.4% cal BCE** | **δ**^13^C |
| --- | --- | --- | --- | --- | --- |
| I13638 (213 / 3252; AF014) | apatite tooth | PSUAMS-12142 | 4180 ± 25 | 2886-2668 | -21.62 |
| I13630 (213 / 3274; AF007) | molar | OxA-39062 | 4174 ± 21 | 2882-2669 | - |
|  | right ulna | OxA-10687 | 4195 ± 40 | 2897-2632 |  |
|  | right ulna | OxA-10688 | 4265 ± 40 | 3010-2699 |  |

I13638 and I13630 have been radiocarbon dated directly and can both be placed between ca. 2885-2670. This is corroborated by two other radiocarbon dates available on two right ulnas from the same collective burial (Toussaint 2003).

**Source of the samples:** University of Huddersfield

**Authors of the entry:** Maria Pala, Alessandro Fichera, Damien Flas

**References**:

Fraipont, J. (1897). La grotte du mont Falhise [Anthée]. *Bulletins de l'Académie royale des sciences, des lettres et des beaux-arts de Belgique.* Bruxelles: Académie royale des sciences, des lettres et des beaux-arts de Belgique.

Haeck, J. (1964). La grotte du Mont Falise à Antheit, vallée de la Méhaigne, province de Liège. *Bulletin de la Société royale belge d'Anthropologie et de Préhistoire* 74, 39-54*.*

Toussaint, M. (2003). Wanze/Antheit: apport des datations radiocarbones d'ossements humains de la grotte du Mont Falise à la problématique des sépultures protohistoriques en milieu karstique. *Chronique de l’Archéologie Wallonne* 11, 99-101*.*

## 2.19 Abri Sandron (Liège, Belgium)

**Analyzed samples:**

| I13633 | (97; AF010) |
| --- | --- |
| I13635 | (89 / 6763; AF012) |
| I13654 | (X1; AF031) |
| I13655 | (88 / 6165; AF032) |
| I13656 | (90; AF033) |
| I13657 | (91; AF034) |
| I13659 | (94; AF036) |
| I13660 | (95; AF037) |

**Contact information:** Maria Pala, Damien Flas, Pierre Noiret **Site information and excavation history:**

Abri Sandron is a rock-shelter located in the Mehaigne valley, near Huccorgne (Liege Province). The site has been excavated several times between the 1880s and 1960s. The main excavations were organised by Julien Fraipont and Fernand Tihon in 1887 and 1888 (Fraipoint 1898) and yielded the human remains sampled here. Besides hundreds of bones, corresponding to at least 15 individuals (Toussaint, 2002, Fraipont, 1898), the archaeological material includes Pleistocene (Middle and Upper Palaeolithic; (Otte, 1979) and Holocene (mostly Neolithic) fauna and artefacts.

**Summary of the sampled materials:** The human remains included in this study were part of a collection excavated in 1887 and 1888 by Julien Fraipont and Fernand Tihon (Fraipoint 1898).

Genetic Identifier: I13633. Grave Identifier: 97; AF010. Grave type: collective grave. Skeletal information: molar (LRM3). Grave goods: none.

Genetic Identifier: I13635. Grave Identifier: 89 / 6763; AF012. Grave type: collective grave. Skeletal information: adult; left petrous. Grave goods: none.

Genetic Identifier: I13654. Grave Identifier: X1; AF031. Grave type: collective grave. Skeletal information: molar (M1). Grave goods: none.

Genetic Identifier: I13655. Grave Identifier: 88 / 6165; AF032. Grave type: collective grave. Skeletal information: molar (RM3). Grave goods: none.

Genetic Identifier: I13656. Grave Identifier: 90; AF033. Grave type: collective grave. Skeletal information: molar (LLM3). Grave goods: none.

Genetic Identifier: I13657. Grave Identifier: 91; AF034. Grave type: collective grave. Skeletal information: juvenile; molar (ULM3). Grave goods: none.

Genetic Identifier: I13659. Grave Identifier: 94; AF036. Grave type: Collective grave. Skeletal information: none. Grave goods: molar (LLM).

Genetic Identifier: I13660. Grave Identifier: 95; AF037. Grave type: collective grave. Skeletal information: juvenile; molar (LLM2). Grave goods: none.

**Dating:**

### Table SI2.22 ^14^C dates from Abri Sandron

| **ID number** | **Sample material** | **Lab code** | ^14^C Age BP ± 1 σ | **95.4% cal BCE** | **δ**^13^C |
| --- | --- | --- | --- | --- | --- |
| I13633 (97; AF010) | mandible | PSUAMS-12141 | 4260 ± 25 | 2916-2781 | -21.62 |
| I13635 (89 / 6763; AF012) | left petrous | PSUAMS-11920 | 4035 ± 30 | 2660-2468 | -20.90 |
| I13657 (91; AF034) | maxilla | PSUAMS-12143 | 4265 ± 25 | 2917-2786 | -21.00 |
| I13659 (94; AF036) | molar | PSUAMS-12144 | 4245 ± 25 | 2911-2706 | -21.09 |
| I13660 (95; AF037) | mandible | UBA-42622 | 4103 ± 29 | 2865-2502 | -20.60- |
|  | Human bone, 2nd right metacarpal | OxA-10555 | 4235 ± 45 | 2921-2638 | - |
|  | Human bone, 2nd right metacarpal | OxA-10556 | 4183 ± 38 | 2891-2631 | - |
|  | Human bone, 2nd right metacarpal | OxA-10557 | 4280 ± 40 | 3016-2707 | - |

Five radiocarbon dates were obtained directly on the sequenced individuals, all falling within a fairly tight range between ca. 2900-2700 cal BCE with perhaps a few burials slightly later (I13635 and I13660) ca. 2660-2468 cal BCE. This is supported by three additional dates on three 2^nd^ right metacarpals, all confirming a Late neolithic chronology (Toussaint, 2002).

**Source of the samples:**

**Authors of the entry:** Maria Pala, Alessandro Fichera, Damien Flas

**References**:

Fraipont, J. (1898). Les Néolithiques de la Meuse. Type de Furfooz. *Bulletin de la Société d’Anthropologie de Bruxelles.* Bruxelles.

Otte, M. (1979). *Le paléolithique supérieur ancien en Belgique*, Bruxelles : Musées royaux d'art et d'histoire.

Toussaint, M. (2002). Problématique chronologique des sépultures du Mésolithique mosan en milieu karstique. *Notae Praehistoricae,* 22**,** 141-166.

## 2.20 Pommerœul (Hainault, Belgium)

### Table SI2.23 samples used from Pommereuil individuals

| **ID number** | **Sample material** | **Lab code** | ^14^C Age BP ± 1 σ | **95.4% cal BCE** | **δ**^13^C |
| --- | --- | --- | --- | --- | --- |
| I18068 (T26-C) | - | RICH-27887 | 4320 ± 27 | 3011-2890 | -20,90 |
| I21570 (T26-J) | - | RICH-27891 | 4278 ± 27 | 2926-2789 | - |

These samples have been reported in detail by Veselka, B. et al. 2024. Assembling Ancestors: the manipulation of Neolithic and Gallo-Roman skeletal remains from Pommeroeul, Belgium. *Antiquity online*, 1-16.

## 2.21 Grotte de la faille du Burin (Namur, Belgium)

**Analyzed samples:**

| I7010 | (BELG_265) |
| --- | --- |

**Contact person:** Michel Toussaint

**Site information and excavation history:** The Faille du Burin is a small cave at the base of the rocks surmounted by the medieval castle of Samson, just above the slopes that descend towards the Samson river, a tributary of the Meuse river. The cavity opens to the northeast and appears as a narrow crack that extends southwest for nearly 8 m before opening into a small room of about 1.50 m^2^, where the human bones were found by P. Lacroix who excavated dozens of cranial and postcranial bones (Toussaint & Lacroix, 2002). The bones correspond to at least four adults and two children. No archaeological material was collected in direct contact with the bones; a flint blade was discovered in the access corridor. It seems however that, during previous speleologist work, the anterior part of the cavity has also yielded human bones dating back to the Neolithic.

**Summary of the sampled materials:**The human bone analyzed in this article comes from the small room located about ten meters from the entrance of the cave. The sampled individual in this study is I7010.

**Dating:**

### Table SI2.24 ^14^C dates from the Grotte de la faille du Burin

| **Site** | **Sample material** | **Lab code** | ^14^C Age BP ± 1 σ | **95.4% cal BCE** | **δ**^13^C |
| --- | --- | --- | --- | --- | --- |
| ***Grotte de la faille du Burin*** | collagen left navicular | OxA-10585 | 9520 ± 55 | 9140-8645 | -19.6 |
|  | collagen left navicular | OxA-8938 | 9345 ± 75 | 8800-8340 | -19.6 |
|  | collagen left navicular | OxA-10595 | 9335 ± 65 | 8765-8345 | -19.6 |
|  | collagen left navicular | OxA-10564 | 9315 ± 50 | 8710-8350 | -20.0 |

The sequenced sample has not been directly dated. However, four AMS radiocarbon dates were obtained from four left navicular bones from four different subjects found in the small room at the back of the cavity. These radiocarbon dates are consistent with the already rich corpus of radiocarbon results obtained from early Mesolithic burials of our regions (Toussaint, 2010 ; Meiklejohn *et* *al.*, 2014).

**Source of the samples:** The material discovered at the “Faille du Burin” is in the process of being deposited in public collections.

**Authors of the entry:** Michel Toussaint

**References**:

Meiklejohn, C., Miller, B., & Toussaint, M. (2014). Radiocarbon dating of Mesolithic human remains in Belgium and Luxembourg. *Mesolithic Miscellany* 22, 10–39.

Toussaint M. (2010). Les sépultures mésolithiques du bassin mosan wallon : où en est la recherche en 2010 ? *Bulletin des Chercheurs de la Wallonie*, hors-série n° 2, 69-86.

Toussaint M. & Lacroix Ph. (2002). Andenne/Thon : la Faille du Burin à Samson, une nouvelle sépulture collective du Mésolithique ancien. *Chronique de l'Archéologie wallonne*, 10/2002, 228-230.

## 2.22 Grotte Rousseau (Lustin, Profondeville, Namur, Belgium)

**Analyzed samples:**

| I7012 | (BELG_267) |  |
| --- | --- | --- |
| I7018 | (BELG_7764) |  |

**Contact person:** Michel Toussaint

**Site information and excavation history:** This small cave was discovered during speleological and archaeological surveys by Philippe Lacroix in the 1980s and 1990s, near the Meuse river. It has not been the subject of detailed excavations, but only small surveys. The most spectacular piece collected is a part of a right human foot with bone ankylosis (Masy, 1997).

**Summary of the sampled materials:** The human bones analyzed in this article were found near the entrance of the cave. Two individuals have been sequenced and have been directly radiocarbon dated. Interestingly one dates to the Mesolithic (I7018), the other to the Neolithic (I7012).

**Dating**:

### Table SI2.25 ^14^C dates from the Grotte Rousseau

| **ID number** | **Sample material** | **Lab code** | ^14^C Age BP ± 1 σ | **95.4% cal BCE** | **δ**^13^C |
| --- | --- | --- | --- | --- | --- |
| I7012 (BELG_267) | bone | PSUAMS-7872 | 4020 ± 25 | 2618-2468 | -20.77 |
| I7018 (BELG_7764) | bone | PSUAMS-7873 | 9190 ± 45 | 8547-8293 | -20.15 |
| - | Human bone, adult 5th metatarsal | OXA-8877 | 4270 ± 70 | 3091-2630 | - |
| - | Human bone, adult 5th metatarsal | OxA-8809 | 4150 ± 50 | 2882-2581 | - |

Two AMS radiocarbon dates were obtained from fifth metatarsals from two different individuals found near the entrance of the cavity (Toussaint *et al.*, 2020). The two sequenced individuals were also dated directly on the human bone. While three radiocarbon dates suggest the origin of the skeletons to be in the early 3^rd^ millennium BCE, I7018 must be dated in the Early Mesolithic. This is confirmed by the genetic profile of this individual.

**Source of the samples:** The anthropological material discovered at the Rousseau cave are in the process of being deposited in public collections.

Authors of the entry: Michel Toussaint

**References** :

Masy Ph. (1997). Notice, in descriptive catalogue in *Le secret des dolmen*s. Wéris, Musée des Mégalithes, 145.

Toussaint M., Smolderen A., Bocherens H., Cattelain L., Collin J.-Ph. & Cattelain P. (2020). La Grotte Ambre à Matagne-la-Grande (Doische, Namur, Belgique) : étude anthropologique, biogéochimique et archéologique d’un amas d’ossements humains du Néolithique final du bassin mosan wallon. *Archeo-Situla* 39, 63-100.

## 2.23 Wéris (Durbuy, Luxembourg, Belgium)

**Analyzed samples:**

| I7014 | (BELG_273) |  |
| --- | --- | --- |

**Contact person:** Michel Toussaint

**Site information and excavation history:** The megalithic complex of Wéris (Durbuy, province of Luxembourg) stretches over some 8 km long and 300 m wide, on two plateaus aligned on either side of the Aisne, an Ardennes tributary on the right bank of the Ourthe river. The sites consist of two passage graves (Wéris I and Wéris II) with associated standing stones, and six sites composed of one or more menhirs. All these monuments are made of puddingstone, a rock that has formed in natural benches on the Ardennes ridge overlooking the Wéris plateau (Toussaint, 2003; Toussaint *et al*., 2009).

The interest of the Wéris megalithic field has long been recognised. The two passage graves and the site of the menhirs of Oppagne were the subject of initial excavations at the end of the 19th century and the beginning of the 20th century. Recent excavations at the end of the 20th century and the beginning of the 21st century were still carried out in the two passage graves but also in a series of sites with standing stones discovered on this occasion

The two passage graves are built according to the same rectangular plan. They both have an ante-chamber, an elongated burial chamber and a posterior slab lying behind the back stone. It is in these two alleys, clearly formerly disturbed, that very rare archaeological and anthropological material was discovered both during the first excavations and recent excavations.

**Summary of the sampled materials:** The human bone analyzed in this article comes from the Wéris II covered alley (I7014).

**Dating**: Five ^14^C dates were obtained from the Wéris megalithic field, all using human bones (Toussaint 2003, Toussaint *et al*. 2009):

### Table SI2.26 ^14^C dates from the sites at Wéris

| **site** | **Sample material** | **Lab code** | ^14^C Age BP ± 1 σ | **cal. BCE (2σ)** | **δ^13^C** |
| --- | --- | --- | --- | --- | --- |
| ***Wéris I :*** | Adult phalange | OxA-6457 | 4240 ± 65 | 3010-2625 | **-** |
|  | Fragment of maxilla | OxA-6458 | 4170 ± 60 | 2895-2580 | **-** |
| ***Wéris II* :** | Adult second left metacarpal | OxA-8956 | 4240 ± 45 | 2925-2640 | -20.40 |
|  | Adult five left metatarsal | OxA-8939 | 4180 ± 40 | 2890-2630 | -20.50 |
| I7014 | bone | PSUAMS-14124 | 4055 ± 20 | 2831-2476 | -20.61 |
| ***Heyd standing stone* :** | Human clavicle | OxA-8828 | 4425 ± 45 | 3330-2920 | -20.90 |

**Source of the samples:** The archaeological and anthropological material discovered in Wéris is very poor due to old alterations of the two gallery graves (*allées couvertes*). The few discoveries from the first excavations, at the end of the 19th century and the beginning of the 20th century, are kept at the Archaeological Museum in Arlon. The archives and the few objects from the recent excavations are kept in Namur at the “Agence wallonne du Patrimoine, Service public de Wallonie.

**Authors of the entry:** Michel Toussaint

**References :**

Toussaint M. (ed.), (2003). Le « champ mégalithique de Wéris ». Fouilles de 1979 à 2001. Volume 1. Contexte archéologique et géologique, Namur,  Études et Documents, Archéologie, 9.

Toussaint M. Frébutte C. & hubert F. (eds.), (2009). *Le « champ mégalithique de Wéris ». Fouilles de 1979 à 2001, volume 2. Rapports de fouilles*. Namur, Études et Documents, Archéologie 15.

## 2.24 Claminforge (Sambreville, Namur, Belgium)

**Sample analyzed**:

| I7015 | (BELG_6598) |
| --- | --- |

**Contact person:** Michel Toussaint

**Site information and excavation history:** Claminforge is a small cave site on the left bank of the Bième, a tributary of the Sambre. The site was first discovered in 1988 by a group of speleologists of the Centre Spéléologique de la Basse Sambre(Meiklejohn et al. 2014). In 1995 a small rescue excavation was carried out by M. Toussaint (Toussaint 2019). The front part of the site was formerly destroyed by the activity of a limestone quarry. Therefore the human remains discovered represent only part of the original bone deposit. All of them, often fragmentary, have been discovered at the end narrow gallery of barely two square meters, most of them by speleologists in 1988. Seven individuals have been recovered from the cave. Direct dating of two bones, carried out just after the 1995 excavation, corresponds to the early Mesolithic. However, new, as yet unpublished datings carried out recently at Ghent University show that some of the other individuals correspond to the Neolithic. This discovery could possibly be interpreted as two successive secondary burials, one Mesolithic and one Neolithic, but more probably as rejections of bones intended to make room for new corpses.

**Summary of the sampled materials:** The present assemblage gives evidence for the presence of at least seven individuals: four adults and three children aged between 8 and 11 years (Toussaint 2019). One of these individuals yielded sample I7015.

**Dating:**

### Table SI2.27 ^14^C dates from Claminforge

| **site** | **Sample material** | **Lab code** | ^14^C Age BP ± 1 σ | **95.4% cal BCE** | **δ^13^C** |
| --- | --- | --- | --- | --- | --- |
| ***Claminforge*** | Cervical vertebra | OxA-5451 | 9320 ± 75 | 8755-8315 | -19,40 |
|  | 3rd metacarpal | OxA-10552 | 9525 ± 60 | 9156-8642 | -19,40 |

Two of the individuals have been dated: after the rescue excavation in 1995 Toussaint discovered, a human third metacarpal, which yielded a first ^14^C date; a cervical vertebra found a year later, yielded a second date (Toussaint 2019, 255).

**Source of the samples:** The bones of Claminforge are preserved at the Prehistomuseum of Ramioul, in Flémalle, in the province of Liège, Belgium.

**Authors of the entry:** Michel Toussaint

**References :**

Meiklejohn, C., Miller, B., & Toussaint, M. (2014). Radiocarbon dating of Mesolithic human remains in Belgium and Luxembourg. *Mesolithic Miscellany* 22, p.10–39.

Toussaint, M. (2019). Les ossements humains du Mésolithique ancien de la grotte de Claminforge (Sambreville, province de Namur, Belgique). *Bulletin des Chercheurs de la Wallonie, Tome LIV*, 251–281.

## *Germany*

## 2.25 Hagen-Blätterhöhle cave (Westphalia, Germany)

### Table SI2.28 samples used from Blätterhöhle individuals

| **ID number** | **Sample material** | **Lab code** | ^14^C Age BP ± 1 σ | **cal. BCE (2σ)** | **δ^13^C** |
| --- | --- | --- | --- | --- | --- |
| I1563 Bla5+Bla7+Bal13+Bla26(o)+Bla30+Bla54 (Excavation 2004, 2014) | - | [R_combine: (4580±30, KIA-28844, Bla5); (4860±30, KIA-45011, Bla7); (4730±25, KIA-45010, Bla13)] | 4726 ± 17 | 3626-3378 | - |
| I1565 Bla8+Bla9+Bla11+Bla24+Bla26(x)+Bla45 (Excavation 2004, 2014) | - | [R_combine: (4950±30, KIA-45006, Bla8); (4905±25, KIA-45008, Bla9); (5145±30, KIA-45007, Bla11); (4845±35, KIA-37507, Bla24)] | 4965 ± 15 | 3783-3655 | - |
| I1593 Bla16+Bla27+Bla59 (Excavation 2004, 2014) | - | [R_combine: (4615±30, KIA-28845, Bla16); (5055±35, KIA-37508, Bla27)] | 4810 ± 23 | 3644-3528 | - |
| I1594 Bla28 (Excavation 2004) | - | KIA-28846 | 4465±30 | 3338-3024 | - |

A detailed description is published by Bollongino, R., O. Nehlich, M.P. Richards, J. Orschiedt, M.G. Thomas, C. Sell, Z. Fajkosová, A. Powell & J. Burger. 2013. 2000 years of parallel societies in Stone Age Central Europe. *Science (New York, N.Y.)* 342: 479–81. https://doi.org/10.1126/science.1245049.

A detailed analysis is published by Lipson, M., Szécsényi-Nagy, A., Mallick, S., Pósa, A., Stégmár, B., Keerl, V., Rohland, N., Stewardson, K., Ferry, M., Michel, M., Oppenheimer, J., Broomandkhoshbacht, N., Harney, E., Nordenfelt, S., Llamas, B., Gusztáv Mende, B., Köhler, K., Oross, K., Bondár, M., … Reich, D. (2017). Parallel palaeogenomic transects reveal complex genetic history of early European farmers. *Nature*, *551*(7680), 368–372. https://doi.org/10.1038/nature24476

## 2.26 Niedertiefenbach (Germany)

### Table SI2.29 samples used from Niedertiefenbach individuals

| **ID number** | **Sample material** | **Lab code** | ^14^C Age BP ± 1 σ | **cal. BCE (2σ)** | **δ^13^C** |
| --- | --- | --- | --- | --- | --- |
| KH150189_KH150632_KH150636 (NT1) | - | KIA-53052 | 4493 ± 26 | 3346-3095 | - |
| KH150190 (NT21) | - |  |  |  | - |
| KH150191 (NT39) | - |  |  |  | - |
| KH150193_KH150286 (NT9A+ 9) | - |  |  |  | - |
| KH150195_KH150196_KH150616 (NT23/123/128b) | - | KIA-52274 | 4492 ± 24 | 3342-3096 | - |
| KH150197 (NT122.1) | - |  |  |  | - |
| KH150198 (NT186) | - |  |  |  | - |
| KH150200 (NT136.2) | - |  |  |  | - |
| KH150203 (NT121) | - | KIA-52267 | 4481 ± 24 | 3339-3036 | - |
| KH150204_KH150634 (KH150204_KH150634) | - | KIA-53051 | 4507 ± 25 | 3352-3099 | - |
| KH150208_KH150210 (NT26 /111) | - |  |  |  | - |
| KH150287 (NT54) | - | KIA-52268 | 4499 ± 25 | 3346-3098 | - |
| KH150289 (NT98) | - | KIA-52270 | 4499 ± 24 | 3346-3098 | - |
| KH150418 (NT41+45+43) | - |  |  |  | - |
| KH150419 (NT27) | - |  |  |  | - |
| KH150422 (NT46) | - | KIA-52272 | 4538 ± 24 | 3366-3103 | - |
| KH150610 (NT17) | - | KIA-52273 | 4532 ± 25 | 3365-3102 | - |
| KH150612 (NT5+6.2) | - |  |  |  | - |
| KH150613_KH180043 (NT107) | - | KIA-53045 | 4499 ± 26 | 3346-3098 | - |
| KH150614_KH150615 (NT142.1) | - | KIA-53046 | 4462 ± 24 | 3334-3025 | - |
| KH150618 (NT48) | - | KIA-52275 | 4468 ± 25 | 3336-3028 | - |
| KH150619 (NT42) | - | KIA-52276 | 4455 ± 2 | 3333-3021 | - |
| KH150620 (NT148) | - | KIA-53047 | 4491 ± 25 | 3344-3094 | - |
| KH150621 (NT50) | - |  |  |  | - |
| KH150622 (NT130) | - | KIA-53048 | 4417 ± 19 | 3264-2926 | - |
| KH150623 (NT135) | - |  |  |  | - |
| KH150625 (NT83) | - | KIA-52277 | 4497 ± 27 | 3348-3096 | - |
| KH150626 (NT58) | - |  |  |  | - |
| KH150627 (KI11) | - | KIA-52278 | 4462 ± 27 | 3335-3024 | - |
| KH150628 (NT150.1) | - | KIA-53049 | 4448 ± 26 | 3334-2938 | - |
| KH150629 (NT30) | - |  |  |  | - |
| KH150630 (KI12) | - | KIA-53050) | 4564 ± 25 | 3487-3106 | - |
| KH150633 (KI13) | - | KIA-52279) | 4486 ± 29 | 3344-3036 | - |
| KH150635 (KI14) | - | KIA-52280) | 4425 ± 28 | 3322-2924 | - |
| KH150637 (NT110) | - | KIA-52281) | 4432 ± 28 | 3328-2926 | - |
| KH150639 (NT49) | - |  |  |  | - |
| KH150640 (NT98) | - | KIA-53053) | 4461 ± 25 | 3334-3024 | - |
| KH150641 (KI15) | - | KIA-52282) | 4473 ± 28 | 3339-3028 | - |
| KH180044 (NT136.1) | - |  |  |  | - |
| KH180045 (NT146) | - |  |  |  | - |

A detailed analysis was published in: Immel, A., Pierini, F., Rinne, C., Meadows, J., Barquera, R., Szolek, A., Susat, J., Böhme, L., Dose, J., Bonczarowska, J., Drummer, C., Fuchs, K., Ellinghaus, D., Kässens, J. C., Furholt, M., Kohlbacher, O., Schade-Lindig, S., Franke, A., Schreiber, S., … Krause-Kyora, B. (2021). Genome-wide study of a Neolithic Wartberg grave community reveals distinct HLA variation and hunter-gatherer ancestry. *Communications Biology*, *4*(1), 113. https://doi.org/10.1038/s42003-020-01627-4

## 2.27 Spiekeroog, Wittmund, Lower Saxony (Germany)

**Sample Used:**

SPI001 Site No.: 2212/2:1

**Contact information:** Jan F. Kegler

**Site information and excavation history:**

The site is located on the northern beach of the North Sea island of Spiekeroog about 3 km northeast of the town center. The find was discovered by volunteers (M. Huus) on 04.07.2016 and handed over to the Archaeological Service of the Ostfriesische Landschaft. No further archaeological finds could be made. The object comes from an undefined archaeological context. No follow-up investigation has been carried out on site.

**Summary of the sampled materials:**

Skeletal information: mandible, male, adolescent, min. 40 years. Grave goods: none. Additional information:

Wetland find of a human mandible, found during survey in 2016. The bone shows all the characteristics of wet soil preservation. Very robust mandible without knots, dentate with heavily abraded molars. Toothed with right P2, M1 and M2, left M1, M2 and M3. Carious dentition (interdental caries right P2).

An anthropological examination by Dr. S. Grefen-Peters, Braunschweig, confirmed the archaic character of the lower jaw, which comes from a man who probably died at the age of at least 40 years.

Isotope analyses by the University of Warsaw and the Curt Engelhorn Center for Archaeometry confirm a diet with a high content of marine food (probably fish, waterfowl and seal). The 87Sr/86Sr analyses from CEZA underline a local resident population. Genetic material has been extracted from the M2 Molar.

**Dating:**

### Table SI2.30 ^14^C dated samples from the Spiekeroog individual

| **ID number** | **Sample material** | **Lab code** | ^14^C Age BP ± 1 σ | **95.4% cal BCE** | **δ^13^C** |
| --- | --- | --- | --- | --- | --- |
| SPI001 Site No.: 2212/2:1 | molar | Poz-103001 | 6510 ± 40 BP | 5558-5373 | - |

**Source of the samples:** Archäologischer Dienst & Forschungsinstitut Ostfriesische Landschaft, Aurich

**Authors of the entry:** Jan F. Kegler

**References**:

Kegler, J.F., Grefen-Peters, S. (2019a): Männer aus dem Meer. Archäologie in Deutschland 02-2019, 59.

Kegler, J.F. u. Grefen-Peters, S. (2019b): Meermänner - Anthropologische Spülsaumfunde von Spiekeroog und Baltrum. Archäologie in Niedersachsen 22, 110-115.

## 2.28 Baltrum, Wittmund, Lower Saxony (Germany)

**Sample Used:**

BLR001 Site No.: 2210/5:2-1

**Contact information:** Jan F. Kegler

**Site information and excavation history:**

The site is located on the northern beach of the North Sea island of Baltrum about 4,5 km northeast of the town center. The find was discovered by volunteers (Chr. Groger) on 21.03 2018 and handed over to the Archaeological Service of the Ostfriesische Landschaft. No further archaeological finds could be made. The object comes from an undefined archaeological context. No follow-up investigation has been carried out on site

**Summary of the sampled materials:**

An anthropological examination by Dr. S. Grefen-Peters, Braunschweig, confirmed the archaic character of the lower jaw, which comes from an adult man who probably died at the age in between 20 to 50 years. Isotope analyses by the University of Warsaw and the Curt Engelhorn Center for Archaeometry (CEZA) confirm a diet with a high content of marine food (probably fish, waterfowl and seal). 87Sr/86Sr analyses from CEZA underline a local resident population.

Genetic material has been extracted from the M2 Molar.

The fragment of site 2210/5:2-1 (sample BLR001) is of a mandible that belonged to an adolescent male between 20-50 years old. There were no grave goods. The bone shows all the characteristics of wet soil preservation. The mandible fragment is very robust. The right side of the jaw is incomplete, the right branch and the corpus with the premolar and molar tooth fan are missing. Toothed with left M1, M2 and M3.

**Dating:**

### Table SI2.31 ^14^C date from the Baltrum individual

| **ID number** | **Sample material** | **Lab code** | ^14^C Age BP ± 1 σ | **95.4% cal BCE** | **δ^13^C** |
| --- | --- | --- | --- | --- | --- |
| BLR001 Site No.: 2210/5:2-1 | - | Poz-103000 | 4905 ± 30 | 3795-3655 | - |

**Source of the samples:** Archäologischer Dienst & Forschungsinstitut Ostfriesische Landschaft, Aurich

**Authors of the entry:** Jan F. Kegler

**References:**

Kegler, J.F., Grefen-Peters, S. (2019a): Männer aus dem Meer. Archäologie in Deutschland 02-2019, 59.

Kegler, J.F. u. Grefen-Peters, S. (2019b): Meermänner - Anthropologische Spülsaumfunde von Spiekeroog und Baltrum. Archäologie in Niedersachsen 22, 110-115.

# SI 3 Analytical details Sr-O-C isotope analysis

The dental elements selected for combined Sr-O-C isotope analysis (N=23) were mechanically cleaned at the Archaeological and Forensic Sample Preparation Laboratory of the Vrije Universiteit Amsterdam or, in case of ‘Cees’ from Opmeer-Mienakker (supplementary data 2.7), at the depot where the skeleton was housed, using a Proxxon drill equipped with a ball-shaped, acid-cleaned (10% HCl), diamond-coated grinding bit. Approximately 10 mg of white enamel powder was collected in clean glass vials.

For Sr isotope analysis, around 2 ± 1 mg of enamel powder was subsampled into acid-cleaned (6–7M HCl) Eppendorf® centrifuge tubes and transferred to the USA class 100 (ISO 5) clean laboratory, equipped with USA class 10 (ISO 4) laminar flow hoods at the same university. Additionally, 0.3 mg ± 10% was subsampled into clean, screw-capped Exetainer® vials and sent to the Stable Isotope Laboratory at the Vrije Universiteit Amsterdam for O-C isotope analysis.

Depending on the physical quality of the sample, the enamel powder underwent leaching with 0.1M acetic acid, followed by Milli-Q water rinsing and dissolution in 500 μl 3M HNO₃. Strontium extraction and sample loading were conducted following previously established protocols^116^. Isotope compositions were measured using a Thermo Scientific™ Triton Plus™ instrument housed at the Vrije Universiteit Amsterdam. Strontium ratios were determined via a static routine and corrected for mass fractionation to an ⁸⁶Sr/⁸⁸Sr of 0.1194. The long-term reproducibility was ± 0.000008 based on repeated analysis of the NIST® SRM® 987 standard during the course of the study (2019-2022: n = 393, 1σ, loading size: 200 ng). Procedural blanks (n = 7) contained between a negligible amount strontium. The ⁸⁷Sr/⁸⁶Sr are reported with a ±2 standard error (2SE), representing the analytical uncertainty derived from 240 measurements (12 blocks of 20 cycles) per run.

The δ¹⁸O and δ¹³C values were analyzed using a Thermo Finnigan GasBench II preparation device connected to a Thermo Finnigan Delta+ mass spectrometer. The data were normalized to the Vienna Peedee Belemnite (VPDB) scale using an in-house carbonate reference material (VICS), calibrated against NBS19 and LSVEC certified reference materials. Instrument performance was verified using the international control standard IAEA-603, which yielded average values of 2.40‰ for δ¹³C and −2.55 ‰ for δ¹⁸O (n = 31). The reproducibility of IAEA-603 during the analytical session was 0.12‰ (1σ).

# SI 4. *qpAdm* modeling of ancestry proportions

We used *qpAdm*^117^ to estimate ancestry proportions. We set the parameters allsnps: YES and inbreed: YES to account for the use of pseudo-haploid data.

Throughout this manuscript, we use terms such as “WHG”, “EHG”, “EEF” as genetic shorthand for ancestry components maximized in western European hunter-gatherers, eastern European hunter-gatherers, and Neolithic farmers of Anatolian origin, respectively. These labels refer solely to patterns of shared genetic ancestry and do not imply any specific subsistence strategy, cultural affiliation, or social identity for individuals in whom these components are detected.

Following Patterson *et al.*^118^, we used a core set of outgroups including:

- OldAfrica: a pool of diverse ancient African individuals with no evidence of recent West Eurasian-related admixture that we use as a deeply divergent outgroup.
- IronGates_HG: European hunter-gatherers that were genetically very similar to WHG but with slightly more Eastern European Hunter-Gatherer relatedness and mostly from the Iron Gates region of the Danube river in southeastern Europe
- Anatolia_N: Anatolian Neolithic farmers very similar genetically to Early European farmers (EEF).
- WSHG: West Siberian hunter-gatherers
- CHG_Iran_N: Caucasus hunter-gatherers (CHG) and Iran Neolithic individuals

We found that the addition of the last two populations to the outgroup set adds leverage to tease apart WHG-related ancestry, Eastern hunter-gatherer-related ancestry (EHG) and Steppe Early Bronze Age-related ancestry. Depending on the hypotheses being tested in each case, we complemented this core set by adding additional populations. We chose the nested fitting model if possible.

For target, source and outgroup populations outside of the Lower Rhine-Meuse area used throughout this section, the list of individuals included under each population label is provided in Supplementary Tables 3-4, together with PCA plots showing their genetic affinities and degree of ancestry heterogeneity (Extended Data Figures 1a-b)

The genetic data for the target Lower Rhine-Meuse individuals/groups, the source populations and the outgroup populations in our models were generated using different protocols (1240k capture, Twist capture and shotgun). Previous studies^119^ have reported bias when co-analysing different types of data, specially 1240k with both Twist or shotgun. Thus, to ensure that our results were not affected by this issue, we ran each *qpAdm* model with four different setups:

1) Using all 1240k autosomal SNPs.

2) Using 469k autosomal SNPs reported to greatly reduced the bias when co-analysing 1240k data with Twist and shotgun data^119^.

3) Using 711k autosomal SNPs with reduced bias identified using a novel approach (https://github.com/rmnfournier/compatibility-panel) to filter out biased SNPs^120^.

4) Using all 1240k autosomal SNPs but featuring only Twist or shotgun data in the target, source and outgroup populations (whenever possible) (Supplementary Table 4).

As shown in Supplementary Tables 5,6,9,10,11,12, we did not find any evidence that the major conclusions of this study are affected by bias resulting from co-analysing data generated by different protocols. Thus, in the following sections we report the P-values and ancestry proportions for the first qpADM setup.

**Mesolithic groups from the Lower Rhine-Meuse area**

We began by analyzing the ancestry in the 11 Mesolithic individuals from our area of interest, five of which were previously published^8^. Following Posth et al. 2023^8^ and Rivollat et al. 2020^121^, we modelled the Mesolithic individuals as a four-way mixture of Balkan_N+WHG+EHG+GoyetQ-2_cluster (Supplementary Table 5), representing the three major ancestries present in Mesolithic Europe together with an EEF rich to account for possible EEF-related admixture. We added GoyetQ116-1_cluster to the outgroup to increase leverage for detecting GoyetQ-2 related ancestry. The newly reported Lower Rhine-Meuse Mesolithic individuals can be modelled as 100% WHG (Villabruna/Oberkassel cluster), with no discernible Balkan_N, EHG or GoyetQ-2 related ancestry. This matches the results from previous publications and highlights that Mesolithic hunter-gatherers from this region derived all their ancestry from Villabruna/Oberkassel, unlike contemporaneous populations further east with EHG-related affinities, and further west with GoyetQ-2-related affinities.

**Early/Middle/Late Neolithic groups from the Lower Rhine-Meuse area**

We computed ancestry proportions for the model Balkan_N+WHG+EHG for each Neolithic individual separately and also after grouping individuals based on geography/chronology/cultural affiliation (Supplementary Tables 6). We excluded Late Neolithic Vlaardingen/Corded Ware-related individuals (RhineMeuse_LNA_Vlaardingen/CordedWare) and Bell Beaker-associated individuals (RhineMeuse_LNB_BB) (modelled in further sections). Given that GoyetQ-2_cluster-related ancestry has not been detected in any Mesolithic and Neolithic individual from central/northern Europe, we remove GoyetQ-2_cluster from the sources and replace GoyetQ116-1_cluster by Russia_Afanasievo in the outgroups, as this population increased our resolution to detect ancestry related to Steppe Early Bronce populations in our target individuals/populations.

Two EN_Swifterbant individuals who share a mother-daughter relation can be modelled successfully as 100% WHG. Most of the remaining individuals required a mixture between Balkan_N+WHG with high WHG proportions (~40-50%). The MN_Tiel individuals represent an exception with WHG values ~20%, more in line with the values observed in other central and western European regions. For a small subset of individuals, P-values increased when adding EHG as a third source (Supplementary Table 6), but the estimated proportions never surpass 10%.

To explore how the levels of hunter-gatherer ancestry observed in the Lower Rhine-Meuse area compare to other regions, we ran the *qpAdm* model Balkan_N+WHG+EHG for individuals dated between 4500-2500 BCE without steppe ancestry outside the Lower Rhine-Meuse area (Supplementary Table 7; Figure 2).

**Sex bias admixture in the Neolithic period**

Next, we wished to test for sex bias in the admixture between EEF and WHG ancestries. To increase resolution, we merged all the Neolithic individuals with EEF ancestry from the Lower Rhine-Meuse area (excluding the Late Neolithic ones with steppe ancestry) with capture data under one group (RhineMeuse_Neolithic) and tested the model Germany_EN_LBK+ WHG.

We ran this analysis for autosomal data only, and separately for the X-chromosome only (Supplementary Tables 8).

We used Germany Early Neolithic LBK farmers because they represent a more proximal source for the EEF-related ancestry (as opposed to Balkan_N), and our goal is to study evidence for sex-bias in the admixture that occurred in the Rhine-Meuse delta region, rather than studying an integrated signal that also might reflect admixture between people of Anatolian farmers and people of Mesolithic European hunter-gatherer ancestry as they interacted across the European continent prior to arriving in our study region.

If one of the two populations participating in the admixture event is predominantly composed by women, and the other population predominantly by men, we expect the ancestry proportions of the first population to be higher in the X-chromosome than those in the autosomes, as women carry two X-chromosomes for every one carried by men, while the autosomes are equally carried by both biological sexes. We find EEF-related proportions to be ~12% higher in the X-chromosome as compared to the autosomes (Supplementary Table 8), with a Z-score of 5 standard deviations above zero for the EEF ancestry being higher on the X-chromosome. This provides significant evidence of sex bias in the admixture event between Neolithic groups with EEF-related ancestry and hunter-gatherer groups, with EEF-related ancestry being predominantly contributed by females. Together with the independent evidence from mtDNA and Y-chromosome lineages, the data provide a compelling case for sex bias. To assess whether *qpAdm* ancestry estimates remain reliable when downsampling to the SNPs density of the X-chromosome, we computed ancestry proportions separately for each autosomal chromosome (Supplementary Table 8). Estimates for chromosomes with a similar number of Twist/1240k SNPs as the X-chromosome closely match the global autosomal proportions. For example, the 95% confidence interval for WHG ancestry on chromosome 9 (44.22-36.38%), the chromosome with the closest SNP count to the X-chromosome, includes the global autosomal estimate. Moreover, WHG proportions across all autosomes—regardless of SNP count—remain above 40%, whereas the X-chromosome estimate drops to 32%. This supports that the observed difference is not driven by data sparsity or noise.

**Corded Ware/Vlaardingen, Bell Beaker and Early Bronze Age groups from the Lower Rhine-Meuse area**

We applied the model Balkan_N+WHG+Germany_CordedWare to the individuals from the Corded Ware/Vlaardingen (RhineMeuse_LNA_Vlaardingen/CordedWare), Bell Beaker (RhineMeuse_LNB_BB) and Early Bronze Age (RhineMeuse_EBA) related groups, both individually and as groups (Supplementary Table 9).

Female individual I12896 from a Corded Ware/Vlaardingen context at Molenaarsgraaf could be modelled without Germany_CordedWare and with ~50% of WHG ancestry and Germany_CordedWare proportions not significantly different from 0, strongly suggesting that her ancestry derived from local Lower Rhine-Meuse populations with high levels of WHG. The remaining individuals provided good-fitting models for Balkan_N+WHG+Germany_CordedWare. The other two individuals from Corded Ware/Vlaardingen contexts displayed lower levels of Germany_CordedWare ancestry (13-21%), while this value for the RhineMeuse_LNB_BB and RhineMeuse_LNB_EBA groups reached ~80%. To investigate the origin of the non-CW part of the ancestry ( i.e. ancestry derived from Middle/Late Neolithic European groups without steppe ancestry) in RhineMeuse_LNA_Vlaardingen/CordedWare, RhineMeuse_LNB_BB and RhineMeuse_EBA groups, we first computed the ratio between the WHG ancestry proportion and the sum of WHG+Balkan_N ancestry proportions. This value informs us about the proportion of WHG related ancestry within the non-CW part of the ancestry of these groups. The RhineMeuse_Vlaardingen/CordedWare group (including only the two individuals with Germany_CordedWare ancestry) and the RhineMeuse_LNB_BB display ratios of 49% and 40%, respectively (Supplementary Table 9), very similar to the WHG levels observed in Midde-Late Neolithic populations from the Lower Rhine-Meuse area such as MN_Wartberg, MLN_Belgium or MN_Hazendonk, and hinting that they derived from a mixture between Corded Ware-related groups and local Neolithic groups from the Lower Rhine-Meuse region. The ratio for RhineMeuse_EBA, which displays slightly higher levels of non-CW ancestry as compared to Lower Rhine-Meuse BB, was 31%, lower than Lower Rhine-Meuse BB. This suggests that their extra non-CW ancestry is likely derived from MLN populations from outside the Lower Rhine-Meuse area with lower levels of WHG ancestry. For comparison, we run the same Balkan_N+WHG+Germany_CordedWare model for Bell Beaker individuals from England (England_BB), Czechia (Czechia_BB), southeast Germany (SEGermany_BB) and France (France_BB_Steppe and France_BB_NoSteppe), both individually and as groups (Supplementary Table 9). The ratio for England_BB was 37%, very similar to that obtain for RhineMeuse_LNB_BB. In contrast, SEGermany_BB and Czechia_BB, although having higher proportions of non-CW ancestry, show a ratio WHG/(WHG+EEF) of 22% and 23%, respectively, (Supplementary Table 9) that matches the WHG levels observed for populations such as Globular Amphora and TRB/Baalberge (see, for instance, Papac *et al.* 2021^122^ or Furtwängler *et al.* 2020^123^ for ancestry proportions in these groups). Similarly, France_BB_Steppe showed a ratio of 25%, which matches the levels of WHG ancestry observed in Late Neolithic groups from France without steppe ancestry such as Grotte du Rouquet, the main group from Mont-Aimé, Aven de la Boucle or Breviandes^124–126^

**Proximal modelling of the European Neolithic ancestry in** **Corded Ware/Vlaardingen, Bell Beaker and Early Bronze Age groups from the Lower Rhine-Meuse area**

Based on the observations in the previous section, we attempted to model the ancestry in RhineMeuse_LNA_Vlaardingen/CordedWare, RhineMeuse_LNB_BB and RhineMeuse_EBA (and groups from other regions) as a mixture between CW and Middle-Late Neolithic populations without steppe ancestry from different parts of Europe, either as two-way models CW+MLN group or three-way models CW+ CW+MLN group1+ CW+MLN group. We grouped Middle and Late Neolithic individuals from different parts of Europe into homogeneous groups that could represent plausible sources of ancestry (Supplementary Tables 3-4). We excluded Early Neolithic individuals because they are temporally distant from our target populations from the Rhine. Since we wanted to understand which Late Neolithic populations contributed ancestry to Lower Rhine-Meuse populations, we decided not to include Bell Beaker-associated individuals under any of these group labels, even when they lack steppe ancestry, as they are very close chronologically to our test populations. The Middle-Late Neolithic groups are the following:

-N_Spain_LNCA: we included individuals from northern Iberia dated to the Late Neolithic and Chalcolithic periods.

-NE_France_LN: we included Late Neolithic individuals from the northeast (Wettolsheim, Ferme de l'Ile, Mont-Aimé, Bréviandes les Pointes) with similar levels of HG ancestry^125–127^.

-S_France_LN: including Late Neolithic individuals from the south (Mas-Rouge, Peirières, Grotte du Rouquet, Aven de la Boucle)^125,127,128^ with similar levels of HG ancestry.

Germany_Baalberge_MN: Middle Neolithic Baalberge individuals from Germany.

-TRB_N: Neolithic TRB-associated individuals from the Czech Republic. In the case of the *qpAdm* setup with only Twist/shotgun data, we used TRB individuals from Denmark, Sweden and Poland.

-GlobularAmphora_LN: Late Neolithic Globular Amphora-associated individuals from Poland and Czechia.

-England_N: Neolithic individuals from England.

In addition to these, we also included Middle-Late Neolithic groups from the Lower Rhine-Meuse area (MLN_Belgium, MN_Wartberg, MN_ Hazendonk) as possible sources.

The RhineMeuse_LNA_Vlaardingen/CordedWare group could only be modelled using Lower Rhine-Meuse Neolithic sources MN_Hazendonk (84.4% ± 2%) or MLN_Belgium (87.7% ± 2%) in two-way models (Supplementary Table 10), which is consistent with the PCA results and the distal Balkan_N+WHG+Germany_CordedWare modelling (Supplementary Table 9). Three-way mixtures combining MLN_Belgium with one of the other MLN groups outside the Lower Rhine-Meuse area provided good-fitting models but with close-to-zero or negative proportions for these groups. The RhineMeuse_LNB_BB group could be modelled using two-way models including any of the three Lower Rhine-Meuse MLN groups (with ~17-18% ancestry), but not with MLN groups outside the Lower Rhine-Meuse. Unlike the Vlaardingen/CordedWare group, the three-way models assigned ancestry to both MLN_Belgium and the MLN group outside the Lower Rhine-Meuse area, with the former consistently receiving approximately twice as much (ranging from 10.9%-13% depending on the source). This suggests that the non-CW part of the ancestry in RhineMeuse_LNB_BB derives predominantly from the local MLN populations from the Lower Rhine-Meuse area with high hunter-gatherer ancestry.

Given the previously observed strong genetic similarity between Bell Beakers-associated individuals from the Lower Rhine-Meuse area and Bell Beaker-associated individuals from England^109^, we ran the same models for England_BB, which excludes four outlier individuals with higher levels of EEF ancestry (I14200, I1767, I2416, I5379). These four individuals (grouped as England_BB_highEEF) very likely harboured ancestry from local Neolithic from England and thus are not good representatives of the groups that moved to Britain at the beginning of the Chalcolithic period, and so we tested them as a separate group. The England_BB group behaved very similarly to RhineMeuse_LNB_BB and with matching ancestry proportions (Supplementary Table 10); i.e. non-fitting two way models when including MLN groups outside the Lower Rhine-Meuse area and a good fit for the two-way model including a MLN Lower Rhine-Meuse group (MN_Wartberg; 18% ancestry), and fitting three-way models with most of the MLN ancestry derived from the Lower Rhine-Meuse group (9.3-12%). These results again highlight the common origin between the main group of Bell Beaker-associated individuals from England and those from the Rhine-Meuse delta. In contrast, England_BB_highEEF show a very poor fit (P<3.12x10^-08^) for two-way models featuring Lower Rhine-Meuse MLN groups and much higher ancestry proportions (27-39.2%) assigned to the MLN outside the Rhine area in three way models, as compared to Lower Rhine-Meuse MLN groups (0-9%) very low ancestry proportions assigned to the groups in three-way models. Our interpretation is that that England_BB_highEEF, unlike the main England_BB group, likely represent recent mixtures with local Neolithic populations from Britain, and consequently most of their MLN ancestry component is best modeled by British Neolithic populations or other MLN groups with similar levels of hunter-gathered ancestry.

To check how Bell-Beaker associated groups from central Europe compare to RhineMeuse_LNB_BB and England_BB in this analysis, we tested the same models in the SEGermany_BB group. This group showed a very poor fit for two-way mixtures featuring MLN groups from the Lower Rhine-Meuse area, and the p-values substantially increase with MLN groups outside the Lower Rhine-Meuse area (Supplementary Table 10). In the three-way models, *qpAdm* assigned either negative or very low (<6%) ancestry proportions for the Lower Rhine-Meuse MLN group in most cases. These lines of evidence strongly suggest that the non-CW ancestry in RhineMeuse_LNB_BB and England_BB derived predominantly from MLN groups from the Lower Rhine-Meuse area with elevated levels of WHG ancestry, unlike Bell-Beaker associated groups from other regions such as southeast Germany, Czechia and France, which do not show signal of admixture with WHG-rich MLN groups.

Finally, the RhineMeuse_EBA group, whose lower ratio WHG/(WHG+EEF) already pointed to a slightly higher affinity to MLN groups with lower WHG ancestry, shows good-fitting two-way models for both MLN groups from the Lower Rhine-Meuse area (MN_Wartberg) and from other regions. The three-way models for the Lower Rhine-Meuse EBA groups support the previous observations and assign higher proportions (11.8-16.6%) for the MLN populations outside the Lower Rhine-Meuse area, but still with non-zero proportions for MLN_Belgium (5.8-10.1%) (Supplementary Table 10).

**Identification of the Lower Rhine-Meuse MLN source for RhineMeuse_LNA_Vlaardingen/CordedWare, RhineMeuse_LNB_BB**

Determining which specific MLN groups (both inside and outside the Lower Rhine-Meuse area) contributed ancestry to Lower Rhine-Meuse groups with Corded-Ware-related ancestry is very challenging because the MLN groups, especially those outside the Lower Rhine-Meuse area, harboured very similar levels of WHG ancestry. Since the three Lower Rhine-Meuse MLN groups display some differences in their WHG levels (47% for MLN_Belgium and MN_Hazendonk; 36% for MN_Wartberg) (Supplementary Table 6), we explored whether it was possible to determine if any of them was more likely to be the source of non-CW ancestry.

RhineMeuse_LNA_Vlaardingen/CordedWare could be modelled as MN_Hazendonk +Germany_CordedWare or MLN_Belgium+Germany_CordedWare with very similar ancestry proportions (Supplementary Table 10), not surprising given that, as mentioned above, MLN_Belgium and MN_Hazendonk are genetically very similar. The model with MN_Wartberg + Germany_CordedWare fails, likely because the MN_Wartberg has slightly lower WHG ancestry proportions than MLN_Belgium and MN_Hazendonk. For RhineMeuse_LNB_BB, their non-CW ancestry portion is much lower than that obtained for Vlaardingen/CordedWare (17% and ~84%, respectively) and thus, detecting the subtle differences in WHG levels is more challenging. Unlike the Vlaardingen/CordedWare group, RhineMeuse_LNB_BB could be well modelled with MN_Wartberg+ Germany_CordedWare. The model MN_Hazendonk + Germany_CordedWare also gives a good fit and the model MLN_Belgium + Germany_CordedWare yields a P-value that barely passes the 0.05 threshold. The same tendency of better fit for models including MN_Wartberg as compared to models including MLN_Belgium and MN_Hazendonk can be seen in the England_BB (Supplementary Table 10). This suggests that the non-CW source for the RhineMeuse_LNB_BB and England_BB groups likely had slightly lower WHG levels as compared to Vlaardingen/CordedWare, although still within the range of Lower Rhine-Meuse Neolithic populations, as using Neolithic populations outside the Lower Rhine-Meuse area with lower WHG proportions yields a very poor fit.

To further investigate this question, we tested a rotating approach^129^ using Germany_CordedWare as one source and either MN_Wartberg, MN_Hazendonk or MLN_Belgium as a non-CW source, including the other two in the outgroup set (Supplementary Table 11). If, for a given test population, one of the three non-CW sources is the true source, when placed in the outgroup set the model is expected to yield a poor fit, as this true source population in the outgroups will be related directly to the test population and not via the source populations. For Vlaardingen/CordedWare, the model MLN_Belgium+Germany_CW provided a good fit while MN_Hazendonk+Germany_CW and especially MN_Wartberg+Germany_CW yielded low P-values. In the case of RhineMeuse_LNB_BB, the model MN_Wartberg+Germany_CW resulted in the best fit, with MN_Hazendonk +Germany_CW and MLN_Belgium+Germany_CW yielding low p-values. This favours MLN_Belgium and MN_Wartberg as the most adequate non-CW sources for Lower Rhine-Meuse Vlaardingen/CordedWare-associated and Bell Beaker-associated groups, respectively.

**Proximal models to investigate the ancestry of Bell Beaker associated and Chalcolithic-Early Bronze groups from England**

So far, the England_BB group has shown strong similarities in qpADM modelling with RhineMeuse_LNB_BB. Further demonstrating cladality between the two, testing RhineMeuse_LNB_BB as the only source to model England_BB yields a P-value of 0.61 (Supplementary Table 12). When England_BB_highEEF or England_CA_EBA replace England_BB as the target group in this test, P-values dramatically drop to 1.64x10^-21^ and 2.79x10^-10^, indicating that England_BB_highEEF and England_CA_EBA are not cladal to RhineMeuse_LNB_BB and thus require additional ancestry. We then tried to improve the fit of the model by adding one or two MLN groups (Supplementary Table 12). In both cases, Lower Rhine-Meuse MLN groups are strongly rejected. The fit improves substantially when adding MLN groups with lower WHG levels, suggesting that the additional ancestry beyond RhineMeuse_LNB_BB is more likely derived from these groups. In the case of England_CA_EBA, models assigned 7.3-7.9% ancestry from these MLN groups. Since we cannot determine which specific MLN population was the actual source, there is a maximum of 8% ancestry derived from local Neolithic populations from England (if all the MLN ancestry beyond RhineMeuse_LNB_BB derived from England Neolithic) and a minimum of 0% (if all the MLN ancestry beyond RhineMeuse_LNB_BB derived from continental MLN groups).

**References**

1. Louwe Kooijmans, L. P. Schipluiden: a synthetic view. in *Schipluiden: A Neolithic Settlement on the Dutch North Sea Coast c. 3500 cal BC* (eds Louwe Kooijmans, L. P. & Jongste, P. F. B.) 485–516 (Leiden University Press, Leiden, 2006).

2. Reimer, P. J. *et al.* The IntCal20 Northern Hemisphere Radiocarbon Age Calibration Curve (0–55 cal kBP). *Radiocarbon* **62**, 725–757 (2020).

3. Toussaint, M. Les ossements humains du Mésolithique ancien de la grotte de Claminforge (Sambreville, province de Namur, Belgique). *Bulletin des Chercheurs de la Wallonie, Tome LIV* 251–281 (2019).

4. Toussaint, M. & Lacroix, P. Andenne/Thon: la Faille du Burin à Samson, une nouvelle sépulture collective du Mésolithique ancien. *Chronique de l’Archéologie Wallonne 10* 228–230 (2002).

5. Vos, P. C., van der Meulen, M., Weerts, H. J. T. & Bazelmans, J. *Atlas of the Holocene Netherlands, Landscape and Habitation since the Last Ice Age*. (Amsterdam University Press, Amsterdam, 2020).

6. Coles, B. J. Doggerland: a speculative survey. *Proceedings of the Prehistoric Society* **64**, 45–81 (1998).

7. Polet, C. & Cauwe, N. Les squelettes mésolithiques et néolithiques de l’abri des Autours (province de Namur, Belgique). *Comptes Rendus Palevol* **1**, 43–50 (2002).

8. Posth, C. *et al.* Palaeogenomics of Upper Palaeolithic to Neolithic European hunter-gatherers. *Nature* **615**, 117–126 (2023).

9. *Doggerland. Lost World under the North Sea*. (Sidestone Press, Leiden, 2022).

10. *Archeologie in de Betuweroute. Hardinxveld-Giessendam Polderweg: Een Mesolithisch Jachtkamp in Het Rivierengebied (5500-5000 v. Chr.)*. vol. 83 (NS Railinfrabeheer, Utrecht, 2001).

11. Smits, E. & Louwe Kooijmans, L. P. 13 Menselijke skeletresten. in *Archeologie in de Betuweroute. Hardinxveld-Giessendam De Bruin: Een kampplaats uit het Laat-Mesolithicum en het begin van de Swifterbant-cultuur (5500-4450 v.Chr.)* (eds Louwe Kooijmans, L. P., Koot, C. W., ten Anscher, T. J., Van Wijngaarden, G. J. & Goudswaard, B.) vol. 83 487–498 (Rijksdienst voor het Oudheidkundig Bodemonderzoek, Amersfoort, 2001).

12. Gaffney, V. L., Fitch, S. & Smith, D. N. *Europe’s Lost World: The Rediscovery of Doggerland*. (Council for British Archaeology, York, England, 2009).

13. Amkreutz, L. W. S. W. A view from Doggerland – interpreting the Mesolithic-Neolithic transition in the wetlands of the Rhine-Meuse delta (5,500 – 2,500 calBC). in *Stone Age borderland experience: Neolithic and Late Mesolithic parallel societies in the north European plain* (eds Klimscha, F., Heumueller, M., Raemaekers, D. C. M., Peeters, H. & Terberger, T.) 311–326 (Marie Leidorf GmbH, Rahden, 2022).

14. Walker, J. *et al.* A great wave: the Storegga tsunami and the end of Doggerland? *Antiquity* **94**, 1409–1425 (2020).

15. Ritchie, K. The Ertebølle Fisheries of Denmark, 5400-4000 B. (University of Wisconsin, Madison, 2010).

16. Andersen, S. H. ‘Køkkenmøddinger’ (Shell Middens) in Denmark: a Survey. *Proceedings of the Prehistoric Society* **66**, 361–384 (2000).

17. Enghoff, I. B. Freshwater fishing at Ringkloster, with a supplement of marine fishes. *Journal of Danish Archaeology* **12**, 99–106 (1995).

18. Raemaekers, D. *et al.* Timing and Pace of Neolithisation in the Dutch Wetlands (c. 5000–3500 cal. BC). *Open Archaeology* **7**, 658–670 (2021).

19. Brusgaard, N. Ø. *et al.* Early animal management in northern Europe: multi-proxy evidence from Swifterbant, the Netherlands. *Antiquity* **98**, 654–671 (2024).

20. Teetaert, D. & Crombé, P. The start of pottery production by hunter-gatherers in the Low Countries (Swifterbant Culture, 5th millennium BC) : a critical assessment of the available radiocarbon dates. *Notae Praehistoricae* **41**, 173–186 (2021).

21. Andersen, S. H. The first pottery in South Scandinavia. in *Pots, Farmers and Foragers. Pottery traditions and social interaction in the earliest Neolithic of the Lower Rhine Area* (eds Vanmontfort, B., Louwe Kooijmans, L. P., Amkreutz, L. W. S. W. & Verhart, L. B. M.) 167–213 (Leiden University Press, Leiden, 2010).

22. Dreshaj, M., Raemaekers, D. & Dee, M. Chronological modeling on a calibration plateau: implications for the emergence of agriculture in the Dutch wetlands. *Radiocarbon* **65**, 1280–1298 (2023).

23. Crombé, P. *et al.* New evidence on the earliest domesticated animals and possible small-scale husbandry in Atlantic NW Europe. *Sci Rep* **10**, 20083 (2020).

24. Hulst, R. S., Hogestijn, J. W. H., de Haan, M. J. A., Lauwerier, R. C. G. M. & Marswijk, R. W. Buren Zoelen. *Jaarverslag Rijksdienst voor het Oudheidkundig Bodemonderzoek 1992* 69 (1993).

25. Bakels, C. C. *The Western European Loess Belt : Agrarian History, 5300 BC - AD 1000*. (Springer Netherlands, Dordrecht, 2009).

26. Bakels, C. C. *Four Linearbandkeramik Settlements and Their Environment: A Paleoecological Study of Sittard, Stein, Elsloo and Hienheim*. vol. 11 (Leiden (proefschrift), 1978).

27. Kreuz, A. M. *Die Ersten Bauern Mitteleuropas - Eine Archäobotanische Untersuchung Zu Umwelt Und Landwirtschaft Der Ältesten Bandkeramik*. vol. 23 (Leiden (proefschrift), 1991).

28. Čerevková, A. The Subsistence Strategy of Linear Pottery Culture in Moravia (Czech Republic): Current State of Knowledge. **7**, 1473–1491 (2021).

29. Bakels, C. Archaeobotanical investigations in the Aisne valley, northern France, from the neolithic up to the early Middle Ages. *Veget Hist Archaebot* **8**, 71–77 (1999).

30. Denaire, A. *et al.* The Cultural Project: Formal Chronological Modelling of the Early and Middle Neolithic Sequence in Lower Alsace. *Journal of Archaeological Method and Theory* **24**, (2017).

31. Kirschneck, E. The Phenomena La Hoguette and Limburg – Technological Aspects. *Open Archaeology* **7**, 1295–1344 (2021).

32. Constantin, C., Illett, M. & Burnez-Lanotte, L. La Hoguette, Limburg and the Mesolithic: some questions. in *Pots, Farmers and Foragers. Pottery traditions and social interaction in the earliest Neolithic of the Lower Rhine Area* (eds Vanmontfort, B., Louwe Kooijmans, L. P., Amkreutz, L. W. S. W. & Verhart, L. B. M.) 41–49 (Leiden University Press, Leiden, 2010).

33. Hofmann, D. Keep on walking> the role of migration in Linearbandkeramik life. *Documenta Praehistorica 43* 235–251 (2016).

34. Crombé, P. Mesolithic projectile variability along the southern North Sea basin (NW Europe): Hunter-gatherer responses to repeated climate change at the beginning of the Holocene. *PLoS ONE* **14**, e0219094 (2019).

35. Crombé, P. & Cauwe, N. The Mesolithic. *Anthropologica et praehistorica* **112**, (2001).

36. Cauwe, N., Vander Linden, M. & Vanmontfort, B. The Middle and Late Neolithic. in *Prehistory in Belgium. Special issue on the occasion of the XIVth Congress of the International Union for Prehistoric and Protohistoric Sciences* 77–89 (SRBAP, Brussel, 2001).

37. van Berg, P. L. & Hauzeur, A. Le Néolithique ancien. *Anthropologica et Præhistorica 112* 63–76 (2001).

38. Kruk, J. *The Neolithic Settlement of Southern Poland*. (Archaeopress, Oxford, 1980).

39. Price, T. D. The introduction of farming in northern Europe. in *Europe’s First Farmers* (ed. Price, T. D.) 260–300 (Cambridge University Press, Cambridge, 2000). doi:10.1017/CBO9780511607851.011.

40. Regenye, J. *et al.* Narratives for Lengyel funerary practice. *Bericht der Römisch-Germanischen Kommission Bd. 97 2016(2020)* 5–80 (2020) doi:10.11588/DATA/2EVBVW.

41. Louwe Kooijmans, L. P. Mesolithic/Neolithic transformation in the lower Rhine basin. in *Case Studies in European Prehistory* (ed. Bogucki, P. I.) 95–145 (CRC Press, Boca Raton, 1993).

42. Fokkens, H., Steffens, B. J. W. & van As, S. F. M. *Farmers, Fishers, Fowlers, Hunters. Knowledge Generated by Development-Led Archaeology about the Late Neolithic, the Early Bronze Age and the Start of the Middle Bronze Age (2850 - 1500 Cal BC) in the Netherlands.* vol. 53 (Rijksdienst voor het Cultureel Erfgoed, Amersfoort, 2016).

43. Orschiedt, J., Gehlen, B., Schön, W. & Gröning, F. The Neolithic and Mesolithic Cave site ‘Blätterhöhle’ in Westphalia (D). *Notae Prehistoricae 32* 73–88 (2012).

44. Bollongino, R. *et al.* 2000 years of parallel societies in Stone Age Central Europe. *Science* **342**, 479–481 (2013).

45. Lipson, M. *et al.* Parallel palaeogenomic transects reveal complex genetic history of early European farmers. *Nature* **551**, 368–372 (2017).

46. *Doorbraken Aan de Rijn. Een Swifterbant-Gehucht, Een Hazendonk-Nederzetting En Erven En Graven Uit de Bronstijd in Medel-De Roeskamp*. (RAAP/Archol/ADC ArcheoProjecten/BAAC, Weesp/Leiden/Amersfoort/‘s-Hertogenbosch, 2023).

47. Louwe Kooijmans, L. P. & Jongste, P. F. B. *Schipluiden. A Neolithic Settlement on the Dutch North Sea Coast, c. 3500 Cal BVC*. vol. 37/38 (Faculty of Archaeology, Leiden, 2006).

48. Mol, J., Louwe Kooijmans, L. P. & Hamburg, T. D. 2 Stratigrahy and chronology of the site. in *Schipluiden: A Neolithic Settlement on the Dutch North Sea Coast c. 3500 cal BC* (eds Louwe Kooijmans, L. P. & Jongste, P. F. B.) 19–38 (Leiden University Press, Leiden, 2006).

49. Raemaekers, D. C. M. & Rooke, M. The Schipluiden pottery. in *Schipluiden: A Neolithic Settlement on the Dutch North Sea Coast c. 3500 cal BC* (eds Louwe Kooijmans, L. P. & Jongste, P. F. B.) 113–128 (Leiden University Press, Leiden, 2006).

50. ten Anscher, T. J. Leven met de vecht. schokland-p14 en de noordoostpolder in het neolithicum en de bronstijd. (Amsterdam University, Amsterdam, 2012).

51. Raemaekers, D. C. M. *et al.* The submerged pre-drouwen trb settlement site wetsingermaar, C. 3500 CAL. BC (province of Groningen, The Netherlands). *Palaeohistoria* **53**, 1–24 (2012).

52. Louwe Kooijmans, L. P. The Neolithic at the Lower Rhine. Its structure in chronological and geographical respect. *Dissertationes Archaeologicae Gandenses* **16**, 149–173 (1976).

53. Raemaekers, D. C. M. & de Roever, J. P. The Swifterbant pottery tradition (5000-3400 BC). Matters of fact and matters of interest. in *Pots, Farmers and Foragers. Pottery traditions and social interaction in the earliest Neolithic of the Lower Rhine Area* (eds Vanmontfort, B., Louwe Kooijmans, L. P., Amkreutz, L. W. S. W. & Verhart, L. B. M.) 135–149 (Leiden University Press, Leiden, 2010).

54. Vanmontfort, B. The Group of Spiere as a New Stylistic Entity in the Middle Neolithic Scheldt Basin. *Notae Praehistoricae* **21**, 139–143 (2001).

55. Vanmontfort, B. Can we attribute the middle Neolithic in the Scheldt and middle Meuse basins to the Michelsberg Culture? in *Impacts interculturels au Néolithique Moyen. du terroir au territoire: sociétés et espaces* (ed. Duhamel, P.) 109–116 (Artehis Éditions, Dijon, 2006).

56. Crombé, P., Boudin, M. & Van Strydonck, M. Swifterbant pottery in the Scheldt Basin and the emergence of the earliest indigenous pottery in the sandy lowlands of Belgium. in *Early pottery in the baltic - dating, origin and social context: International workshop at Schleswig from 20th to 21st October 2006* (eds Hartz, S., Lüth, F. & Terberger, T.) vol. Bericht der Römisch-Germanischen Kommission band 89 465–484 (Philipp von Zabern, Darmstadt, 2011).

57. Crombé, P. & Vanmontfort, B. The neolithisation of the Scheldt basin in western Belgium. in *Going Over: The Mesolithic-Neolithic Transition in North-West Europe* (eds Whittle, A. W. R. & Cummings, V.) 263–285 (The British Academy, London, 2007).

58. Cottiaux, R. & Salanova, L. Avant-propos : le Néolithique récent entre Seine, Oise et Marne. in *La fin du IVe millénaire dans le bassin parisien : Le Néolithique récent entre Seine, Oise et Marne (3500-2900 avant notre ère)* (eds Cottiaux, R. & Salanova, L.) 7–9 (ARTEHIS Éditions, Dijon, 2014).

59. Frébutte, C., Toussaint, M., Masy, P., Pirson, S. & Hubert, F. Campagne archéologique 2001 sur le site du «champ mégalithique de Wéris» à Durbuy (province de Luxembourg). *Notae Praehistoricae 21* 157–173 (2001).

60. Veselka, B. *et al.* Assembling Ancestors: the manipulation of Neolithic and Gallo-Roman skeletal remains from Pommeroeul, Belgium. *Antiquity* 1–16 (2024).

61. Immel, A. *et al.* Genome-wide study of a Neolithic Wartberg grave community reveals distinct HLA variation and hunter-gatherer ancestry. *Commun Biol* **4**, 113 (2021).

62. Modderman, P. J. R. The Neolithic burial vault at Stein. *Analecta Praehistorica Leidensia 1* 3–16 (1964).

63. Verhart, L. B. M. & Amkreutz, L. W. S. W. *Een Nieuwe Blik Op de Grafkelder van Stein*. (2017).

64. Amkreutz, L. W. S. W. Funerary practices on the fringe. The social dimensions of the Neolithic burial chamber of Stein and its European connections. in *The Early Neolithic of northern Europe. New approaches to migration, movement and social connection* (eds Hofmann, D., Cummings, V., Bjørnevad-Ahlqvist, M. & Iversen, R.) 21–34 (Sidestone Press, Leiden, 2025).

65. Koot, H., Bruning, L. & Houkes, R. *Ypenburg-Locatie 4: Een Nederzetting Met Grafveld Uit Het Midden Neolithicum in Het West-Nederlandse Kustgebied*. (Hazenbeg Archaeologie, Leiden, 2008).

66. Bakker, J. A. *The TRB West Group. Studies in the Chronology and Geography of the Makers of Hunebeds and Tiefstich Pottery*. (University of Amsterdam, Amsterdam, 1979).

67. Deichmüller, J. Die neolithische Moorsiedlung Hüde I am Dümmer, Kreis Grafschaft Diepholz, Vorläufiger Abschlussbericht. *Neue Ausgrabungen und Forschungen in Niedersachsen* **4**, 28–36 (1969).

68. Allentoft, M. E. *et al.* 100 ancient genomes show repeated population turnovers in Neolithic Denmark. *Nature* **625**, 329–337 (2024).

69. Iversen, R. The Pitted Ware Complex in a large scale perspective. *Acta Archaeologica* **81**, 5–41 (2010).

70. Iversen, R., Philippsen, B. & Persson, P. Reconsidering the Pitted Ware chronology: A temporal fixation of the Scandinavian Neolithic hunters, fishers and gatherers. *Praehistorische Zeitschrift* **96**, 44–88 (2021).

71. Coutinho, A. *et al.* The Neolithic Pitted Ware culture foragers were culturally but not genetically influenced by the Battle Axe culture herders. *American journal of physical anthropology* **172**, (2020).

72. Vanhanen, S. *et al.* Maritime Hunter-Gatherers Adopt Cultivation at the Farming Extreme of Northern Europe 5000 Years Ago. *Scientific Reports* **9**, 4756 (2019).

73. Louwe Kooijmans, L. P. *The Rhine/Meuse Delta; Four Studies on Its Prehistoric Occupation and Holocene Geology*. (Instituut voor Prehistorie, Leiden, 1974).

74. Cauwe, N. Les sépultures collectives néolithiques en grotte du Bassin mosan. Bilan documentaire. *Anthropologica et Præhistorica* **115**, 217–224 (2004).

75. Toussaint, M. *et al.* La Grotte Ambre à Matagne-la-Grande (Doische, Namur, Belgique) : étude anthropologique, biogéochimique et archéologique d’un amas d’ossements humains du Néolithique final du bassin mosan wallon. in *Deuxièmes Journées d’actualité de la recherche archéologique en Ardenne-Eifel Actes du colloque tenu à Viroinval 17-19 octobre 2019* (eds Smolderen, A. & Cattelain, P.) 63–100 (Centre d’Études et de Documentation Archéologiques (Cedarc), Treignes, 2020).

76. Haeck, J. La grotte du Mont Falise à Antheit, vallée de la Méhaigne, province de Liège. B 74: 39-54. *ulletin de la Société royale belge d’Anthropologie et de Préhistoire* **74**, 39–54 (1964).

77. Blanchet, J.-C. *Les Premiers Métallurgistes En Picardie et Dans La Nord de La France. Chalcolitique, Age Du Bronze et Début Du Premier Age Du Fer*. (CTHS, Paris, 1984).

78. Brunet, P. *et al.* La céramique de la fin du 4e et du 3e millénaire dans le Centre-Nord de la France: Bilan documentaire. in *Le troisième millénaire dans le nord de la France et en Belgique. Actes de la journée d’études SRBAP-SPF, 8 mars 2003, Lille* (eds Vander Linden, M. & Salanova, L.) 155–178 (SRBAP, Brussel, 2004).

79. Lambot, B. Le site chalcolithique du Gord à Compiègne (Oise) note préliminaire. *Cahiers Archéologique de Picardie* **8**, 5–18 (1981).

80. Cottiaux, R. La céramique du site éponyme du ‘Gord’ à Compiègne (Oise). *bspf* **92**, 97–106 (1995).

81. Blanchet, J.-C. & Lambot, B. Quelques aspects du Chalcolithique et du Bronze ancien en Picardie. *Revue Archéologique de Picardie* **3–4**, 79–118 (1985).

82. Martial, E., Praud, I. & Bostyn, F. Recherches récentes sur le Néolithique final dans le nord de la France. *Anthropologica et Præhistorica* **115**, 49–71 (2004).

83. Piningre, J.-F. Un aspect de la fin du Néolithique dans le Nord de la France. Les sites de Seclin, Houplin-Ancoisne et Saint-Saulve (Nord). *pica* **3**, 53–69 (1985).

84. Demeyere, F., Bourgeois, J. & Crombé, P. Plan d’une maison du groupe de Deûle-Escaut à Waardamme (Oostkamp, Flandre occidentale). (2004).

85. Oueslati, T., Leroy, G. & Salvador, P.-G. Fowling on the banks of the Scheldt river in the recent Neolithic (France, 3300-2900 cal BC). *Quaternary International* **626–627**, 52–61 (2022).

86. Praud, I. *et al.* *Le Néolithique Final Dans La Vallée de La Deûle. Le Site d’Houplin-Ancoisne, Le Marais de Santes.* vol. Recherches archéologiques 9 (CNRS Éditions; Inrap, 2015).

87. Sergant, J. *et al.* Een tweede vindplaats van de Deûle-Escaut groep in de Vlaamse zandstreek De site van Hertsberge – Papenvijvers 3 (gem. Oostkamp, West-Vlaanderen, België). *Notae Praehistoricae* **29**, 93–99 (2009).

88. Salanova, L. *et al.* Du Néolithique récent à l’âge du Bronze dans le centre nord de la France : les étapes de l’évolution chrono-culturelle. in *Le Néolithique du Nord de la France dans son contexte européen : habitat et économie aux 4e et 3e millénaires avant notre ère. Actes du 29e colloque interrégional sur le Néolithique Villeneuve-d’Ascq 2-3 octobre 2009* (eds Bostyn, F., Martial, E. & Praud, I.) vol. Revue archéologique de Picardie. Numéro spécial 28 77–102 (2011).

89. Delcourt-Vlaeminck, M. Les exportations du silex du Grand-Pressigny et du matériau tertiaire dans le nord-ouest de I’Europe au Néolithique final / Chalcolithique. *Anthropologica et Præhistorica* **115**, 139–154 (2004).

90. Mallet, N., Ihuel, E. & Verjux, C. La diffusion des silex du Grand-Pressigny au néolithique / Diffusion of Grand-Pressigny flint during Neolithic. *Supplément à la Revue archéologique du centre de la France* **38**, 131–147 (2012).

91. Ihuel, E., Mallet, N., Pelegrin, J. & Verjux, C. The dagger phenomenon: circulation from the Grand-Pressigny region (France, Indre-et-Loire) in Western Europe. in *The Bell Beaker transition in Europe. Mobility and local evolution during the 3rd Millennium BC* (eds Prieto Martinez, M. P. & Salanova, L.) 113–126 (Oxbow Books, Oxford, 2015).

92. Fokkens, H. The structure of Late Neolithic and Early Bronze Age settlements and houses in the Netherlands. in *Siedlungsarchäologie des Endneolithicums und der frühen Bronzezeit. 11. Mitteldeutsche Arcäologentag vom 18. bis 20. Oktober 2018 in Halle (Saale)* (eds Meller, H., Friederich, S., Küssner, M., Staüble, H. & Risch, R.) 915–936 (Landesmuseum für Vorgeschichte, Halle, 2019).

93. Martial, E. & Praud, I. Une nouvelle occupation du Néolithique final dans le Nord, à Baisieux : présentation liminaire. *InterNéo 12* 127–138 (2018).

94. Nobles, .G. R. 3. Features. in *A Mosaic of Habitation at Zeewijk (the Netherlands) Late Neolithic Behavioural Variability in a Dynamic Landscape* (eds Theunissen, E. M., Brinkkemper, O., Lauwerier, R. C. G. M., Smit, B. I. & Van der Jagt, I. M. M.) 39–54 (Cultural Heritage Agency of the Netherlands, Amersfoort, 2014).

95. van Kampen, J. C. G. & van den Brink, V. B. *Archeologisch Onderzoek Op de Habraken Te Veldhoven. Twee Unieke Nederzettingen Uit Het Laat Neolithicum En de Midden Bronstijd En Een Erf Uit de Volle Middeleeuwen*. (2013).

96. Toussaint, M. Les sépultures mésolithiques du bassin mosan wallon: où en est la recherche en 2010? *Les sépultures mésolithiques du bassin mosan wallon: où en est la recherche en 2010?* 69–89 (2010).

97. de Groote, I. *et al.* Report on the latest excavation campaigns at Grotte de La Faucille, Sclayn (BE) : new radiocarbon dates for a better understanding of burial practice during the Final Neolithic. *(2022) NOTAE PRAEHISTORICAE* 161–177 (2022).

98. Kroon, E. J. *Serial Learners. Interactions between Funnel Beaker West and Corded Ware Communities in the Netherlands during the Third Millennium BCE from the Perspective of Ceramic Technology*. (Sidestone Press, Leiden, 2024).

99. Bourgeois, Q. P. J., Kroon, E. J. & Olerud, L. S. Parallel societies: evidence for the co-existence of Late Funnel Beaker West and Early Corded Ware communities, in. in *The Eve of Destruction? Local groups and large-scale networks during the late fourth and early third millennium BC in central Europe* (eds Hofmann, D., Mischka, D. & Scharl, S.) (Sidestone Press, Leiden, 2025).

100. Beckerman, S. *Corded Ware Coastal Communities. Using Ceramic Analysis to Reconstruct Third Millennium BC Societies in the Netherlands*. (Sidestone Press, Leiden, 2015).

101. Kroon, E. J., Huisman, D. J., Bourgeois, Q. P. J., Braekmans, D. J. G. & Fokkens, H. The introduction of Corded Ware Culture at a local level: An exploratory study of cultural change during the Late Neolithic of the Dutch West Coast through ceramic technology. *Journal of Archaeological Science: Reports* **26**, 101873 (2019).

102. Furholt, M. Mobility and social change: understanding the european Neolithic period after the archaeogenetic revolution. *J Archaeol Res* **29**, 481–535 (2021).

103. Lanting, J. N. De NO-Nederlandse/NW-Duitse Klokbekergroep: Culturele achtergrond, typologie van het aardewerk, datering, verspreiding en grafritueel. *Palaeohistoria* **49/50 (2007-2008)**, 11–326 (2008).

104. Needham, S. P. Transforming Beaker Culture in North-West Europe; Processes of Fusion and Fission. *Proceedings of the Prehistoric Society* **71**, 171–217 (2005).

105. Fitzpatrick, A. P. The arrival of the Beaker Set in Britain and Ireland: rethinking the Bronze Age and the arrival of Indo-European in Atlantic Europe. in *Celtic from the West 2* (eds Koch, J. T. & Cunliffe, B. W.) 41–70 (Oxbow books, Oxford, 2013).

106. Lanting, J. N. & van der Waals, J. D. *Glockenbecher Symposium Oberried 1974*. (Fibula-Van Dishoeck, Bussum, 1976).

107. Wentink, K. *Stereotype: The Role of Grave Sets in Corded Ware and Bell Beaker Funerary Practices*. (Sidestone Press, Leiden, 2020).

108. Fokkens, H., Veselka, B., Bourgeois, Q., Olalde, I. & Reich, D. Excavations of Late Neolithic arable, burial mounds and a number of well-preserved skeletons at Oostwoud-Tuithoorn; a re-analysis of old data. *Analecta Praehistorica Leidensia* **47**, 95–150 (2017).

109. Olalde, I. *et al.* The Beaker phenomenon and the genomic transformation of northwest Europe. *Nature* **555**, 190–196 (2018).

110. Lohof, E., Hamburg, T. & Flamman, J. *Steentijd Opgespoord. Archeologisch Onderzoek in Het Tracé van de Hanzelijn-Oude Land*. vol. Archol rapport 138 & ADC rapport 2576 (Archol bv & ADC ArcheoProjecten bv, Amersfoort, 2011).

111. Besse, M. Bell Beaker Common Ware during the third Millennium BC in Europe. in *Similar but different. Bell beakers in Europe* (ed. Czebreszuk, J.) 127–148 (Adam Mickiewicz University, Poznan, 2004).

112. Arnoldussen, S. *A Living Landscape. Bronze Age Settlement Sites in the Dutch River Area (*c. *2000-800 BC)*. (Sidestone Press, Leiden, 2008).

113. Philippsen, B. The freshwater reservoir effect in radiocarbon dating. *Heritage Science* **1**, 24 (2013).

114. van der Plicht, J. & Streurman, H. J. A new model for radiocarbon dating of marine shells from the Netherlands. *Radiocarbon* **67**, 378–411 (2025).

115. Dreshaj, M., Dee, M., Brusgaard, N., Raemaekers, D. & Peeters, H. High-resolution Bayesian chronology of the earliest evidence of domesticated animals in the Dutch wetlands (Hardinxveld-Giessendam archaeological sites). *PLOS ONE, 18(1), e0280619* https://doi.org/doi.org/10.1371/journal.pone.0280619 (2023) doi:doi.org/10.1371/journal.pone.0280619.

116. Kootker, L. M. & De Coster, M. R. A. L. Chromatographic separation of strontium in archaeological human and faunal enamel for Thermal Ionisation Mass Spectrometry (TIMS) analysis. protocols.io. https://dx.doi.org/10.17504/protocols.io.bp2l628nkgqe/v1 (2024) doi:https://dx.doi.org/10.17504/protocols.io.bp2l628nkgqe/v1.

117. Haak, W. *et al.* Massive migration from the steppe was a source for Indo-European languages in Europe. *Nature* **522**, 207–211 (2015).

118. Patterson, N. *et al.* Large-scale migration into Britain during the Middle to Late Bronze Age. *Nature* **601**, 588–594 (2021).

119. Rohland, N. *et al.* Three assays for in-solution enrichment of ancient human DNA at more than a million SNPs. *Genome Res.* **32**, 2068–2078 (2022).

120. Fournier, R., Fulton, A. P. & Reich, D. A SNP panel for co-analysis of capture and shotgun ancient DNA data. *bioRxiv* https://doi.org/10.1101/2025.07.30.667733 (2025).

121. Rivollat, M. *et al.* Ancient genome-wide DNA from France highlights the complexity of interactions between Mesolithic hunter-gatherers and Neolithic farmers. *Sci. Adv.* **6**, eaaz5344 (2020).

122. Papac, L. *et al.* Dynamic changes in genomic and social structures in third millennium BCE central Europe. *Sci. Adv.* **7**, eabi6941 (2021).

123. Furtwängler, A. *et al.* Ancient genomes reveal social and genetic structure of Late Neolithic Switzerland. *Nat Commun* **11**, 1915 (2020).

124. Arzelier, A. *et al.* Neolithic genomic data from southern France showcase intensified interactions with hunter-gatherer communities. *iScience* **25**, 105387 (2022).

125. Seguin-Orlando, A. *et al.* Heterogeneous Hunter-Gatherer and Steppe-Related Ancestries in Late Neolithic and Bell Beaker Genomes from Present-Day France. *Current Biology* **31**, (2021).

126. Parasayan, O. *et al.* Late Neolithic collective burial reveals admixture dynamics during the third millennium BCE and the shaping of the European genome. *Science Advances* **10**, eadl2468 (2024).

127. Brunel, S. *et al.* Ancient genomes from present-day France unveil 7,000 years of its demographic history. *Proc Natl Acad Sci USA* **117**, 12791–12798 (2020).

128. Arzelier, A., Binder, D., Duday, H., Deguilloux, M.-F. & Pruvost, M. Ancient DNA sheds light on the funerary practices of late Neolithic collective burial in southern France. https://doi.org/10.1098/rspb.2024.1215 (2024) doi:https://doi.org/10.1098/rspb.2024.1215.

129. Harney, É., Patterson, N., Reich, D. & Wakeley, J. Assessing the performance of qpAdm: a statistical tool for studying population admixture. *Genetics* **217**, iyaa045 (2021).
